# Mining Predicted Essential Genes of *Brugia malayi* for Nematode Drug Targets

**DOI:** 10.1371/journal.pone.0001189

**Published:** 2007-11-14

**Authors:** Sanjay Kumar, Kshitiz Chaudhary, Jeremy M. Foster, Jacopo F. Novelli, Yinhua Zhang, Shiliang Wang, David Spiro, Elodie Ghedin, Clotilde K. S. Carlow

**Affiliations:** 1 Division of Parasitology, New England Biolabs, Inc., Ipswich, Massachusetts, United States of America; 2 The Institute for Genomic Research, Rockville, Maryland, United States of America; 3 Division of Infectious Diseases, University of Pittsburgh School of Medicine, Pittsburgh, Pennsylvania, United States of America; Centre for DNA Fingerprinting and Diagnostics, India

## Abstract

We report results from the first genome-wide application of a rational drug target selection methodology to a metazoan pathogen genome, the completed draft sequence of *Brugia malayi,* a parasitic nematode responsible for human lymphatic filariasis. More than 1.5 billion people worldwide are at risk of contracting lymphatic filariasis and onchocerciasis, a related filarial disease. Drug treatments for filariasis have not changed significantly in over 20 years, and with the risk of resistance rising, there is an urgent need for the development of new anti-filarial drug therapies. The recent publication of the draft genomic sequence for *B. malayi* enables a genome-wide search for new drug targets. However, there is no functional genomics data in *B. malayi* to guide the selection of potential drug targets. To circumvent this problem, we have utilized the free-living model nematode *Caenorhabditis elegans* as a surrogate for *B. malayi*. Sequence comparisons between the two genomes allow us to map *C. elegans* orthologs to *B. malayi* genes. Using these orthology mappings and by incorporating the extensive genomic and functional genomic data, including genome-wide RNAi screens, that already exist for *C. elegans*, we identify potentially essential genes in *B. malayi*. Further incorporation of human host genome sequence data and a custom algorithm for prioritization enables us to collect and rank nearly 600 drug target candidates. Previously identified potential drug targets cluster near the top of our prioritized list, lending credibility to our methodology. Over-represented Gene Ontology terms, predicted InterPro domains, and RNAi phenotypes of *C. elegans* orthologs associated with the potential target pool are identified. By virtue of the selection procedure, the potential *B. malayi* drug targets highlight components of key processes in nematode biology such as central metabolism, molting and regulation of gene expression.

## Introduction

The arrival of the post-genomic era has brought with it the possibility of *in silico* selection of drug targets in major human pathogens using rational target-based approaches. Soon after the first microbial genomes were sequenced, comparative and subtractive genomic strategies were proposed to isolate potential drug targets from an organism's complete catalog of gene products. Probable essentiality could be inferred from inter-genomic sequence conservation [1], and possible lead compound toxicity could be disfavored by focusing on targets that lack close homologs in mammals [1], [2]. For many bacterial genomes, functional data is now available enabling direct identification of essential genes and has been incorporated into the approach [3]. Unfortunately, for metazoan pathogens, including human helminth parasites, there is a dearth of complete genomic sequences. To complicate matters further, many parasites are genetically intractable, making gene functions difficult to establish experimentally. However, by using a related model organism as a proxy for missing functional genomic data and applying multiple layers of subtractive filters based on comparative sequence analysis, we can pre-validate a pool of targets to facilitate their entry into drug discovery programs. This methodology was tested successfully in parasitic nematodes, albeit incompletely as only fragmentary EST sequence data was available [4], [5], and has been endorsed by the World Health Organization as a promising approach to identify new helminth drug targets [6].

Worldwide, helminth parasites result in a combined conservative disease burden of 8 million DALYs (Disability Adjusted Life Years) [7]. Lymphatic filariasis and onchocerciasis are tropical diseases caused by filarial parasites that are transmitted to humans by insects. Collectively, they afflict approximately 150 million people in over 80 countries with more than 1.5 billion at risk of infection [7]. The mainstay of filarial disease control for several decades has been a limited number of drugs, predominantly diethylcarbamazine, benzimidazoles (e.g. albendazole) and avermectins (e.g. ivermectin) [8]. Ivermectin exerts its anthelmintic effect by modulating the activity of glutamate-gated chloride channel while albendazole binds to tubulin so as to inhibit its polymerization and the subsequent formation of microtubules. The mode of action of DEC is still not understood [8]. These compounds suffer various drawbacks such as not being effective against all stages of the parasite, the requirement for annual or semi-annual administration, possible side effects and contra-indications for certain individuals. Furthermore, signs of emerging drug resistance are becoming increasingly apparent [9], [10]. Therefore novel chemotherapeutics and vaccines are urgently needed.

In this report, we describe the results from the first application of the *in silico* filtering methodology to a metazoan parasite genome, the completed draft sequence of *Brugia malayi* [11]. We have expanded our previous analysis, which was limited to nematode ESTs [4], and applied this methodology to the complete gene complement predicted for this organism. By incorporating a custom ranking algorithm, we were able to identify and prioritize a pool of 589 potential targets for further study. We also discuss the significance of those candidate targets in terms of nematode biology.

## Results and Discussion

Filarial parasites are related to the free-living nematode *Caenorhabditis elegans,* a model organism with a fully sequenced and extensively annotated genome. Multiple independent genome-wide analyses of gene function for nearly all ∼20000 *C. elegans* genes have been undertaken using high-throughput RNA interference (RNAi). This data, comprising ∼61000 entries, is publicly accessible via Wormbase [12]. The set of genes with non-wild type phenotypes in RNAi screens constitutes a pool of phenotypically significant and potentially essential *C. elegans* genes. We reasoned that homologs of these genes in *B. malayi* are also likely to be essential. *C. elegans* is generally believed to be a valid model for less genetically tractable parasitic nematodes [13]–[15]. Indeed, there is good concordance between the phenotypes resulting from the few cases where genes from filarial nematodes have been targeted by RNAi and similar experiments targeting their *C. elegans* orthologs [16]–[19].

**Figure 1:**
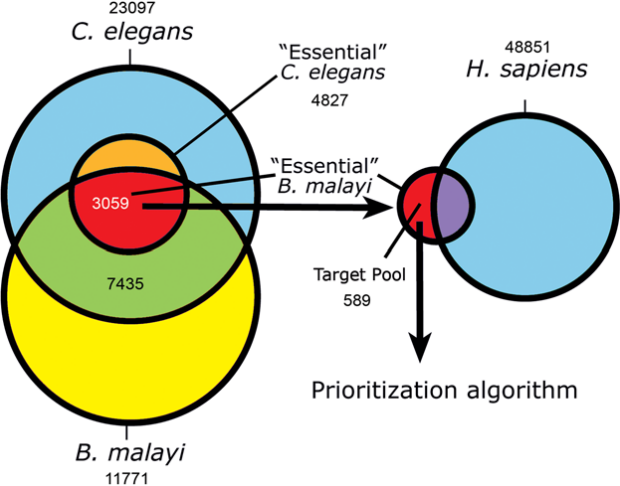
Selection methodology. Venn diagrams summarizing the reduction in search space achieved by selection of “essential” *B. malayi* gene products (left), and subsequent refinement of targets (right). Overlapping areas (not to scale) represent orthologous sequences (Materials and Methods). Numbers indicate gene products.

Using release 150 of Wormbase (http://www.wormbase.org), we recovered 4827 *C. elegans* genes with non-wild type RNAi phenotypes (RNAi positive set). From the 11771 predicted gene products in the data snapshot of the *B. malayi* genome used in our studies, we identified 7435 as having an ortholog in *C. elegans* (Materials and Methods). Of these, 3059 were mapped to the RNAi positive set, constituting a predicted “essential” *B. malayi* genome. The majority of these essential genes have close human homologs and were removed. The remainder is a set of 589 first-pass candidate drug targets (Fig. 1, Table S1).

Analysis of protein domains in the target set shows the presence of several over-represented domains as compared to the whole genome (Table S2), suggestive of an important role in nematode biology. The C2H2 type zinc-finger domain and basic helix-loop-helix dimerization domain are over-represented 3- and 4-fold respectively in the target list, as compared to the whole genome, indicative of proteins that bind to nucleic acids and are presumably involved in essential gene regulation and developmental pathways in the parasite. The collagen triple helix repeat, over-represented by 5-fold, reflects unique components of the cuticle and extracellular matrix. Twenty-four potential targets contain InterPro domains that can be mapped to 14 distinct Enzyme Commission (E.C.) numbers (Table S3). Functional classification of the target set using gene ontology (GO) annotations (Table S4) and statistical analysis of the GO term content (Table 1) revealed several over-represented terms including cuticle structure and ion transport.

**Table 1:** Over-represented GO terms in the target pool.

GO Hierarchy	GO Term ID	GO Term	Freq. in Geneome	Freq. in Targets
**Cellular Component**	GO:0005737	Cytoplasm	349	41
	GO:0005739	Mitochondria	31	10
	GO:0030054	Cell junction	13	7
	GO:0005911	Intracellular junction	13	7
	GO:0005921	Gap junction	13	7
**Molecular Function**	GO:0005198	Structural molecule activity	192	24
	GO:0042302	Structural constituent of cuticle	46	17
	GO:0015077	Monovalent inorganic cation activity	32	11
	GO:0015078	Hydrogen ion transporter activity	32	11
**Biological Process**	GO:0006811	Ion transport	233	30
	GO:0006820	Anion transport	91	18
	GO:0015698	Inorganic anion transport	85	18
	GO:0006817	Phosphate transport	81	18

GO term over representation was calculated as described in Materials and Methods. A minimum significance of p<0.05 was required. The fractions indicate the frequency of the term in the entire predicted *B. malayi proteome* (of 11771) and the target pool (of 589), respectively.

While the pool of 589 candidates reflects a 20-fold reduction in the search space, it is still too large to enter drug-screening pipelines. To rank the output and identify the most promising potential targets, we developed a computational algorithm for integrating and weighting the biological data from *C. elegans* and *B. malayi* (Table 2). The aim of the prioritization algorithm was to predict the efficacy, selectivity and tractability of each candidate target. Hasan *et al.* recently used a similar approach for prioritizing potential drug targets in *Mycobacterium tuberculosis* [20].

**Table 2:** Prioritization factors and relative weighting scheme.

Criteria	Description	Weight	Observed Range
Homology and protein length ratio^a^	Present in *C. elegans*	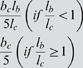	0…226
	Present in *H. sapiens*	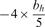	−61…0
Essentiality^b^	Severity and reproducibility of the RNAi phenotype of the *C. elegans* ortholog	**+** 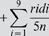	0…230
Stage specific expression	Presence of specific ESTs in all stages (microfilariae, L2, L3, L4 and adults)^c^	+10	0…10
	Presence of ESTs in adults^c^	+7	
	Presence of ESTs in L4^c^	+5	
	Presence of ESTs in L1^c^	+4	
	Presence of ESTs in L3^c^	+3	
	Presence of ESTs in L2^c^	+1	
Druggability	Presence of LR5 druggable domain	+50	0…50
	Presence of druggable E.C. number	+50	0…50
Expressability^d^	GRAVY score measuring hydropathicity and expressability	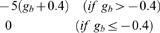	−21…0

Total scores (range −52 … 275) for each target were obtained by summing the individual weights.

a *b_c_*, maximum bit score of the *B. malayi* : *C. elegans* protein alignment; *b_h_*, maximum bit score of the *B. malayi : H. sapiens* protein alignment; *l_b_, l_c_*, number of amino acids in *B. malayi* and *C. elegans* homologs respectively.

b *r_i_*, number of instances an RNAi phenotype has been reported for the gene in wormbase; *d_i_,* degree of severity (0–100) assigned to a phenotype bin *i*; n, number of RNAi experiments reported for a particular gene.

c maximum value, irrespective of expression data in other stages/categories (non-additive).

d *g_b_*, GRAVY score for the *B. malayi* protein.

**Figure 2:**
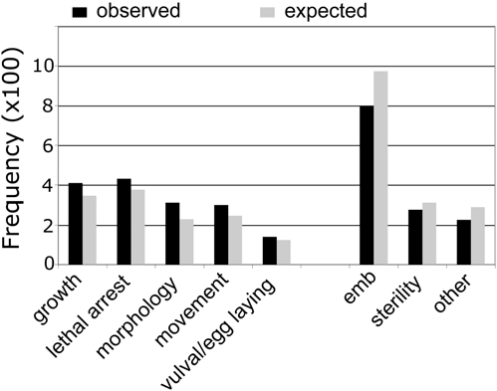
Frequencies of binned RNAi phenotypes in *C. elegans* orthologs of *B. malayi* targets. Observed frequencies were all statistically over- or under-represented relative to expected frequency in the whole genome based on a hypergeometric distribution (P values<1e-3). The entire set of observed values was statistically different from the background (expected) values as measured by a G-test (two sided P value = 5.9e-21).

Potential targets were rewarded for high sequence similarity with *C. elegans* orthologs, but penalized heavily for the presence of a close homolog in humans. Based on the protein length ratios of the orthologs, we identified and penalized *B. malayi* gene models that were incomplete or fragmented. Examples of such gene models include two previously proposed drug targets, 2,3-bisphosphoglycerate-independent phosphoglycerate mutase [21] (model 13047.m00009) and chitin synthase 2 [22] (models 12621.m00166 and 14328.m00023) respectively; despite being penalized, these gene models appear in the top half of the ranked list based on their high scores in other positive ranking criteria. In some instances, manual prediction of the complete coding region revealed strong similarity to human proteins which was not detected using the incomplete or fragmented models. RNAi phenotype data for *C. elegans* (obtained from Wormbase) was used to prioritize *B. malayi* orthologs with respect to their potential efficacy. All reported *C. elegans* RNAi phenotypes were binned into nine categories and assigned weights based on the severity of the observed phenotype (see Methods and Table S5). Adult/larval lethality/arrest was assigned the highest weight. Replicating the adult lethality phenotype would be an important first step towards developing an effective and much-needed macrofilaricide (compound targeting adult worms). To overcome the complications arising from false positives we used ‘phenotype redundancy’ [23] as a measure of confidence, in which independent experiments using different reagents targeting a single gene produce the same phenotype. The product of severity and redundancy for each phenotype category was summed up and normalized by the total number of RNAi experiments for each gene to provide an aggregate confidence score. Interestingly, when the frequency distribution of the binned RNAi categories for *C. elegans* sequences orthologous to the target pool was compared with that expected from the whole genome, we observed that reproductive and embryonic phenotypes (sterility and embryonic arrest/lethality) associated with genes involved in highly conserved metazoan processes were under-represented, whereas post-embryonic phenotypes were slightly over-represented (Fig 2). The latter bodes well for our attempts to prioritize drug targets for larvicidal and macrofilaricidal discovery.

Targets were also prioritized based on data for stage specific expression from approximately 24000 ESTs derived from various stage and gender specific *B. malayi* libraries [24]. Of 589 targets, 252 had corresponding EST sequences. We compiled expression data from microfilariae (L1), L2, L3, L4 and adult stages of the parasite and assigned highest weight to targets which have evidence of expression in all five stages. Next were targets that are expressed in the adults, L4, L1, L3 and L2 stage, in decreasing order of priority.

Other important prioritization criteria included predicted ‘druggability’ and expressability. Druggability can be described as the presence of protein folds that favor interactions with drug-like chemical compounds. Hopkins *et al* identified 130 InterPro protein domains that are targeted by established and experimental small molecule drugs that follow the Lipinsky rule of 5 (LR5) [25]. Similarly, a list of 70 EC numbers of known enzyme targets and respective marketed drugs was compiled [26]. Proteins with LR5 druggable domains or druggable EC numbers were given a high priority. An important factor for selection of targets for rational drug design is their potential to be expressed in heterologous systems for protein production, purification and crystallization. A genome wide survey for high throughput expression of *C. elegans* proteins in *Escherichia coli* found that protein expression and solubility are inversely correlated with hydrophobicity. Proteins having GRAVY (grand average of hydropathicity) scores below an empirically derived cutoff of −0.4 were more likely to be soluble [27]. To prioritize drug targets in *B. malayi*, we penalized proteins with a GRAVY score higher than −0.4. A complete set of data values used for prioritizing the potential targets are available in Supplementary Data Set S1.

The ranked output (Tables 3 and S1), sorted by the sum of the individual scores for each predicted target, was then manually curated to improve functional annotations where possible. Twelve known or previously proposed targets were identified; nine of these are among the top 40 targets shown in Table 3, endorsing the validity of our approach. Two potential targets, triacylglycerol lipase and adenosine deaminase, having domains associated with druggable enzymes and ten targets with LR5 domains, including the rhodopsin-like GPCR superfamily and integrins (alpha-chain), were found concentrated in the top-half of the list. Many of the candidates were predicted to participate in a variety of essential processes which have no counterpart in mammals, such as molting and synthesis of chitin. Perhaps surprisingly, we also found potential targets that participate in important processes shared across Metazoa. These potential targets are functionally analogous to proteins present in mammals yet they bear no sequence similarity. These include the glycolytic/gluconeogenic enzyme 2,3-bisphosphoglycerate-independent phosphoglycerate mutase (iPGM) characterized previously [21] and the innexin family of gap junction protein [28]. The functions of some of our potential targets are described below in more detail.

**Table 3:** Ranked listing of the top 40 predicted drug targets.

Score	*B. malayi* pub locus	*B. malayi* description	*C. elegans* homolog
275	**Bm1_35120**	PAN domain containing protein	*noah-2*
248	**Bm1_36170**	PAN domain containing protein	*noah-1*
248	Bm1_45135	Conserved hypothetical protein, putative	*pqn-83*
179	**Bm1_35215**	chitin synthase 1, chs-1	*chs-1*
172	Bm1_36850	hypothetical protein	C01B10.11
172	**Bm1_22725**	RNA dependent RNA polymerase family protein	*ego-1*
157	Bm1_15245	RH17657p-related	C25H3.9
157	Bm1_43465	Temporarily assigned gene name protein 40, putative	*nrf-6*
154	Bm1_38120	hypothetical protein	W04G3.8
151	Bm1_35395	Acyltransferase family protein	T14D7.2
143	Bm1_36765	SD01790p-related	Y41E3.1
141	Bm1_25640	hypothetical protein	ZC247.1
140	**Bm1_35480**	hypothetical protein	*mlt-8*
133	Bm1_49915	conserved hypothetical protein	K07A12.7
125	Bm1_45670	WH2 motif family protein	C34E10.11
123	**Bm1_37495**	conserved hypothetical protein	*mlt-9*
119	Bm1_46940	hypothetical protein	C52A11.2
116	Bm1_38110	hypothetical protein	W04G3.2
115	Bm1_32730	LBP/BPI/CETP family, C-terminal domain containing protein	C06G1.1
114	Bm1_42470	hypothetical protein	B0491.5
112	Bm1_55705	Conserved hypothetical protein, putative	B0205.11
110	Bm1_38105	hypothetical protein	W04G3.3
108	Bm1_38425	3′-5′ exonuclease family protein	C10G6.1
107	Bm1_43740	conserved hypothetical protein	T14D7.2
107	Bm1_19285	Innexin family protein	*inx-4*
106	Bm1_51995	LBP/BPI/CETP family, C-terminal domain containing protein	F44A2.3
105	**Bm1_38160**	Fatty acid desaturase family protein	*fat-2*
105	Bm1_02135	ribosomal protein L9 domain containing protein	B0205.11
103	Bm1_03880	hypothetical protein	Y71G12B.13
100	Bm1_35075	Innexin inx-3, putative	*inx-3*
99	Bm1_31660	hypothetical protein	C55C3.5
98	**Bm1_02195**	hypothetical protein	*mlt-8*
98	Bm1_09270	Skp1 related (ubiquitin ligase complex component) protein 18-like	*skr-18*
97	Bm1_50630	hypothetical protein	T19B10.2
96	**Bm1_08695**	*trehalose-6-phosphate phosphatase, putative*	*gob-1*
96	Bm1_39265	GH05862p-related	F42G8.10
91	**Bm1_34455**	amine oxidase, flavin-containing-related *(putative UDP galactopyranomutase)*	H04M03.4
88	Bm1_08915	hypothetical protein *(immunoGlobulin-like Cell adhesion Molecule family)*	*igcm-3*
84	Bm1_16245	symbol-related	ZK809.3
82	Bm1_33575	hypothetical protein	ZK899.2

Previously identified targets are shown with bold identifiers. Manually added annotations are shown in italics. *B. malayi* pub locus and descriptions are from Ghedin *et al.* [11]. *C. elegans* gene names and RNAi phenotypes are from Wormbase.

### Molting

Several potential *B. malayi* targets identified by our bioinformatics approach may mediate molting. Nematode molting, which takes place 4 times from hatching to adulthood, is a highly regulated and complex process involving the synthesis and secretion of a new exoskeleton, followed by the separation and shedding of the old cuticle [29]. Steroid hormones have been implicated in triggering molting in nematodes, as found in arthropods [30], [31]. A recent genome-wide RNAi screen in *C. elegans* has identified 159 genes that are required for molting [32]. These genes may mediate distinct aspects of the process, from intracellular signaling (such as hypodermal-specific transcription factors) to extracellular execution (such as cuticle-digesting proteases). The sequencing of the *B. malayi* genome has revealed that almost all these genes have a *B. malayi* counterpart [11], pointing to phylum-wide conservation in the molting machinery, validating *C. elegans* as a good model for this process. There is wide agreement that molting represents an excellent process for chemotherapeutic intervention, given that it is an ancestral feature of the phylum Nematoda and does not occur in vertebrates [32], [33]. Consistent with this, we recovered more than a dozen *B. malayi* orthologs of proteins necessary for molting in *C. elegans* which could be considered potential drug targets. These include the *B. malayi* orthologs of *C. elegans* NOAH-1 and NOAH-2, which contain zona pellucida (ZP) domains and several plasminogen N-terminal (PAN) modules. These proteins share similarity with *Drosophila melanogaster* NompA, a component of the extracellular matrix [34]. Other high-ranking targets include the orthologs of *C. elegans bli-5* and *mlt-11,* which encode predicted serine-peptidase inhibitors containing multiple Kunitz/Bovine trypsin inhibitor domains. These protease inhibitors may play a role in regulating the activity of hypodermally-expressed subtilisin-like peptidases, such as BLI-4, which could be required for processing cuticular collagens and activation of further collagen processing/degrading enzymes, such as astacin metallopeptidases [35]. Significantly, Kunitz-type serine protease inhibitors have been implicated in molting in the related filarial nematode *Onchocerca volvulus* [36], further supporting the hypothesis that the molecular machinery involved in the molting process is conserved between filarial and rhabditid nematodes.

We also identified *B. malayi* orthologs of *C. elegans mlt-8* and *mlt-9*. *mlt-8* encodes a novel protein that has been proposed to act as an amplifier of endocrine cues during synthesis of the new cuticle, while MLT-9 may be involved in hypodermal signaling [32]. In addition, we identified orthologs of the *C. elegans* Patched signaling family member *ptr-23* and Hedgehog signaling family members *qua-1* and *wrt-4*. These genes have been demonstrated to play a role in molting, even though their functions in the process remain unclear [32], [37]–[39]. In particular, *qua-1*, which has been implicated in hypodermal signaling, encodes a nematode-specific cysteine peptidase capable of autocatalytic activation. *qua-1* is essential for ecdysis and viability: deletion mutants arrest at the first molt (L1 to L2) exhibiting severe morphological abnormalities. *qua-1* orthologs are both well conserved and ubiquitous throughout the phylum Nematoda [39], making QUA-1 a particularly attractive target for the development of specific inhibitors [33].

### Structural Components

*C. elegans* has become one of the preferred models to investigate the assembly and molecular interactions of cell junctions because cell-cell and cell-matrix attachment components are generally well conserved between nematodes and vertebrates (reviewed in [40]). However, a few nematode-specific components do exist, some of which were identified in our screen, including the *B. malayi* homologs of *C.elegans ajm-1* and *pat-12/gei-16.* The *C. elegans* coiled-coil protein AJM-1 localizes to apical junctions and is required for embryonic elongation and maintenance of epithelial integrity [41], [42]. *C. elegans pat-12/gei-16* has been implicated in the formation of Fibrous Organelles (FOs), which are found exclusively in nematodes and mediate attachment between body wall muscle and the cuticle across the hypodermis. FOs are essential for viability, ensure maintenance of body rigidity and allow for locomotion [43]. Phenotypic inspection of *pat-12/gei-16* mutants, together with the molecular characterization of the gene product function, suggest that the protein acts as an adaptor providing linkages between the various structural components of FOs (Benjamin D. Williams and Caroline A. Behm, personal communication; [44], [45]). It is noteworthy that in the human filarial nematode *O. volvulus,* the homolog of *gei-16* encodes the well-characterized OvB20 larval antigen [46], [47]. Immunogold electron microscopy of *O. lienalis* with a OvB20-specific serum revealed localization to discrete foci in the hypodermis and cuticle [47], suggesting that the essential function of *pat-12/gei-16* homologs in formation of FOs is likely to be evolutionarily conserved in filiarial nematodes.

Eight *B. malayi* innexin homologs were identified as potential targets (see Tables S1 and S2). Innexins are invertebrate structural proteins that form intercellular channels, or gap junctions, allowing electrical coupling between adjacent cells (reviewed in [28]). Distantly related connexins in vertebrates perform analogous functions. In *C. elegans*, the innexin family comprises 25 paralogs, showing different spatio-temporal expression patterns [48]. Detailed studies on seven *C. elegans inx* genes have revealed that particular *inx* genes are required for distinct processes including locomotion, egg laying, synchronized contraction of the pharyngeal musculature and inhibition of oocyte maturation [28], [49]. Notably, the innexin genes *unc-7* and *unc-9,* which are required for locomotion, also modulate response to the anthelmintic drug ivermectin [50]–[52].

Chitin is a structural component of the eggshell [53] and pharynx [54] of nematodes and it is absent in mammals. As expected, our analyses revealed the two chitin synthase genes previously proposed as drug targets in *B. malayi* [22], [55] and *O. volvulus* [22]. These genes are orthologs of the two chitin synthase genes present in the *C. elegans* genome that are responsible for chitin deposition in the eggshell (*chs-1*) and pharynx (*chs-2*) and essential for development [54]. Functional conservation of nematode chitin synthases is highly likely since the *B. malayi chs*-1 transcript is predominantly found in the oocytes and early embryos [55]. Orthologs of two other *C. elegans* genes (H02I12.1 and W03F11.1) encoding proteins containing putative chitin binding domains, were also identified. Interestingly, RNAi against H02I12.1, which contains a peritrophin A chitin-binding module, compromises the egg osmotic integrity during early embryogenesis [56], suggesting that this gene plays a role in eggshell chitin deposition. Thus, aspects of chitin metabolism are clearly essential in nematodes and involve a number of components worthy of further evaluation as drug targets.

The sugar galactofuranose (Gal*_f_*) is an important component of cell surface glycoconjugates of several prokaryotic and eukaryotic pathogens and has been shown to be essential for viability and virulence [57]–[59]. From the *B. malayi* genome, we annotated two putative orthologs of UDP-galactopyranose mutase (GLF), the enzyme that is required for biosynthesis of Gal*_f_*. Both the sugar and the enzyme are absent from mammals making GLF an attractive drug target [57].

### Central Metabolism

In nematodes, the glucose disaccharide trehalose is proposed to serve as an energy reserve and a protectant against various environmental stresses such as heat, cold and freezing, oxidative and osmotic stress, anoxia, even dessication and anhydrobiosis [60], [61]. It is an abundant storage sugar in the filarial nematodes *Brugia pahangi* and *Acanthocheilonema viteae* [62] and is also found in bacteria, fungi and insects but not in mammals. We identified trehalose-6-phosphate phosphatase as an ortholog of the essential *C. elegans* gene *gob-1* (gut obstructed). Removal of this gene activity in *C. elegans* gives rise to larval lethality, partly due to intestinal blockage and subsequent starvation [63]. This *gob-1* lethality is completely suppressed when the upstream trehalose-6-phosphate synthase genes are deleted, indicating that the lethality is due to toxic build-up of the intermediate trehalose-6-phosphate [63].

Mammals take up various unsaturated fatty acids from food as essential nutrients whereas C. *elegans* has fatty acid desaturases that catalyze the production of polyunsaturated fatty acids [64]. Among the highly ranked targets was the *B. malayi* ortholog of the essential *C. elegans fat-2* gene encoding a Δ-12 fatty acid desaturase that converts oleic acid (18:1) to linoleic acid (18:2) implying that *B. malayi* also synthesizes polyunsaturated fatty acids rather than acquiring them from the host environment.

The glycolytic/gluconeogenic pathway is present in most cellular organisms, however, the enzymes in the pathway may not be conserved. We identified a 2,3-bisphosphoglycerate-independent phosphoglycerate mutase (iPGM) as such an example. This enzyme has a distinct sequence and structure from the 2,3-bisphosphoglycerate-dependent phosphoglycerate mutase (dPGM) found in mammals. Both enzymes are responsible for the interconversion of 2-phosphoglycerate and 3-phosphoglycerate, however different catalytic mechanisms are involved. The biochemical activities of both *B. malayi* and *C. elegans* iPGM enzymes have been demonstrated as well as the essentiality of the gene for nematode development. Down regulation of *C. elegans* iPGM using RNAi, results in embryonic and larval lethality [21].

### Nucleic Acid Metabolism

Other potentially interesting targets revealed by our analysis include orthologs of *C. elegans* transcription factors *lin-14*, *die-1* and *pry-1* known to be involved in key developmental and morphogenetic processes. *C. elegans lin-14* is a nematode-specific transcription factor required for larval stage-specific gene expression [65]. Mutations in *lin-14* cause cell lineage defects in several cell types. The *C. elegans* gene *die-1* belongs to the zinc finger family of transcription factors. Loss of *die-1* affects epithelial cell rearrangements during embryonic epidermal morphogenesis, leading ultimately to embryonic arrest [66]. We also recovered the *B. malayi* homolog of *C. elegans pry-1* [67] encoding a protein with limited homology to vertebrate Axins, which act as scaffold proteins in the Wnt/beta-catenin signaling pathway [68]. Despite its sequence divergence, PRY-1, like Axin, serves as a negative regulator in the Wnt signaling pathway in *C. elegans* and can functionally complement for the *Danio rerio* (zebrafish) *axin1* knockout *masterblind* [69]. This example illustrates how specific components of signaling pathways, which are conserved between vertebrates and nematodes but have diverged at the primary sequence level, may differ sufficiently to allow for the development of nematode-specific inhibitors.

We also identified genes involved in RNA processing. *Trans*-splicing, which involves the addition of a short leader sequence to the 5′-end of mRNA, is an essential step in the maturation of most mRNAs in nematodes and several other invertebrates and protozoa (reviewed in [70]). Our analysis identified the *B. malayi* orthologs of two known components (SL30p and SL95p) required for *in vitro* RNA *trans*-splicing in embryonic lysates from the human nematode *Ascaris lumbricoides* [71]. Recently, orthologs of these two genes in *C. elegans* (*sut-1* and *sna-2* respectively) have also been implicated in RNA *trans*-splicing [72]. Additionally, we identified an ortholog of *C. elegans ego-1,* which belongs to a family of RNA-directed RNA polymerases. *ego-1* is essential for viability and fertility and in particular plays a crucial role in germline development, where it promotes cell proliferation, meiosis, and gametogenesis. It is thought that EGO-1 influences all these distinct processes by inducing and reinforcing germline RNAi of specific genes [73]–[75]. While many components of the RNAi pathway appear to be missing from the *B. malayi* genome, most notably the spreading machinery [11], presence of *ego-1* suggests conservation of the role of this class of RNA-directed RNA polymerases in germline silencing across Nematoda.

In addition to drug target discovery, our method highlights proteins participating in biological processes that are necessarily conserved across parasitic and free-living worms; in the case of *B. malayi* and the sequenced Caenorhabditids these processes span an evolutionary distance of 350 million years since their last common ancestor [11]. This substantially extends our confidence in identifying nematode-centric processes over those conserved only between the Caenorhabditid genomes. Significantly, 50% of the targets were annotated as hypothetical proteins. These may participate in completely novel nematode processes and are worthy of further study.

The recently completed draft genomic sequence of *B. malayi* has enabled us to predict potentially essential genes and apply a method for rational drug target discovery. In contrast to empirical methods, the bioinformatics approach described herein yields a larger pool of candidates and is not biased, thereby providing a wider range of potential targets. Given the threat of emerging drug resistance resulting from continued reliance on a limited repertoire of available drugs, a wider array of choices for drug targets will be invaluable. The method is also tunable and quickly provides a manageable set of targets for closer analysis. By adjusting the parameters of the comparative sequence analysis, the initial target pool size can be increased or decreased by an order of magnitude. Varying the weights for the factors used in the prioritization scheme can tailor the ranking to the needs of the end-user.

The basic subtractive filtering methodology is applicable to a wide variety of sequenced pathogens, ranging from microbial species to the metazoan parasite analyzed here. Although it is currently limited by the availability of complete genome sequence and functional genomics data, the rapid pace of technological advancements in these areas will soon overcome those limitations, and we expect this methodology to gain widespread applicability.

## Materials and Methods

### Data sources

DNA sequences and protein translations for the *B. malayi* genome are as described [11]. The sequence set used in this study differs slightly from the final released genome, though efforts were made to maintain synchrony with the final release. Stage specific ESTs and tentative consensus sequences for *B. malayi* were obtained from the TIGR gene indices project (now housed at Dana Farber Cancer Institute, http://compbio.dfci.harvard.edu/tgi). Complete DNA coding sequence, protein sequence, and RNAi phenotype data from release 150 of the *C. elegans* genome was obtained from Wormbase (http://www.wormbase.org). Human genome protein sequences were obtained from Ensembl release 41 (http://www.ensembl.org) corresponding to the NCBI build 36 of the human genome.

### Ortholog/homolog assignments

Ortholog assignments were based on WashU BLASTP all-vs-all analysis, Jaccard clustering, and bidirectional best hit clustering, as described [11]. These assignments were supplemented with one-way best hits using NCBI BLASTP [76] with an e-value cutoff of 1×10^−20^, BLOSUM 62 as the scoring matrix and *B. malayi* sequences as the query. Similarity between *B. malayi* sequences and human sequences was established using one-way best hits with NCBI BLASTP with an e-value cutoff of 1×10^−13^ and *B. malayi* sequences as the query. E-value cutoffs were empirically adjusted to maintain a reasonable target pool size for subsequent literature scanning and retain known potential drug targets, chitin synthase 1 and 2, within the target pool.

### GO terms

Gene ontology (GO) term assignments were obtained as described [11] where essentially the following procedure was used. Interpro domain assignments were first applied to *B. malayi* proteins using InterproScan. GO terms attached to the InterPro domains were transferred to the *B. malayi* gene products using Interpro2GO (http://www.ebi.ac.uk/interpro). A custom GO slim subset of ontology terms generated by TIGR for the *B. malayi* sequencing project was used to provide a broad overview of the ontology content. Over-representation of GO terms was analyzed using the program Ontologizer [77] with a p-value cutoff of 0.05, Bonferroni correction, and term-for-term methodology.

### Protein properties

Average hydropathy scores (GRAVY) were calculated as the average of the individual hydropathy scores for each amino acid using the data of Kyte and Doolittle [78]. EC numbers were mapped to *B. malayi* proteins using pre-compiled mapping of EC numbers to GO terms, revision 1.54, available from http://www.geneontology.org/external2go/ec2go.

### RNAi phenotype binning

*C. elegans* RNAi phenotypes associated with orthologs of the *B. malayi* candidate drug target sequences were binned into 9 categories with corresponding weights as shown:

**Table:** 

**Bin**	**Weight**
larval/adult lethality/arrest	100
embryonic lethality	90
morphology defect	80
growth defect	70
movement defect	60
vulval/egg laying defect	50
other/unclassified	10
wild-type	0

To establish a background distribution, all *C. elegans* RNAi phenotypes were binned into the same categories. Background frequencies were used to estimate expected frequencies for a sample size equal to the size of the RNAi phenotype set associated with the orthologs of the *B. malayi*

## Supporting Information

Supplementary Table S1 Ranked list of candidate targets. Previously identified targets are shown with bold identifiers. Manually added annotations are shown in italics. *B. malayi* pub locus and descriptions are from Ghedin *et al*. [11]. *C. elegans* gene names and RNAi phenotypes are from Wormbase.
(0.30 MB PDF)

Supplementary Table S2 Frequency of Interpro domains in the target sequences.
(0.09 MB PDF)

Supplementary Table S3 EC numbers mapped to targets using ec2go.
(0.05 MB PDF)

Supplementary Table S4 GO terms associated with target pool sequences. The GO terms are a subset of the GO hierarchy (GO slim). All children of the GO slim nodes are mapped up to the nearest parent in the slim hierarchy. Counts total the occurrences of the exact GO term listed and all its children.
(0.07 MB PDF)

Supplementary Table S5 RNAi phenotype components of each binning category.
(0.12 MB PDF)

Supplementary Data Set S1 Data set for target prioritization. Data values used in assigning scores for prioritization of targets. Maximum bit scores for alignments of putative *B. malayi*, *C. elegans* and Human orthologs were obtained from BLASTP results (see materials and methods). Bitscores of 0.0 are recorded when no similarity was identified with an E-value below the threshold used in the BLAST comparison. The total number of RNAi experiments reported for each target gene are based on wormbase release 150. Pheno Bins record the number of instances that a phenotype was reported in these experiments that belongs to each of 9 phenotype bins (see text). In this table, the “other/unclassified” bin was split into “other” and “unclassified” bins. Stage expression count refers to the number of distinct life cycle stages (L2, L3, L4, adult and microfilariae) having EST evidence for a particular target gene. L2, L3, L4, adult and microfilariae record the number of ESTs for that stage. Total Score was calculated as described in Table 2. Known targets are indicated in bold. Na indicates “no value”.
(0.13 MB PDF)

## References

[1] Galperin MY, Koonin EV (1999). Searching for drug targets in microbial genomes.. Curr Opin Biotechnol.

[2] Rosamond J, Allsop A (2000). Harnessing the power of the genome in the search for new antibiotics.. Science.

[3] Sakharkar KR, Sakharkar MK, Chow VT (2004). A novel genomics approach for the identification of drug targets in pathogens, with special reference to *Pseudomonas aeruginosa*.. In Silico Biol.

[4] Foster JM, Zhang Y, Kumar S, Carlow CK (2005). Mining nematode genome data for novel drug targets.. Trends Parasitol.

[5] McCarter JP (2004). Genomic filtering: an approach to discovering novel antiparasitics.. Trends Parasitol.

[6] Behm CA, Bendig MM, McCarter JP, Sluder AE (2005). RNAi-based discovery and validation of new drug targets in filarial nematodes.. Trends Parasitol.

[7] WHO (2004). The world health report 2004 - changing history..

[8] Hoerauf A (2006). New strategies to combat filariasis.. Expert Rev Anti Infect Ther.

[9] Osei-Atweneboana MY, Eng JK, Boakye DA, Gyapong JO, Prichard RK (2007). Prevalence and intensity of *Onchocerca volvulus* infection and efficacy of ivermectin in endemic communities in Ghana: a two-phase epidemiological study.. Lancet.

[10] Schwab AE, Boakye DA, Kyelem D, Prichard RK (2005). Detection of benzimidazole resistance-associated mutations in the filarial nematode *Wuchereria bancrofti* and evidence for selection by albendazole and ivermectin combination treatment.. Am J Trop Med Hyg.

[11] Ghedin E, Wang S, Spiro D, Caler E, Zhao Q (2007). Draft genome of the filarial nematode parasite *Brugia malayi*.. Science.

[12] Bieri T, Blasiar D, Ozersky P, Antoshechkin I, Bastiani C (2007). WormBase: new content and better access.. Nucleic Acids Res.

[13] Burglin TR, Lobos E, Blaxter ML (1998). Caenorhabditis elegans as a model for parasitic nematodes.. Int J Parasitol.

[14] Hashmi S, Tawe W, Lustigman S (2001). *Caenorhabditis elegans* and the study of gene function in parasites.. Trends Parasitol.

[15] Brooks DR, Isaac RE (2002). Functional genomics of parasitic worms: the dawn of a new era.. Parasitol Int.

[16] Aboobaker AA, Blaxter ML (2003). Use of RNA interference to investigate gene function in the human filarial nematode parasite *Brugia malayi*.. Mol Biochem Parasitol.

[17] Lustigman S, Zhang J, Liu J, Oksov Y, Hashmi S (2004). RNA interference targeting cathepsin L and Z-like cysteine proteases of *Onchocerca volvulus* confirmed their essential function during L3 molting.. Mol Biochem Parasitol.

[18] Heider U, Blaxter M, Hoerauf A, Pfarr KM (2006). Differential display of genes expressed in the filarial nematode *Litomosoides sigmodontis* reveals a putative phosphate permease up-regulated after depletion of *Wolbachia* endobacteria.. Int J Med Microbiol.

[19] Pfarr K, Heider U, Hoerauf A (2006). RNAi mediated silencing of actin expression in adult *Litomosoides sigmodontis* is specific, persistent and results in a phenotype.. Int J Parasitol.

[20] Hasan S, Daugelat S, Rao PS, Schreiber M (2006). Prioritizing genomic drug targets in pathogens: application to *Mycobacterium tuberculosis*.. PLoS Comput Biol.

[21] Zhang Y, Foster JM, Kumar S, Fougere M, Carlow CK (2004). Cofactor-independent phosphoglycerate mutase has an essential role in *Caenorhabditis elegans* and is conserved in parasitic nematodes.. J Biol Chem.

[22] Foster JM, Zhang Y, Kumar S, Carlow CK (2005). Parasitic nematodes have two distinct chitin synthases.. Mol Biochem Parasitol.

[23] Echeverri CJ, Beachy PA, Baum B, Boutros M, Buchholz F (2006). Minimizing the risk of reporting false positives in large-scale RNAi screens.. Nat Methods.

[24] Blaxter M, Daub J, Guiliano D, Parkinson J, Whitton C (2002). The *Brugia malayi* genome project: expressed sequence tags and gene discovery.. Trans R Soc Trop Med Hyg.

[25] Hopkins AL, Groom CR (2002). The druggable genome.. Nat Rev Drug Discov.

[26] Robertson JG (2005). Mechanistic basis of enzyme-targeted drugs.. Biochemistry.

[27] Luan CH, Qiu S, Finley JB, Carson M, Gray RJ (2004). High-throughput expression of *C. elegans* proteins.. Genome Res.

[28] Phelan P (2005). Innexins: members of an evolutionarily conserved family of gap-junction proteins.. Biochim Biophys Acta.

[29] Singh RN, Sulston JE (1978). Some observations on molting in *C. elegans*.. Nematologica.

[30] Kuervers LM, Jones CL, O'Neil NJ, Baillie DL (2003). The sterol modifying enzyme LET-767 is essential for growth, reproduction and development in *Caenorhabditis elegans*.. Mol Genet Genomics.

[31] Kostrouchova M, Krause M, Kostrouch Z, Rall JE (2001). Nuclear hormone receptor CHR3 is a critical regulator of all four larval molts of the nematode *Caenorhabditis elegans*.. Proc Natl Acad Sci U S A.

[32] Frand AR, Russel S, Ruvkun G (2005). Functional genomic analysis of *C. elegans* molting.. PLoS Biol.

[33] Craig H, Isaac RE, Brooks DR (2007). Unravelling the moulting degradome: new opportunities for chemotherapy?. Trends Parasitol.

[34] Chung YD, Zhu J, Han Y, Kernan MJ (2001). nompA encodes a PNS-specific, ZP domain protein required to connect mechanosensory dendrites to sensory structures.. Neuron.

[35] Page AP, McCormack G, Birnie AJ (2006). Biosynthesis and enzymology of the *Caenorhabditis elegans* cuticle: identification and characterization of a novel serine protease inhibitor.. Int J Parasitol.

[36] Ford L, Guiliano DB, Oksov Y, Debnath AK, Liu J (2005). Characterization of a novel filarial serine protease inhibitor, Ov-SPI-1, from *Onchocerca volvulus*, with potential multifunctional roles during development of the parasite.. J Biol Chem.

[37] Zugasti O, Rajan J, Kuwabara PE (2005). The function and expansion of the Patched- and Hedgehog-related homologs in *C. elegans*.. Genome Res.

[38] Hao L, Johnsen R, Lauter G, Baillie D, Burglin TR (2006). Comprehensive analysis of gene expression patterns of *hedgehog*-related genes.. BMC Genomics.

[39] Hao L, Mukherjee K, Liegeois S, Baillie D, Labouesse M (2006). The *hedgehog*-related gene *qua-1* is required for molting in *Caenorhabditis elegans*.. Dev Dyn.

[40] Labouesse M (2006). Epithelial junctions and attachments.. The *C. elegans* Research Community, editor. WormBook.

[41] Koppen M, Simske JS, Sims PA, Firestein BL, Hall DH (2001). Cooperative regulation of AJM-1 controls junctional integrity in *Caenorhabditis elegans* epithelia.. Nat Cell Biol.

[42] McMahon L, Legouis R, Vonesch JL, Labouesse M (2001). Assembly of *C. elegans* apical junctions involves positioning and compaction by LET-413 and protein aggregation by the MAGUK protein DLG-1.. J Cell Sci.

[43] Francis R, Waterston RH (1991). Muscle cell attachment in *Caenorhabditis elegans*.. J Cell Biol.

[44] Williams BD, Waterston RH (1994). Genes critical for muscle development and function in *Caenorhabditis elegans* identified through lethal mutations.. J Cell Biol.

[45] Tsuboi D, Qadota H, Kasuya K, Amano M, Kaibuchi K (2002). Isolation of the interacting molecules with GEX-3 by a novel functional screening.. Biochem Biophys Res Commun.

[46] Abdel-Wahab N, Kuo YM, Wu Y, Tuan RS, Bianco AE (1996). OvB20, an *Onchocerca volvulus*-cloned antigen selected by differential immunoscreening with vaccination serum in a cattle model of onchocerciasis.. Mol Biochem Parasitol.

[47] Taylor MJ, Abdel-Wahab N, Wu Y, Jenkins RE, Bianco AE (1995). *Onchocerca volvulus* larval antigen, OvB20, induces partial protection in a rodent model of onchocerciasis.. Infect Immun.

[48] Starich T, Sheehan M, Jadrich J, Shaw J (2001). Innexins in *C. elegans*.. Cell Commun Adhes.

[49] Whitten SJ, Miller MA (2007). The role of gap junctions in *Caenorhabditis elegans* oocyte maturation and fertilization.. Dev Biol.

[50] Barnes TM, Hekimi S (1997). The *Caenorhabditis elegans* avermectin resistance and anesthetic response gene *unc-9* encodes a member of a protein family implicated in electrical coupling of excitable cells.. J Neurochem.

[51] Dent JA, Smith MM, Vassilatis DK, Avery L (2000). The genetics of ivermectin resistance in *Caenorhabditis elegans*.. Proc Natl Acad Sci U S A.

[52] Starich TA, Lee RY, Panzarella C, Avery L, Shaw JE (1996). *eat-5* and *unc-7* represent a multigene family in *Caenorhabditis elegans* involved in cell-cell coupling.. J Cell Biol.

[53] Bird AF, Bird J (1991). The Structure of Nematodes..

[54] Zhang Y, Foster JM, Nelson LS, Ma D, Carlow CK (2005). The chitin synthase genes *chs-1* and *chs-2* are essential for *C. elegans* development and responsible for chitin deposition in the eggshell and pharynx, respectively.. Dev Biol.

[55] Harris MT, Lai K, Arnold K, Martinez HF, Specht CA (2000). Chitin synthase in the filarial parasite, *Brugia malayi.*. Mol Biochem Parasitol.

[56] Sonnichsen B, Koski LB, Walsh A, Marschall P, Neumann B (2005). Full-genome RNAi profiling of early embryogenesis in *Caenorhabditis elegans*.. Nature.

[57] Beverley SM, Owens KL, Showalter M, Griffith CL, Doering TL (2005). Eukaryotic UDP-galactopyranose mutase (GLF gene) in microbial and metazoal pathogens.. Eukaryot Cell.

[58] Kleczka B, Lamerz AC, van Zandbergen G, Wenzel A, Gerardy-Schahn R (2007). Targeted gene deletion of *Leishmania major* UDP-galactopyranose mutase leads to attenuated virulence.. J Biol Chem.

[59] Pan F, Jackson M, Ma Y, McNeil M (2001). Cell wall core galactofuran synthesis is essential for growth of mycobacteria.. J Bacteriol.

[60] Behm CA (1997). The role of trehalose in the physiology of nematodes.. Int J Parasitol.

[61] Elbein AD, Pan YT, Pastuszak I, Carroll D (2003). New insights on trehalose: a multifunctional molecule.. Glycobiology.

[62] Powell JW, Stables JN, Watt RA (1986). An investigation of the glucose metabolism of *Brugia pahangi* and *Dipetalonema viteae* by nuclear magnetic resonance spectroscopy.. Mol Biochem Parasitol.

[63] Kormish JD, McGhee JD (2005). The *C. elegans* lethal gut-obstructed *gob-1* gene is trehalose-6-phosphate phosphatase.. Dev Biol.

[64] Watts JL, Browse J (2002). Genetic dissection of polyunsaturated fatty acid synthesis in *Caenorhabditis elegans*.. Proc Natl Acad Sci U S A.

[65] Hristova M, Birse D, Hong Y, Ambros V (2005). The *Caenorhabditis elegans* heterochronic regulator LIN-14 is a novel transcription factor that controls the developmental timing of transcription from the insulin/insulin-like growth factor gene *ins-33* by direct DNA binding.. Mol Cell Biol.

[66] Heid PJ, Raich WB, Smith R, Mohler WA, Simokat K (2001). The zinc finger protein DIE-1 is required for late events during epithelial cell rearrangement in *C. elegans*.. Dev Biol.

[67] Maloof JN, Whangbo J, Harris JM, Jongeward GD, Kenyon C (1999). A Wnt signaling pathway controls *hox* gene expression and neuroblast migration in *C. elegans*.. Development.

[68] Mao J, Wang J, Liu B, Pan W, Farr GH (2001). Low-density lipoprotein receptor-related protein-5 binds to Axin and regulates the canonical Wnt signaling pathway.. Mol Cell.

[69] Korswagen HC, Coudreuse DY, Betist MC, van de Water S, Zivkovic D (2002). The Axin-like protein PRY-1 is a negative regulator of a canonical Wnt pathway in *C. elegans*.. Genes Dev.

[70] Blumenthal T (1995). Trans-splicing and polycistronic transcription in *Caenorhabditis elegans*.. Trends Genet.

[71] Denker JA, Zuckerman DM, Maroney PA, Nilsen TW (2002). New components of the spliced leader RNP required for nematode trans-splicing.. Nature.

[72] MacMorris M, Kumar M, Lasda E, Larsen A, Kraemer B (2007). A novel family of *C. elegans* snRNPs contains proteins associated with trans-splicing.. RNA.

[73] Maine EM, Hauth J, Ratliff T, Vought VE, She X (2005). EGO-1, a putative RNA-dependent RNA polymerase, is required for heterochromatin assembly on unpaired dna during *C. elegans* meiosis.. Curr Biol.

[74] Qiao L, Lissemore JL, Shu P, Smardon A, Gelber MB (1995). Enhancers of *glp-1*, a gene required for cell-signaling in *Caenorhabditis elegans*, define a set of genes required for germline development.. Genetics.

[75] Vought VE, Ohmachi M, Lee MH, Maine EM (2005). EGO-1, a putative RNA-directed RNA polymerase, promotes germline proliferation in parallel with GLP-1/notch signaling and regulates the spatial organization of nuclear pore complexes and germline P granules in *Caenorhabditis elegans*.. Genetics.

[76] Altschul SF, Gish W, Miller W, Myers EW, Lipman DJ (1990). Basic local alignment search tool.. J Mol Biol.

[77] Grossmann S, Bauer P, Robinson PN, Vingron MM (2006). An Improved Statistic for Detecting Over-Represented Gene Ontology Annotations in Gene Sets.. Research in Computational Molecular Biology.

[78] Kyte J, Doolittle RF (1982). A simple method for displaying the hydropathic character of a protein.. J Mol Biol.

