# Supplementary material for: Mining Predicted Essential Genes of Brugia malayi for Nematode Drug Targets
Source: PLoS One. 2007 Nov 14;2(11):e1189. doi: 10.1371/journal.pone.0001189 (PMC2063515; doi:10.1371/journal.pone.0001189)
Supplement: Supplementary Table S1 — Ranked list of candidate targets. Previously identified targets are shown with bold identifiers. Manually added annotations are shown in italics. B. malayi pub locus and descriptions are from Ghedin et al. [11]. C. elegans gene names and RNAi phenotypes are from Wormbase. (0.30 MB PDF) [file pone.0001189.s001.pdf]

Supplementary Table S1. Ranked list of candidate targets.

Score	TIGR *B.malayi* pub locus	*B. malayi* gene description	*C. elegans* ortholog	*C. elegans* ortholog RNAi phenotypes
275	**Bm1_35120**	PAN domain containing protein	*noah-2*	Lva, Sck, Emb, Lvl, Unc, Ste, WT
248	**Bm1_36170**	PAN domain containing protein	*noah-1*	Bmd, Lva, Muv, Emb, Lvl, Unc, WT
248	Bm1_45135	Conserved hypothetical protein, putative	*pqn-83*	Let, Unc, Ste
179	**Bm1_35215**	chitin synthase 1, chs-1	*chs-1*	Emb, Ste, Sterility/Impaired Fertility in F0, Gro, WT
172	Bm1_36850	hypothetical protein	C01B10.11	Dpy, Unc
172	**Bm1_22725**	RNA dependent RNA polymerase family protein	*ego-1*	Cyk, Emb, WT, Gro
157	Bm1_15245	RH17657p-related	C25H3.9	General Pace of Development abnormal, Emb
157	Bm1_43465	Temporarily assigned gene name protein 40, putative	*nrf-6*	Dpy, Bmd, Slu, Emb, WT
154	Bm1_38120	hypothetical protein	W04G3.8	Prz, Lvl, Unc, WT, Larval Lethal-Early (L1/L2), Gro
151	Bm1_35395	Acyltransferase family protein	T14D7.2	Egl, Pvl, Unc, WT
143	Bm1_36765	SD01790p-related	Y41E3.1	Sck, Ste, WT
141	Bm1_25640	hypothetical protein	ZC247.1	Sck, Ste, Sma, WT, Stp, Gro
140	**Bm1_35480**	hypothetical protein	*mlt-8*	Bmd, Prz, Lva, Larval Arrest-Early (L1/L2), Lvl, Unc, WT
133	Bm1_49915	conserved hypothetical protein	K07A12.7	Emb, WT
125	Bm1_45670	WH2 motif family protein	C34E10.11	Emb, WT
123	**Bm1_37495**	conserved hypothetical protein	*mlt-9*	Prz, Larval Lethal-Late (L3/L4), Unclassified, Mlt, Unc, Lva, Sck, Gro, WT
119	Bm1_46940	hypothetical protein	C52A11.2	Dpy, Lva, Egl, Emb, Unc, Rup, WT
116	Bm1_38110	hypothetical protein	W04G3.2	Bmd, Lva, Lvl, Unc, WT
115	Bm1_32730	LBP / BPI / CETP family, C-terminal domain containing protein	C06G1.1	Lon, Unc, WT
114	Bm1_42470	hypothetical protein (contains similarity to Aedes aegypti Putative mitochondrial NADH-ubiquinone oxidoreductase (MitochondrialsNADH:ubiquinone oxidoreductase B14.7 subunit, putative).; TR:Q1HQR1)	B0491.5	Bmd, Lva, Emb, Unclassified, Lvl, WT

112	Bm1_55705	Conserved hypothetical protein, putative	B0205.11	Emb, WT
110	Bm1_38105	hypothetical protein	W04G3.3	Unc, WT
108	Bm1_38425	3'-5' exonuclease family protein	C10G6.1	Mlt, Unc, Rup, WT
107	Bm1_43740	conserved hypothetical protein	T14D7.2	Egl, Pvl, Unc, WT
107	Bm1_19285	Innexin family protein	*inx-4*	Unclassified, WT
106	Bm1_51995	LBP / BPI / CETP family, C-terminal domain containing protein	F44A2.3	Spindle Elongation/Integrity abnormal, Emb, WT
105	**Bm1_38160**	Fatty acid desaturase family protein	*fat-2*	Emb, Unclassified, WT
105	Bm1_02135	ribosomal protein L9 domain containing protein	B0205.11	Emb, WT
103	Bm1_03880	hypothetical protein	Y71G12B.13	Emb, WT
100	Bm1_35075	Innexin inx-3, putative	*inx-3*	Lva, Unc, Gro, WT
99	Bm1_31660	hypothetical protein	C55C3.5	Osmotic Integrity defective, Ooc, Emb
98	**Bm1_02195**	hypothetical protein	*mlt-8*	Bmd, Prz, Lva, Larval Arrest-Early (L1/L2), Lvl, Unc, WT
98	Bm1_09270	Skp1 related (ubiquitin ligase complex component) protein 18-like	*skr-18*	Pvl, Unc, Rup, Stp, Gro, WT
97	Bm1_50630	hypothetical protein	T19B10.2	Prz, Rup, Unc, WT, Gro
96	**Bm1_08695**	trehalose-6-phosphate synthase-related	*gob-1*	Slu, Larval Arrest-Late (L3/L4), Gro, WT
96	Bm1_39265	GH05862p-related	F42G8.10	Lva, Emb, Unclassified, WT
91	**Bm1_34455**	amine oxidase, flavin-containing-related (putative UDP galactopyranomutase)	H04M03.4	Prz, Dpy, Lvl, Unclassified, Sma, Unc, WT
88	Bm1_08915	hypothetical protein (immunoGlobulin-like Cell adhesion Molecule family)	*igcm-3*	Unclassified, WT
84	Bm1_16245	symbol-related	ZK809.3	Lva, Emb, Unclassified, WT, Gro
82	Bm1_33575	hypothetical protein	ZK899.2	Lva, Emb, Lvl, WT, Larval Lethal-Early (L1/L2), Gro
82	Bm1_39790	conserved hypothetical protein	W01C8.5	Unclassified, Gro, WT
81	Bm1_33440	Innexin family protein	*inx-13*	Unc, Rup, Stp, Gro, WT
81	Bm1_41495	Gex interacting protein protein 4, isoform c-related	*gei-4*	Let, Sck, Emb, Pvl, Ste, WT
79	Bm1_55030	hypothetical protein	Y67H2A.5	Sck, Emb, Ste, WT
79	Bm1_07680	EB module family protein	F56B3.2	Prz, Lvl, Unclassified, Unc, WT, Gro
77	Bm1_30085	Apoptosis regulator proteins, Bcl-2 family protein	*ced-9*	Integrity of Membranous Organelles defective, Emb, WT
77	Bm1_56645	Hypothetical 19.4 kDa protein T09A5.5 in chromosome III, putative	T09A5.5	Lva, Clr, Emb, Unclassified, WT
75	Bm1_49050	hypothetical protein	ZC328.1	Prz, Dpy, Prl, Lvl, Unc, WT, Gro

75	Bm1_49335	RUN domain containing protein	*unc-14*	Unc, WT
74	Bm1_05470	hypothetical protein	F37C4.4	Emb, WT, Stp
74	Bm1_53115	hypothetical protein	C41G7.3	Him, General Pace of Development abnormal, WT
74	Bm1_41565	hAT family dimerisation domain containing protein	F42H10.5	Emb, WT
73	Bm1_56145	conserved hypothetical protein	*mdt-15*	Clr, Emb, Unc, Ste, Lva, Sck, Adl, WT, Developmental Delay, Gro
72	**Bm1_51960**	conserved hypothetical protein (putative UDP galactopyranomutase)	H04M03.4	Prz, Dpy, Lvl, Unclassified, Sma, Unc, WT
72	Bm1_44220	conserved hypothetical protein	C25H3.6	Bmd, Lva, Emb, Unc, WT
71	Bm1_05960	Patched family protein	*ptr-23*	Unc, WT
71	Bm1_24555	hypothetical protein	F28F8.5	Slu, Emb, Pvl, Unc, Rup, Gro, Stp, WT
71	Bm1_16555	hypothetical protein	*atp-5*	Lva, General Pace of Development abnormal, Emb, Ste, Gro
70	Bm1_35415	50S ribosomal protein L20.-related	Y48C3A.10	Emb, Unclassified, WT
69	Bm1_52630	Lipase family protein	T08B1.4	Aldicarb resistant, WT
68	Bm1_21610	PDZ domain containing protein	T21G5.4	Lva, Pvl, WT, Stp
68	Bm1_45560	hypothetical protein	C09F5.1	Pvl, Ste, WT
67	Bm1_14680	PDZ domain containing protein	T21G5.4	Lva, Pvl, WT, Stp
67	Bm1_14330	Mitochondrial ATP synthase coupling factor 6 family protein	*atp-4*	General Pace of Development abnormal, Emb, Lvl, Ste, Adl, Gro
67	Bm1_47700	Mitochondrial import inner membrane translocase subunit Tim17 family protein	F15D3.7	Emb, Pvl, Severe Pleiotropic Defects, Ste
67	Bm1_07635	GM16138p-related	F49C12.12	Osmotic Integrity defective, Emb, Sterile F0/Fertility Problems, Lvl
67	Bm1_53755	NADH-ubiquinone oxidoreductase subunit B14.5b.-related	Y71H2AM.4	Lva, Emb, WT, Gro
67	Bm1_45175	conserved hypothetical protein	ZC262.8	Emb, WT
66	Bm1_37530	conserved hypothetical protein	*lin-14*	Egl, Pvl, Unc, Sma, Sck, Sterile F1, Stp, WT
65	Bm1_33125	conserved hypothetical protein	ZK1193.5	Lva, Clr, Lvl, Larval Arrest-Late (L3/L4), Gro
65	Bm1_21620	Profilin family protein	*pfn-1*	Cyk, Emb, Unc, Stp
65	Bm1_18695	Nematode cuticle collagen N-terminal domain containing protein	*dpy-3*	Dpy, Unc
64	Bm1_38065	Clc-like	Y38F2AL.1	Dpy, Egl, Unc, WT
64	Bm1_25750	Lipase family protein	F58G1.5	Him, WT
64	Bm1_51095	Innexin inx-10, putative	*inx-10*	Aldicarb resistant, WT

64	Bm1_51050	hypothetical protein	*ril-1*	Lva, General Pace of Development abnormal, Emb, Gro
64	Bm1_44775	hypothetical protein	C23G10.8	Emb, Pvl, Ste, WT, Gro
63	Bm1_32310	Innexin family protein	*inx-3*	Lva, Unc, Gro, WT
63	Bm1_13015	Nematode cuticle collagen N-terminal domain containing protein	*dpy-5*	Dpy, Rup
63	Bm1_44070	Zinc finger, C2H2 type family protein	*die-1*	Bmd, Muv, Emb, Lvl, WT, Gro
63	Bm1_46095	hypothetical protein	F57B10.14	Osmotic Integrity defective, Emb, WT
63	Bm1_08035	Hypothetical 36.0 kDa protein C45G9.5 in chromosome III.-related	C45G9.5	Lva, Integrity of Membranous Organelles defective, Emb, Larval Arrest-Early (L1/L2), Gro
62	Bm1_56805	50S ribosomal protein L10.-related	K01C8.6	Lva, Emb, Larval Arrest-Early (L1/L2), Unclassified, Sma, WT, Gro
62	Bm1_48395	hypothetical protein	Y87G2A.1	Unclassified, Pvl, Rup, Unc, Stp, Gro, WT
62	Bm1_53205	hypothetical protein	T19A5.3	Lvl, WT
61	Bm1_41590	Mitochondrial import inner membrane translocase subunit Tim17 family protein	F15D3.7	Emb, Pvl, Severe Pleiotropic Defects, Ste
61	Bm1_21255	Chitin binding Peritrophin-A domain containing protein	H02I12.1	Osmotic Integrity defective, Emb, Sterile F0/Fertility Problems, Ste
60	Bm1_05895	Gex interacting protein protein 16, isoform d-related	*gei-16*	Lva, Emb, Lvl, Gro, WT
60	Bm1_42370	hypothetical protein	T21B10.3	Clr, Emb, Pvl, Unc, Rup, WT, Gro
59	Bm1_55805	hypothetical protein	K11B4.1	Lva, Emb, Unclassified, WT
58	**Bm1_03495**	Kunitz/Bovine pancreatic trypsin inhibitor domain containing protein	*bli-5*	Bli, Lvl, Unc, Adl, WT
58	Bm1_10140	Zinc finger, C2H2 type family protein	ZK867.1	Let, Egl, Unc, WT
58	Bm1_21225	hypothetical protein	F36A2.7	Lva, General Pace of Development abnormal, Sle, Emb, Unclassified, Gro
57	Bm1_33565	conserved hypothetical protein	*zwl-1*	Emb, WT
57	Bm1_55375	carbamoyl-phosphate synthase, large subunit, putative	*pyr-1*	Emb, Mlt, Lvl, Unc, WT, Gro
57	Bm1_56035	hypothetical protein	*pry-1*	Mig, WT
57	Bm1_37760	Zinc finger, C2H2 type family protein	*mep-1*	Pvl, Lvl, Ste, WT, Stp, Gro
55	Bm1_43170	Transmembrane amino acid transporter protein	Y32F6A.4	Unclassified, WT
55	Bm1_37230	conserved hypothetical protein	Y11D7A.9	Dpy, Egl, Clr, Lvl, Unclassified, Unc, Bmd, Lva, WT, Gro
54	Bm1_09225	HIT zinc finger family protein	*tag-143*	WT, Developmental Delay

54	Bm1_36315	spliced leader 175 kDa protein, putative	T13F2.7	Lva, Emb, Unclassified, WT, Gro
53	Bm1_08450	hypothetical protein	ZK1193.5	Lva, Clr, Lvl, Larval Arrest-Late (L3/L4), Gro
53	Bm1_41500	conserved hypothetical protein	*gei-4*	Let, Sck, Emb, Pvl, Ste, WT
52	Bm1_42535	Tudor domain containing protein	*ekl-1*	Spindle Elongation/Integrity abnormal, Emb, Abs, WT
52	Bm1_51010	hypothetical protein	K03B4.1	Lva, Emb, WT, Gro
52	Bm1_36555	collagen col-34 - Caenorhabditis elegans, putative	*col-34*	Dpy, Bmd, Unc, WT
51	Bm1_33065	hypothetical protein	F46C8.7	Unclassified, WT
51	Bm1_29610	hypothetical protein	Y49G5B.1	Emb, WT
51	Bm1_54230	zgc:101038 protein-related	Y69A2AR.21	Emb, WT
51	Bm1_39200	hypothetical protein	*pes-8*	Prz, Egl, Ste, Adl, WT, Stp
51	Bm1_27205	hypothetical protein	Y47D3B.1	Let, Lva, Egl, Unclassified, WT
51	Bm1_05160	Troponin T.-related	*tnt-2*	Slu, Ste, Unc, Stp, Gro, WT
50	Bm1_55745	sulfakinin receptor protein, putative	T23B3.4	Emb, Unclassified, WT
50	Bm1_39610	hypothetical protein	F40F11.2	Sck, Emb, WT
49	Bm1_11590	Hypothetical 30.1 kDa protein ZC434.4 in chromosome I.-related	ZC434.4	Sle, Emb, Ste, Lva, Sck, Gro, Stp, WT
49	Bm1_22470	conserved hypothetical protein	W02B8.6	Emb, WT
49	Bm1_52795	hypothetical protein	K09H11.3	Cortical Dynamics abnormal, Emb, Ste
49	Bm1_23555	UcrQ family protein	F45H10.2	Clr, WT, Stp, Gro
49	Bm1_47145	Cuticle collagen dpy-2 precursor, putative	*dpy-2*	Dpy, Bmd, WT
49	Bm1_45220	CG7038-PA-related	W04B5.4	Lva, Emb, Larval Lethal-Early (L1/L2), Gro, WT
48	Bm1_32990	Apical junction molecule protein 1, isoform d-related	*ajm-1*	Emb, Unc, Gro, WT
48	Bm1_08735	hypothetical protein	B0035.15	Unc, Gro, WT
48	Bm1_52640	hypothetical protein	Y57E12AL.6	Sck, Emb, Lvl, Passage Through Meiosis defective, Ste
48	Bm1_16540	hypothetical protein	Y54G9A.7	Emb, WT
48	Bm1_20120	PDZ domain containing protein	C25G4.6	Lva, Pvl, Ste, WT, Stp
48	Bm1_40975	hypothetical protein	Y23H5A.3	Led, Slu, Emb, WT
47	Bm1_41700	Zn-finger in Ran binding protein and others containing protein	*npp-7*	Pronuclear/Nuclear Appearance abnormal, Pna, Emb
47	Bm1_12550	hypothetical protein	*csn-1*	Cyk, Sck, Emb, WT, Stp
47	Bm1_16780	hypothetical protein	C44C1.1	Lvl, Sma, Unc, Gro, WT
46	Bm1_14325	hypothetical protein	M05B5.2	Unclassified, Lon, Unc, Gro, WT
46	Bm1_00815	Cuticle collagen 14, putative	*col-14*	Pvl, WT
46	**Bm1_01990**	chitin synthase 2 (chs-2) fragment	*chs-2*	Bmd, Emb, Unc, Ste, WT, Gro
45	Bm1_25060	Nematode cuticle collagen N-terminal domain containing protein	*col-180*	Bmd, Unc, Larval Lethal-Early (L1/L2), WT, Gro

45	Bm1_51260	Innexin family protein	*inx-13*	Unc, Rup, Stp, Gro, WT
45	Bm1_46165	Chain A, Structure Of A Brca2-Dss1 Complex., putative	Y119D3B.15	Sck, Ste, WT
44	**Bm1_17930**	chitin synthase 2 (chs-2) fragment	*chs-2*	Bmd, Emb, Unc, Ste, WT, Gro
44	Bm1_32875	Calponin homolog OV9M.-related	C53C9.2	Gro, WT
43	Bm1_25645	hypothetical protein	ZC247.1	Sck, Ste, Sma, WT, Stp, Gro
42	Bm1_08745	hypothetical protein	C44B12.5	Emb, Sterile F0/Fertility Problems, Ste, WT, Stp
42	Bm1_09360	conserved hypothetical protein	*gei-16*	Lva, Emb, Lvl, Gro, WT
42	Bm1_36270	TspO/MBR family protein	C41G7.3	Him, General Pace of Development abnormal, WT
42	Bm1_43045	Dumpy : shorter than wild-type protein 10, isoform b, putative	*dpy-10*	Dpy, Pch, Slu, WT
42	Bm1_34840	collagen col-34 - Caenorhabditis elegans, putative	*col-108*	Dpy, Emb, Lvl, Unclassified, Unc, Bmd, Lva, WT, Larval Lethal-Early (L1/L2)
42	Bm1_23570	conserved hypothetical protein	K10G9.2	Prz, Pvl, Unc, Adl, Gro, WT
42	Bm1_36575	conserved hypothetical protein	Y55H10A.1	Osmotic Integrity defective, Sck, Sterile F0/Fertility Problems, Lvl, Ste
41	Bm1_26170	hypothetical protein	Y47D3B.1	Let, Lva, Egl, Unclassified, WT
41	Bm1_28210	hypothetical protein	C44C1.1	Lvl, Sma, Unc, Gro, WT
41	Bm1_37610	Destabilase family protein	C55F2.2	Ste, WT
41	Bm1_23080	ATP synthase f chain, mitochondrial.-related	R53.4	Bmd, Lva, General Pace of Development abnormal, Emb, Unclassified, Pvl, Ste
41	Bm1_42700	hypothetical protein	D1043.1	Bmd, Lva, Clr, Emb, Unc, WT
41	Bm1_28670	hypothetical protein	ZK1236.5	Clr, Gro, WT
41	Bm1_24580	ATP synthase e chain, mitochondrial.-related	R04F11.2	Lva, Emb, Unclassified, WT, Gro
40	Bm1_12855	hypothetical protein	*sas-5*	Spindle Assembly abnormal, Emb
40	Bm1_22670	conserved hypothetical protein	F15B9.5	Unclassified, WT
39	Bm1_50710	hypothetical protein	Y32G9B.1	Ste, WT
39	Bm1_18000	Zinc finger, C2H2 type family protein	F10B5.3	Unclassified, WT, Stp
39	Bm1_51520	hypothetical protein	Y59A8B.20	Lon, WT
39	Bm1_24165	TolA protein.-related (similar to sut-1, involved in trans-splicing)	T13B5.8	Emb, WT
38	Bm1_27515	hypothetical protein	Y65B4BR.8	Bmd, Emb, Unclassified, Pvl, Unc, WT
38	Bm1_08545	Mediator protein 4-related	*mdt-4*	Egl, Pch, Emb, Rup, WT
38	Bm1_02565	40S ribosomal protein S12.-related	*rps-12*	Dpy, Slu, Emb, Severe Pleiotropic Defects, Ste, Lva, Sck, WT
38	Bm1_04880	NADH-ubiquinone oxidoreductase AGGG subunit homolog, mitochondrialprecursor .-related	F44G4.2	Lva, General Pace of Development abnormal, Emb, Unclassified
38	Bm1_52140	P40-related	Y69E1A.2	Emb, WT

38	Bm1_00490	CEH-25 homeobox protein-related	*unc-62*	Ooc, Dpy, Egl, Mig, Emb, Unclassified, Pvl, Unc, WT
38	Bm1_18060	hypothetical protein	C16B8.4	Emb, WT
38	Bm1_50825	hypothetical protein	W06A7.2	Unc, WT
38	Bm1_37860	NADH-dependent xylose reductase.-related	F53F1.2	Prz, Lvl, Unc, WT, Gro
37	Bm1_23385	conserved hypothetical protein	C01B7.1	Lva, Emb, WT, Gro
37	Bm1_37570	predicted protein	*rig-1*	Sck, Emb, WT, Stp
37	**Bm1_04665**	2,3-bisphosphoglycerate-independent phosphoglycerate mutase, putative	F57B10.3	Egl, Age, Emb, Lvl, Unc, Bmd, Gro, WT
37	Bm1_18340	LIN-7, putative	*lin-7*	Clr, WT, Gro
37	Bm1_54115	RH01479p-related	Y95D11A.1	Unclassified, Larval Arrest-Late (L3/L4), WT, Stp, Gro
37	Bm1_54490	hypothetical protein	F42G8.5	Unclassified, WT
37	Bm1_00215	conserved hypothetical protein	Y75B8A.14	Emb, WT
37	Bm1_27330	conserved hypothetical protein	B0432.3	Clr, Emb, Lvl, Lva, Sck, Larval Arrest-Early (L1/L2), Stp, Gro, WT
37	Bm1_27330	conserved hypothetical protein	B0432.3	Clr, Emb, Lvl, Lva, Sck, Larval Arrest-Early (L1/L2), Stp, Gro, WT
37	Bm1_15680	hypothetical protein	M176.3	Emb, Sterile F1, WT
37	Bm1_27280	hypothetical protein	*sys-1*	Emb, Pvl, Unc, Rup, Bmd, Led, Lva, WT, Gro, Stp
37	Bm1_55000	Troponin T, putative	*tnt-2*	Slu, Ste, Unc, Stp, Gro, WT
37	Bm1_43955	hypothetical protein	*rod-1*	Sck, Emb, WT
36	Bm1_08225	conserved hypothetical protein	T08A11.2	Emb, Pvl, Unc, Ste, Adl, WT
36	Bm1_13550	conserved hypothetical protein	M04F3.1	Egl, Emb, Pvl, Rup, Unc, Sterile F1, WT, Stp
36	Bm1_43515	Hypothetical 36.5 kDa protein C56G2.3 in chromosome III.-related	C56G2.3	Stp, Gro, WT
36	Bm1_25440	hypothetical protein	T09B4.9	Lva, General Pace of Development abnormal, Emb, Ste, Etv, Gro
36	Bm1_19985	cuticle collagen 2 precursor, putative	*col-97*	Dpy, Bmd, Unc, WT
35	Bm1_36335	conserved hypothetical protein	F49C12.11	Dpy, Lva, Lvl, Unc, WT
35	Bm1_40905	Putative glutamate synthase, putative	W07E11.1	Lva, Emb, Gro, WT
35	Bm1_50900	Conserved hypothetical protein, putative	T21G5.4	Lva, Pvl, WT, Stp
34	Bm1_22525	DNA-(Apurinic or apyrimidinic site) lyase-related	*apn-1*	Emb, WT

34	Bm1_19065	conserved hypothetical protein	*pqn-22*	Emb, WT
34	Bm1_29140	hypothetical protein	W03F11.3	Emb, WT
34	Bm1_17810	vacuolar ATP synthase subunit H, putative	*vha-17*	Osmotic Integrity defective, Lva, Sck, Emb, Lvl, Ste
34	Bm1_56515	Nematode cuticle collagen N-terminal domain containing protein	*col-84*	Sister Chromatid Separation abnormal (Cross-eyed), Emb, Unclassified, WT
34	Bm1_09670	NADH-ubiquinone oxidoreductase B22 subunit .-related	C16A3.5	Lva, Emb, Unclassified, WT, Larval Lethal-Early (L1/L2), Gro
33	Bm1_46400	Nematode cuticle collagen N-terminal domain containing protein	*rol-6*	Dpy, Egl, WT
33	Bm1_55790	hypothetical protein	D2030.3	Lva, Larval Arrest-Early (L1/L2), Unclassified, WT, Gro
33	Bm1_13030	conserved hypothetical protein	*icl-1*	Lva, Emb, Ste, Unc, WT, Gro
32	Bm1_10505	cuticle collagen 34, putative	*col-125*	Dpy, Larval Lethal-Late (L3/L4), Lvl, Unclassified, Unc, Bmd, WT, Larval Lethal-Early (L1/L2)
32	Bm1_48195	Innexin family protein	*inx-3*	Lva, Unc, Gro, WT
32	Bm1_31780	Clc-4 protein., putative	K10D6.2	Emb, WT
32	Bm1_49770	Mitochondrial ribosomal protein L51 / S25 / CI-B8 domain containing protein	C25A1.13	Emb, WT
32	Bm1_00720	conserved hypothetical protein	C44B9.2	Gro, WT
31	Bm1_12400	hypothetical protein	F10E7.6	Clr, Sck, Larval Arrest-Early (L1/L2), WT, Gro
31	Bm1_23180	ribosomal protein L32 containing protein	C30C11.1	Lva, Emb, Unclassified, Larval Lethal-Early (L1/L2), WT, Gro
31	Bm1_09610	Troponin T, putative	*tnt-2*	Slu, Ste, Unc, Stp, Gro, WT
31	Bm1_16685	hypothetical protein	*hcp-4*	Sister Chromatid Separation abnormal (Cross-eyed), Emb, Ste, Mul
31	Bm1_52255	TB2/DP1, HVA22 family protein	C36H8.1	Slu, WT
31	Bm1_07780	immunogenic protein 3, putative	ZK856.7	Developmental Delay, WT
31	Bm1_45405	LD03534p-related	T12G3.5	Lva, Unclassified, WT, Gro
30	Bm1_41030	conserved hypothetical protein	*pqn-38*	Dpy, Pch, Pace of P-Lineage abnormal, Emb, Unc, Rup, Gro, WT
30	Bm1_52065	hypothetical protein	*pbs-5*	Lva, Ocs, Emb, Passage Through Meiosis defective, Unc, Ste
30	Bm1_23010	hypothetical protein	CD4.3	Lva, Emb, Unclassified, WT, Gro
30	Bm1_57645	conserved hypothetical protein	*patr-1*	Emb, Unclassified, Lvl, WT
30	Bm1_07925	peroxisomal membrane anchor protein, putative	*prx-14*	Lva, Larval Arrest-Early (L1/L2), WT, Gro
29	Bm1_09975	hypothetical protein	C44B7.3	Egl, WT
29	Bm1_52560	hypothetical protein	T10G3.1	Emb, WT

29	Bm1_55970	hypothetical protein	C23G10.8	Emb, Pvl, Ste, WT, Gro
29	Bm1_27240	hypothetical protein	T09A5.9	Emb, Nuclear Appearance abnormal, Pvl, Ste, Unc, Stp
29	Bm1_14115	hypothetical protein	*ain-1*	Egl, Unc, WT
29	Bm1_04775	hypothetical protein	H35B03.2	Lva, Unclassified, Larval Arrest-Late (L3/L4), Stp, Gro
29	Bm1_47280	WD-repeat protein WDC146.-related	F25B5.7	Let, Unclassified, Rup, WT
28	Bm1_05925	EGF-like domain containing protein	*mup-4*	Prz, Lva, Sck, Ste, Adl, WT, Larval Lethal-Early (L1/L2)
28	Bm1_49935	Zn-finger in Ran binding protein and others containing protein	C27H5.3	Emb, WT
28	Bm1_21655	hypothetical protein	*lpd-9*	Lva, Emb, Lvl, Larval Arrest-Late (L3/L4), Lpd, WT, Gro
28	Bm1_19420	hypothetical protein	C16C10.6	Lva, Emb, WT, Gro
28	Bm1_07450	hypothetical protein	C07D8.3	Lva, Emb, Unc, WT
27	Bm1_54105	hypothetical protein	*tag-319*	Emb, Ste, WT, Stp
27	Bm1_12525	RNA dependent RNA polymerase family protein	*ego-1*	Cyk, Emb, WT, Gro
27	Bm1_13915	Nematode astacin protease protein 9, isoform c-related	*nas-9*	Emb, WT
27	Bm1_39000	predicted protein	K04C2.5	Sck, Lvl, WT, Stp, Gro
26	Bm1_54705	Nematode cuticle collagen N-terminal domain containing protein	*col-17*	Dpy, Sck, WT
26	Bm1_05210	hypothetical protein	*dpy-7*	Dpy, Egl, Unc, WT, Gro
26	Bm1_56880	hypothetical protein	Y38E10A.24	Clr, Sck, Emb, Gro
26	Bm1_17365	RNA-directed RNA polymerase 1-related	*ego-1*	Cyk, Emb, WT, Gro
26	Bm1_40345	conserved hypothetical protein	F59B10.3	Emb, WT
26	Bm1_52595	Ground-like domain containing protein	*grl-4*	Gro, WT
26	Bm1_38270	predicted protein	*tag-273*	Unclassified, Stp, WT
26	Bm1_06665	Transmembrane amino acid transporter protein	F21D12.3	Emb, WT
25	Bm1_39335	conserved hypothetical protein	B0310.1	Unclassified, WT
25	Bm1_41530	BED zinc finger family protein	*gei-13*	Rol, Dpy, Unc, WT
25	Bm1_00640	hypothetical protein	*ifg-1*	Pch, Emb, Severe Pleiotropic Defects, Ste, Bmd, Lva, Sterile F0/Fertility Problems, Gro, WT
25	Bm1_06810	Cuticle collagen dpy-7 precursor, putative	*dpy-7*	Dpy, Egl, Unc, WT, Gro
25	Bm1_18685	conserved hypothetical protein	W01A8.4	Bmd, Lva, Emb, WT
25	Bm1_49180	NADH-ubiquinone oxidoreductase 15 kDa subunit.-related	Y54E10BL.5	Unclassified
25	Bm1_19740	Hypothetical protein	K10D6.2	Emb, WT
25	Bm1_45285	CG13018-PA, putative	Y18D10A.16	Gro, WT

25	Bm1_53210	predicted protein	T19A5.3	Lvl, WT
25	Bm1_37315	hypothetical protein	*hmg-5*	WT, Stp
25	Bm1_07795	hypothetical protein	F45E12.5	Emb, WT, Stp
24	Bm1_11840	hypothetical protein	K07B1.6	Lva, WT
24	Bm1_21815	hypothetical protein	F10E9.4	Lva, Unclassified, Lvl, Gro, WT
24	Bm1_32260	conserved hypothetical protein	C15C7.5	Clr, Unclassified, Unc, WT, Gro
24	Bm1_04070	Salivary glue protein Sgs-3 precursor.-related	H11E01.3	Emb, WT
24	Bm1_50790	hypothetical protein	*spd-2*	Lva, Spd, Spindle Assembly abnormal, Emb, Unclassified, Ste
24	Bm1_22765	hypothetical protein	ZK484.4	Emb, WT, Stp
24	Bm1_22905	hypothetical protein	ZK858.5	Emb, WT
24	Bm1_38370	predicted protein	*flp-1*	Aldicarb resistant, WT
23	Bm1_15075	PDZ domain containing protein	T21G5.4	Lva, Pvl, WT, Stp
23	Bm1_14650	hypothetical protein	F23H11.2	Ste, Sma, WT, Stp
23	Bm1_55755	major sperm protein, putative	*ssp-16*	Unclassified, WT
23	Bm1_55755	major sperm protein, putative	*ssp-19*	Unclassified, WT
23	Bm1_53230	hypothetical protein	C26F1.3	Gro, WT
23	Bm1_53630	hypothetical protein	F09E5.11	Prz, Clr, Lvl, Unc, Lva, Larval Arrest-Early (L1/L2), Gro, WT
23	Bm1_09930	kinesin light chain, putative	*klc-2*	Emb, WT
22	Bm1_23380	Zinc finger, C2H2 type family protein	C01B7.1	Lva, Emb, WT, Gro
22	Bm1_15990	RE06140p-related	T21B10.1	Lva, Clr, Emb, Unclassified, Sma, WT, Gro
22	Bm1_09120	Hypothetical protein	E01A2.6	Emb, WT
22	Bm1_46225	hypothetical protein	C05B5.4	Gro, WT
22	Bm1_21885	SWIB/MDM2 domain containing protein	T24G10.2	Emb, WT
22	Bm1_46355	hypothetical protein	*emb-30*	Emb, Pvl, Passage Through Meiosis defective, Stp, WT
22	Bm1_28165	hypothetical protein	C26B9.3	Egl, Pvl, Unc, WT
22	Bm1_01765	Probable mitochondrial import receptor subunit TOM7-like	*tomm-7*	Unclassified, WT
22	Bm1_01505	Hypothetical protein	R186.3	Osmotic Integrity defective, Emb, WT
22	Bm1_44095	Conserved hypothetical protein, putative	F11G11.5	Emb, WT
21	Bm1_19440	hypothetical protein	C06A8.2	Clr, Pch, Emb, Unclassified, Lva, Gro, Stp, WT
21	Bm1_34110	hypothetical protein	K12H4.5	Lva, Emb, WT, Larval Lethal-Early (L1/L2), Gro
21	Bm1_36295	Resistance to inhibitors of cholinesterase protein 3-related	*ric-3*	Lva, Emb, Unclassified, WT

21	Bm1_06340	hypothetical protein	K02E10.4	Unclassified, WT
21	Bm1_50365	conserved hypothetical protein	F09G2.9	Emb, Ste, WT
21	Bm1_41110	conserved hypothetical protein	C45G9.11	Sterile F0/Fertility Problems, WT
21	Bm1_53505	myotactin form B, putative	*let-805*	Prz, Sck, Emb, Lvl, Adl, Ste, WT
20	Bm1_35045	hypothetical protein	H05L03.3	Unclassified, WT
20	Bm1_38610	conserved hypothetical protein	*tnt-4*	Larval Arrest-Early (L1/L2), WT
20	Bm1_13170	conserved hypothetical protein	K04C2.2	Pch, Sck, Pvl, Lvl, Ste, WT, Stp, Gro
20	Bm1_32180	hypothetical protein	F55A12.2	Gro, WT
20	Bm1_07440	hypothetical protein	Y53F4B.13	Ste, WT
20	Bm1_26400	Fibronectin type III domain containing protein	*ptp-3*	Emb, Morphology defect, WT
20	Bm1_25025	Spc97 / Spc98 family protein	*gip-1*	Spindle Assembly abnormal, Emb, WT
20	Bm1_43720	conserved hypothetical protein	*eif-3.F*	Dpy, Emb, Unclassified, Pvl, Bmd, Lva, WT, Stp, Gro
20	Bm1_10260	gene model 83, putative	ZK430.7	Lva, Emb, Pvl, Larval Arrest-Late (L3/L4), Ste, WT, Gro
20	Bm1_37335	ankyrin-related unc-44-related	*unc-44*	Dpy, Slu, Unc, Sma, Gro, WT
19	Bm1_20495	hypothetical protein	Y40B1B.5	Gro, WT
19	Bm1_33635	hypothetical protein	R09F10.3	Emb, Ste, WT
19	Bm1_54575	Membrane calcium atpase protein 3, isoform a, putative	*mca-3*	Prz, Adl, WT
19	Bm1_03645	Warthog protein-related	*wrt-4*	Emb, WT
18	Bm1_10215	Calcium-binding protein.-related	C01G8.9	Let, Emb, Lvl, Ste, Rup, Bmd, Led, WT, Gro
18	Bm1_00120	ATP synthase epsilon chain, mitochondrial, putative	F32D1.2	Emb, Severe Pleiotropic Defects, WT, Stp, Gro
18	Bm1_21210	Innexin inx-14.-related	*inx-3*	Lva, Unc, Gro, WT
18	Bm1_01060	Calcium-binding protein.-related	K01A6.4	Emb, Unc, Gro, WT
18	Bm1_45055	Chitin binding Peritrophin-A domain containing protein	W03F11.1	Ste, WT
17	Bm1_10475	hypothetical protein	W01A8.5	Emb, Unclassified, Stp, WT
17	Bm1_46520	predicted protein	C54G4.9	Emb, WT
17	Bm1_14055	ShTK domain containing protein	F48G7.5	Sck, Ste, WT, Stp
17	Bm1_22500	hypothetical protein	F20D12.2	Bmd, Sck, Emb, Unclassified, Lvl, Ste, WT
17	Bm1_46015	F-box domain containing protein	K03H1.11	Emb, WT
17	Bm1_06310	protein R52.2-related	C07D8.2	Lva, Emb, Unc, WT
16	Bm1_18740	Transmembrane cell adhesion receptor mua-3 precursor, putative	*mua-3*	Prz, Lvl, Ste, Unc, Lva, Sck, WT, Larval Lethal-Early (L1/L2)
16	Bm1_47930	VAB-10A protein-related	*vab-10*	Prz, Let, Emb, Pvl, Lvl, Ste, Rup, Unc, Bmd, Lva, Gro, Stp, WT

16	Bm1_05890	B20-1 protein, putative	*gei-16*	Lva, Emb, Lvl, Gro, WT
16	Bm1_03770	FLYWCH zinc finger domain containing protein	*peb-1*	Clr, Pvl, Unclassified, Unc, WT
16	Bm1_25120	Tudor domain containing protein	*tudr-1*	Emb, WT
16	Bm1_38875	hypothetical protein	Y62E10A.11	Emb, Pvl, Unc, Ste, Stp, Gro, WT
15	Bm1_13600	major sperm protein 2 , putative cytoskeletal MSP	*msp-40*	Emb, Unclassified, WT
15	Bm1_23015	hypothetical protein, conserved	CD4.4	Aberrant Cytoplasmic Structures, Lva, Clr, Emb, Mlt, Unc, WT, Gro
15	Bm1_13605	Major Sperm Protein (MSP), putative cytoskeletal MSP	*msp-50*	Sck, Emb, Unclassified, Ste, WT
15	Bm1_33105	hypothetical protein	Y57A10A.27	Pch, Unclassified, Lvl, Lva, Sck, Stp, Gro, WT
15	Bm1_22560	hypothetical protein	*dpy-22*	Egl, Pvl, Unc, Sma, WT
15	Bm1_10860	hypothetical protein	Y106G6A.2	Gro, WT
15	Bm1_22820	hypothetical protein	R10H10.4	Aldicarb resistant, Emb, WT
15	Bm1_46970	hypothetical protein	C14A4.12	Unclassified, WT
14	Bm1_38880	Mitochondrial ATP synthase g subunit family protein	*asg-1*	Bmd, General Pace of Development abnormal, Emb, Etv, WT, Stp, Gro
14	Bm1_46130	hypothetical protein	Y37D8A.16	Lva, Unc, Gro, WT
14	Bm1_42730	conserved hypothetical protein	Y54E10A.7	Lva, Emb, WT, Gro
14	Bm1_09160	conserved hypothetical protein	F18C12.3	Gro, WT
14	Bm1_29320	hypothetical protein	*spd-1*	Egl, Pvl, Lon, Unc, Rup, Bmd, Stp, WT, Gro
14	Bm1_57650	conserved hypothetical protein	ZK930.3	Egl, Pvl, Unc, WT
14	Bm1_56480	RhoGAP domain containing protein	K09H11.3	Cortical Dynamics abnormal, Emb, Ste
13	Bm1_26235	hypothetical protein	W02B8.4	Emb, WT
13	Bm1_55690	NADH-ubiquinone oxidoreductase B12 subunit .-related	C18E9.4	Lva, Emb, WT, Gro
13	Bm1_10660	Hypothetical 20.9 kDa protein in PLB1-HXT2 intergenic region.-related	T01D1.4	Emb, WT
13	Bm1_41635	hypothetical protein	R07E5.7	Dpy, Lva, Larval Arrest-Early (L1/L2), Lvl, Unc, WT, Gro
13	Bm1_51715	hypothetical protein	C14C10.4	Lva, Emb, Ste, WT, Gro
13	Bm1_40800	Helix-loop-helix DNA-binding domain containing protein	*hlh-13*	Lva, Emb, Lvl, Unc, WT
13	Bm1_50395	hypothetical protein	*sgo-1*	Lva, Emb, Unclassified, WT
12	Bm1_53760	cytochrome-c oxidase, putative	Y71H2AM.5	Lva, Clr, Emb, WT, Stp, Gro
12	Bm1_28945	hypothetical protein	F33D11.5	Ste, WT
12	Bm1_50000	Conserved hypothetical protein, putative	F02E9.5	Emb, WT

12	Bm1_53515	conserved hypothetical protein	*let-805*	Prz, Sck, Emb, Lvl, Adl, Ste, WT
12	Bm1_33500	hypothetical protein	Y54E10A.16	Prz, Egl, WT
12	Bm1_36265	conserved hypothetical protein	*smn-1*	Emb, Unclassified, Lvl, Unc, Lva, Sck, WT, Stp, Gro
12	Bm1_29880	Ubiquitin carboxyl-terminal hydrolase family protein	Y67D2.2	Emb, Lvl, WT
12	Bm1_52720	Conserved hypothetical protein, putative	*lpl-1*	Lva, Sck, Emb, Sma, WT, Stp, Gro
12	Bm1_21695	hypothetical protein	E01G4.2	Lva, Larval Lethal-Late (L3/L4), Ste, WT
12	Bm1_23935	heavy metal-associated domain containing protein	*cuc-1*	Unclassified, WT
12	Bm1_06925	F26F3.2 protein -related	*ndg-4*	Bmd, Emb, WT
12	Bm1_07615	Peroxin-3 family protein	C15H9.10	General Pace of Development abnormal, Larval Arrest-Early (L1/L2), WT
12	Bm1_46775	hypothetical protein	T24F1.2	Pronuclear/Nuclear Appearance abnormal, WT
11	Bm1_29000	hypothetical protein	*tag-286*	Emb, WT
11	Bm1_27615	bZIP transcription factor family protein	*fos-1*	Pvl, Ste, Rup, WT, Stp
11	Bm1_32720	hypothetical protein	C29H12.6	Unclassified, WT
11	Bm1_07485	hypothetical protein	K04G7.11	Emb, Unclassified, Pvl, Unc, Rup, Bmd, Lva, Sck, WT, Gro, Stp
11	Bm1_26745	hypothetical protein	C23H3.2	Unclassified, WT
11	Bm1_18115	hypothetical protein	*npp-15*	Emb, WT
11	Bm1_00910	Zinc finger, C2H2 type family protein	F10B5.3	Unclassified, WT, Stp
11	Bm1_43075	hypothetical protein	C50F2.2	Emb, WT
11	Bm1_02140	Lethal protein 805, isoform d, putative	*let-805*	Prz, Sck, Emb, Lvl, Adl, Ste, WT
10	Bm1_53390	Ubiquinone biosynthesis protein COQ4 homolog, putative	*coq-4*	Age, Unclassified, WT
10	Bm1_52605	hypothetical protein	C50E3.5	Emb, WT, Stp
10	Bm1_05820	hypothetical protein	B0240.4	Pronuclear/Nuclear Appearance abnormal, WT
10	Bm1_54895	hypothetical protein	C08C3.4	Ste, WT
10	Bm1_37705	NHR1 homology to TAF family protein	*taf-4*	Lva, Emb, Lvl, Unc, Ste, Gro, WT
10	Bm1_16040	hypothetical protein	F01F1.3	Emb, WT
9	Bm1_02065	Abnormal cell migration protein 10, putative	*mig-10*	Egl, Emb, Unc, Gro, WT
9	Bm1_41775	UBA/TS-N domain containing protein	*pqn-59*	Egg Size abnormal, Emb, Unclassified, Lvl, Unc, Ste, Lva, Sck, WT

9	Bm1_19380	hypothetical protein	F42G4.6	Aldicarb resistant, WT
9	Bm1_56530	28S ribosomal protein S30, mitochondrial, putative	B0511.8	Lva, Emb, Pvl, Ste, Sma, WT
9	Bm1_30695	hypothetical protein	R04F11.5	Emb, WT
9	Bm1_32280	hypothetical protein	*tag-343*	Unc, Gro, WT
9	Bm1_24615	ATP synthase B chain, mitochondrial precursor, putative	*asb-2*	Lva, General Pace of Development abnormal, Emb, Unclassified, Ste, Stp, Gro, WT
8	Bm1_09195	Thyroglobulin type-1 repeat family protein	*mlt-11*	Lva, Emb, Unc, WT
8	Bm1_42645	hypothetical protein	*mut-7*	Lva, WT
8	Bm1_01030	hypothetical protein	T08H4.2	Emb, WT
8	Bm1_50115	Hypothetical UPF0172 protein CG3501.-related	F25H2.4	Clr, Emb, Unclassified, Unc, Etv, Complex Phenotype, Lva, Gro, Stp, WT
7	Bm1_26495	hypothetical protein	T07C12.12	Emb, WT
7	Bm1_46050	Zinc finger, C2H2 type family protein	ZC395.8	Sck, Unc, WT, Stp
7	Bm1_01100	hypothetical protein	T06A10.2	Clr, Emb, Larval Arrest-Early (L1/L2), Lon, Gro, WT
7	Bm1_27360	hypothetical protein	T14B4.1	Emb, WT
7	Bm1_35060	Troponin family protein	*tni-3*	Let, Unclassified, WT
7	Bm1_10425	hypothetical protein	C06A5.3	Pvl, Stp, WT
6	Bm1_44890	hypothetical protein	W02D3.10	Emb, WT
6	Bm1_42145	OTU-like cysteine protease family protein	*duo-1*	Aldicarb resistant, WT
6	Bm1_06460	hypothetical protein	ZC513.1	Unclassified, WT
6	Bm1_56405	hypothetical protein	C31C9.2	Emb, Gro, WT
6	Bm1_23670	FRG1 protein homolog, putative	ZK1010.3	Lva, Gro, WT
6	Bm1_34425	Ctr copper transporter family protein	F27C1.2	Dpy, Ste, Sma, WT, Stp, Gro
6	Bm1_08610	Hypothetical protein	*unc-10*	Aldicarb resistant, WT
6	Bm1_05190	hypothetical protein	T09B4.2	Emb, WT
6	Bm1_13195	hypothetical protein	B0495.2	Emb, WT
6	Bm1_18845	GRIM-19 protein	C34B2.8	Lva, Sle, Emb, WT, Gro
5	Bm1_19290	hypothetical protein	*npp-16*	Larval Arrest-Early (L1/L2), WT
5	Bm1_01955	LPXTG cell wall surface anchor family protein, putative	*tbx-33*	Bmd, Lva, Emb, Sterile F0/Fertility Problems, Unc, WT
5	Bm1_17130	Kunitz/Bovine pancreatic trypsin inhibitor domain containing protein	*mlt-11*	Lva, Emb, Unc, WT
4	Bm1_09640	Nematode cuticle collagen N-terminal domain containing protein	*dpy-9*	Dpy, Unc, WT
4	Bm1_57420	Zinc finger, C2H2 type family protein	ZK867.1	Let, Egl, Unc, WT
4	Bm1_09565	Transmembrane amino acid transporter protein	Y32F6A.4	Unclassified, WT

4	Bm1_03010	PRO0477p-related	Y50D7A.9	Unclassified, WT
4	Bm1_10315	Long protein 1, isoform b, putative	*lon-1*	Sck, Emb, WT
4	Bm1_35660	Succinate dehydrogenase, putative	F33A8.5	Lva, Clr, Emb, WT, Gro
4	Bm1_21040	Hypothetical thiol protease C06G4.2 in chromosome III.-related	*clp-1*	Ced, WT
4	Bm1_06045	conserved hypothetical protein	K06C4.15	Lva, Emb, Unc, WT
3	Bm1_49240	hypothetical protein	*irs-2*	Unclassified, Gro, WT
3	Bm1_28060	Hypothetical protein	R12E2.12	Lva, Unclassified, WT
3	Bm1_13520	Hypothetical protein-conserved	C05D11.10	Let, Lva, Emb, Gro, WT
3	Bm1_20745	adenosine deaminase ADR-1C, putative	*adr-1*	Lva, Unclassified, WT
2	Bm1_39165	cAMP-dependent protein kinase regulatory chain, putative	*kin-2*	Dpy, Lvl, Unc, Bmd, Lva, Stp, Larval Lethal-Early (L1/L2), WT
2	Bm1_16530	Hint module family protein	*qua-1*	Lva, Clr, Larval Lethal-Late (L3/L4), Mlt, Unc, WT
2	Bm1_43570	hypothetical protein	F55C5.4	Bli, Sister Chromatid Separation abnormal (Cross-eyed), Emb, Unclassified, Pvl, Unc, Gro
2	Bm1_24565	conserved hypothetical protein	*mps-1*	Emb, WT
2	Bm1_15165	hypothetical protein	C56G2.1	Emb, Sterile F1, WT
2	Bm1_29960	von Willebrand factor type A domain containing protein	M01E10.2	Dpy, Unclassified, WT
1	Bm1_52385	hypothetical protein	Y24D9A.1	Sterile F1, WT
1	Bm1_42980	Immunoglobulin I-set domain containing protein	*oig-2*	Unclassified, WT
1	Bm1_08025	F-box domain containing protein	F54D5.9	Emb, WT
1	Bm1_03155	gag protein-related	F07B7.6	Lva, Emb, Unc, WT
1	Bm1_06290	hypothetical protein	K06C4.14	Emb, WT
1	Bm1_14965	hypothetical protein	R03E1.2	Lva, Emb, Unclassified, Lvl, Unc, WT, Larval Lethal-Early (L1/L2)
1	Bm1_00705	P. falciparum RESA-like protein with DnaJ domain-related	*ssq-2*	Unc, WT
1	Bm1_06655	hypothetical protein	F08G2.7	Unclassified, WT
1	Bm1_19425	MNN4 protein.-related	F46C5.6	Unclassified, WT
1	Bm1_36080	hypothetical protein	*sas-4*	Dpy, Spindle Assembly abnormal, Emb, Unc, Rup, Gro
0	Bm1_02410	hypothetical protein	C33H5.17	Unclassified, WT
0	Bm1_13005	hypothetical protein	*smu-2*	Unclassified, WT
0	Bm1_43725	Mov34/MPN/PAD-1 family protein	*eif-3.F*	Dpy, Emb, Unclassified, Pvl, Bmd, Lva, WT, Stp, Gro
0	Bm1_31870	Surfeit locus protein 5 containing	*mdt-22*	Dpy, Slu, Emb, Pvl, Sma, Lva,

		protein		WT, Gro, Stp
0	Bm1_08175	conserved hypothetical protein	*cdt-1*	Led, Sister Chromatid Separation abnormal (Cross-eyed), Emb, Pvl, Ste
0	Bm1_00430	hypothetical protein	F54D7.7	Aldicarb resistant, WT
0	Bm1_28625	Mitochondrial glycoprotein	F59A2.3	Rup, Unc, Gro, WT
0	Bm1_28315	conserved hypothetical protein	T16G12.5	Pvl, Unc, Rup, Stp, Gro, WT
0	Bm1_32025	hypothetical protein	C54G7.1	Unclassified, WT
0	Bm1_03765	hypothetical protein	C15H9.7	Unclassified, WT
-1	Bm1_51735	Troponin I, putative	*tni-4*	WT, Gro
-1	Bm1_13150	Barrier-to-autointegration factor 1, putative	*baf-1*	Bmd, Emb, Unclassified, Unc, WT
-1	Bm1_27705	7B2-related	*sbt-1*	Aldicarb resistant, WT
-2	Bm1_34300	Nematode cuticle collagen N-terminal domain containing protein	*dpy-8*	Rol, Dpy, Egl, Unc, Sma, WT
-2	Bm1_22990	actin-depolymerizing factor 1, putative	*unc-60*	Let, Sck, Pvl, Unclassified, Ste, Unc, WT
-2	Bm1_25910	RIKEN cDNA 2610002M06, putative	F23C8.6	Let, Emb, Unc, Aberrant Cytoplasmic Structures, Lva, aldicarb resistant, Gro, Larval Lethal-Early (L1/L2), WT
-2	Bm1_25810	Hepatocellular carcinoma-associated antigen 127, putative	ZK930.3	Egl, Pvl, Unc, WT
-3	Bm1_33765	hypothetical protein	R08D7.2	Emb, Pvl, Rup, Stp, Gro, WT
-3	Bm1_34145	RE35789p-related	F33D4.7	Lva, Emb, Unclassified, WT
-3	Bm1_14240	PWWP domain containing protein	C06A5.3	Pvl, Stp, WT
-3	Bm1_19990	cuticle collagen 2 precursor, putative	*col-97*	Dpy, Bmd, Unc, WT
-3	Bm1_29435	Hypothetical protein	Y63D3A.8	Sck, Sma, WT, Stp
-4	Bm1_33020	LAMP family protein lmp-1 precursor.-related	*lmp-1*	Clr, WT
-4	Bm1_04725	Helix-loop-helix DNA-binding domain containing protein	*hlh-2*	Emb, Pvl, Unc, WT, Stp
-4	Bm1_35390	Slbp protein, putative	*cdl-1*	Bmd, Sister Chromatid Separation abnormal (Cross-eyed), Emb, Pvl, Ste, Rup, Gro
-4	Bm1_39400	BED zinc finger family protein	F25H8.6	Lva, WT
-4	Bm1_07190	hypothetical protein	F41G3.14	Sck, Larval Arrest-Early (L1/L2), WT
-5	Bm1_05435	conserved hypothetical protein	Y54E10A.2	Prz, Emb, Unclassified, Pvl, Lvl, Rup, Unc, Lva, WT, Gro
-5	Bm1_37515	hypothetical protein	F53A9.4	Unclassified, WT
-5	Bm1_34195	Filamin/ABP280 repeat family protein	C23F12.1	Mlt, Unc, Rup, WT, Gro
-5	Bm1_33855	Ubiquinol-cytochrome C chaperone family protein	C35D10.5	Emb, Larval Lethal-Early (L1/L2), WT, Gro
-5	Bm1_30505	Neurotransmitter-gated ion-channel transmembrane region family protein	F21A3.7	WT, Stp, Gro
-6	Bm1_55395	Conserved hypothetical protein,	*npp-4*	Pronuclear/Nuclear Appearance

		putative		abnormal, Emb, Unclassified, Pvl, Stp, Gro, WT
-6	Bm1_21970	conserved hypothetical protein	Y39A1A.13	Pvl, Sterile F1, Rup, Unc, Stp, WT
-6	Bm1_13050	Hypothetical protein	Y39B6A.36	Emb, Rup, Stp, Gro, WT
-6	Bm1_03920	Cytochrome c oxidase subunit IV family protein	W09C5.8	Lva, Age, Emb, Ste, WT, Gro
-7	Bm1_06785	conserved hypothetical protein	Y71H2B.2	Sister Chromatid Separation abnormal (Cross-eyed), Emb, WT
-7	Bm1_22805	Hypothetical protein	*snr-5*	Lva, Sck, Emb, Lvl, Ste, WT, Larval Lethal-Early (L1/L2)
-7	Bm1_02630	hypothetical protein	C06A8.5	Bmd, Chromosome Segregation (karyomeres) abnormal, Emb, WT, Gro
-7	Bm1_17210	conserved hypothetical protein	R05D11.9	Emb, WT
-8	Bm1_29430	Cytochrome c oxidase polypeptide Vb, mitochondrial precursor.-related	*cco-1*	Age, Clr, Emb, Unclassified, Lpd, Ste, Lva, Gro, WT
-9	Bm1_10835	hypothetical protein	C09H10.7	Osmotic Integrity defective, Emb, Pvl, Passage Through Meiosis defective, Stp
-9	Bm1_31055	RNA recognition motif containing protein, putative	K07H8.10	Emb, WT, Gro
-9	Bm1_44000	NADH-ubiquinone oxidoreductase ASHI subunit, mitochondrial precursor, putative	Y51H1A.3	Emb, Unclassified, WT
-9	Bm1_41915	NADH-ubiquinone oxidoreductase 18 kDa subunit, mitochondrial precursor.-related	*lpd-5*	Lva, Emb, WT
-9	Bm1_16675	hypothetical protein	F26E4.4	Lva, Emb, Larval Arrest-Early (L1/L2), WT
-9	Bm1_36460	hypothetical protein	Y51H4A.15	Lva, Pch, Slu, Emb, Unclassified, WT, Gro
-10	Bm1_52475	hypothetical protein	T01C3.1	Emb, Pvl, Rup, Unc, Sma, Sck, Gro, WT
-10	Bm1_01075	CG15780-PA-related	Y73B3A.17	Larval Arrest-Late (L3/L4), WT
-10	Bm1_07885	SD09147p-related	F25H5.6	Lva, Unclassified, Gro, WT
-10	Bm1_40540	conserved hypothetical protein	C39E9.11	Emb, Unc, WT
-11	Bm1_42620	DNA segment, Chr 7, Wayne State University 180, expressed, putative	*crn-5*	Lva, Pch, Larval Arrest-Early (L1/L2), WT, Gro
-11	Bm1_33770	zgc:92910-related	ZK265.6	Lva, Larval Arrest-Early (L1/L2), Unclassified, Pvl, Larval Arrest-Late (L3/L4), WT, Gro
-11	Bm1_36075	D10Ertd718e protein-related	F10E9.11	Spindle Assembly abnormal, Emb, WT
-12	Bm1_49670	zgc:101594-related	Y47G6A.9	Lva, Clr, Pvl, Rup, WT, Stp, Gro
-12	Bm1_48075	M-phase phosphoprotein 6.-related	F29A7.6	Lva, Pch, Pvl, WT, Stp, Gro
-12	Bm1_04435	Hypothetical protein	*vha-15*	Prz, Bli, Lva, Emb, Lvl, WT

-12	Bm1_55635	hypothetical protein	*mdf-1*	Pvl, Unc
-13	Bm1_57435	ELM2 domain containing protein	*egl-27*	Egl, Emb, Lvl, Rup, Unc, Bmd, Lva, Larval Lethal-Early (L1/L2), WT
-13	Bm1_14750	Ubiquinol-cytochrome C reductase hinge protein	T27E9.2	Gro, WT
-13	Bm1_34560	CGI-115 protein-related	F53E10.6	WT, Gro
-13	Bm1_46380	Probable dolichol-phosphate mannosyltransferase subunit 3, putative	F28D1.11	Sck, Sma, WT, Stp
-14	Bm1_46795	protein T01B7.5, putative	T01B7.5	Egl, Pch, Ste, Gon, Lva, Gro, Stp, WT
-14	Bm1_46160	Conserved hypothetical protein, putative	D2007.4	Lva, Emb, Larval Lethal-Early (L1/L2), WT, Gro
-14	Bm1_49750	ORF; putative	Y47H9C.7	Dpy, Lva, Emb, WT, Gro
-15	Bm1_47525	LD18634p-related	*egl-46*	Sck, Lvl, WT, Gro
-15	Bm1_12585	small heat shock protein 12.6, putative	*hsp-12.2*	Sterile F0/Fertility Problems, WT
-15	Bm1_37955	hypothetical protein	ZK792.5	Unclassified, Stp, WT
-15	Bm1_18760	conserved hypothetical protein	C56A3.4	WT, Gro
-15	Bm1_02155	Delta5 fatty acid desaturase-related	*fat-3*	Dpy, Unc, WT, Gro
-16	Bm1_01935	Hypothetical protein	Y43F8C.8	Larval Arrest-Early (L1/L2), Gro, WT
-16	Bm1_03840	Hypothetical protein	Y40B1B.7	Emb, Gro, WT, Stp
-17	Bm1_37810	GGL domain containing protein	*gpc-2*	Pnm, Emb, WT
-17	Bm1_16340	hypothetical protein	K04G7.1	Emb, Larval Arrest-Early (L1/L2), Unclassified, Larval Arrest-Late (L3/L4), WT, Stp, Gro
-17	Bm1_27220	hypothetical protein	*pqn-45*	Osmotic Integrity defective, Emb, Sterile F0/Fertility Problems, Ste, Sterility/Impaired Fertility in F0, WT
-18	Bm1_14345	Helix-loop-helix DNA-binding domain containing protein	*hnd-1*	Bmd, WT
-18	Bm1_48760	putative transcription factor	*tag-313*	Unclassified, WT, Gro
-18	Bm1_37370	conserved hypothetical protein	*unc-44*	Dpy, Slu, Unc, Sma, Gro, WT
-18	Bm1_02675	GYF domain containing protein	R10D12.14	Emb, WT
-18	Bm1_02520	1300013D05Rik protein-related	F52C9.7	Dpy, Sle, Emb, WT
-18	Bm1_56915	conserved hypothetical protein	Y51A2D.7	Emb, Ste, Unc, WT, Gro
-19	Bm1_41180	Conserved hypothetical protein, putative	ZK1098.7	Egl, Emb, Lva, Larval Arrest-Early (L1/L2), Stp, WT, Gro, Larval Lethal-Early (L1/L2)

-19	Bm1_10195	Hypothetical protein	C24H12.5	Bmd, Lvl, Unc, WT
-19	Bm1_37250	RNA recognition motif.	F11A10.7	Lva, Emb, WT
-20	Bm1_41995	Conserved hypothetical protein, putative	*rsp-7*	Pch, Slu, Emb, Unclassified, Lvl, Unc, Gro, WT
-20	Bm1_44295	Helix-loop-helix DNA-binding domain containing protein	*hlh-1*	Emb, Unc, WT
-20	Bm1_48330	Dihydrofolate reductase.-related	C36B1.7	Emb, WT
-20	Bm1_45185	putative RNA binding protein	T04A8.6	Egl, Sle, Ste, Lva, Larval Arrest-Early (L1/L2), Gro, Stp, Larval Lethal-Early (L1/L2), WT
-21	Bm1_44035	hypothetical protein	C50B6.2	Emb, Unc, WT
-21	Bm1_28490	conserved hypothetical protein	F25H5.5	Emb, WT
-22	Bm1_26345	Hypothetical protein	*tag-307*	Age, Unclassified, WT
-22	Bm1_09495	conserved hypothetical protein	F30F8.9	Emb, WT
-22	Bm1_17305	Hypothetical protein	*ntl-2*	Emb, Unclassified, Larval Arrest-Late (L3/L4), Unc, Rup, Lva, Adl, WT, Gro, Stp
-22	Bm1_25285	Prion-like--related	*pqn-13*	Aldicarb resistant, WT
-22	Bm1_17120	Hyaluronan / mRNA binding family protein	*vig-1*	Emb, WT
-22	Bm1_54140	GH14561p-related	T27F7.1	General Pace of Development abnormal, Emb, Mlt, Unclassified, Lvl, Sma, Unc, Lva, Adl, WT, Gro
-23	Bm1_19655	Hypothetical protein	F54C4.1	Lva, Emb, Gro, WT
-23	Bm1_42395	Nematode cuticle collagen N-terminal domain containing protein	*col-137*	Unclassified, Gro, WT
-23	Bm1_32145	Cuticle collagen dpy-7 precursor, putative	*dpy-8*	Rol, Dpy, Egl, Unc, Sma, WT
-23	Bm1_31210	Zinc finger, C2H2 type family protein	*lir-1*	Emb, Mlt, Lvl, Unc, WT
-23	Bm1_27030	Talin 1, putative	Y71G12B.11	Clr, Pch, Prl, Emb, Lvl, Pvl, Ste, Rup, Unc, Bmd, Lva, Sck, Gro, WT, Stp
-23	Bm1_27570	ARID/BRIGHT DNA binding domain containing protein	C01G8.9	Let, Emb, Lvl, Ste, Rup, Bmd, Led, WT, Gro
-24	Bm1_26605	UPF0279 protein C14orf129 homolog.-related	T24H7.3	Emb, WT
-24	Bm1_46495	Zinc finger, C2H2 type family protein	*lin-13*	Bmd, Sterile F1, WT
-25	Bm1_04785	AN1-like Zinc finger family protein	F56F3.4	Unclassified, WT
-25	Bm1_01560	conserved hypothetical protein	*dgn-1*	Unclassified, WT
-25	Bm1_49645	Microcephalin., putative	W04A8.1	Lvl, WT
-25	Bm1_45665	Mediator protein 11-related	*mdt-11*	Dpy, Lva, Emb, WT
-26	Bm1_30230	Hypothetical 19.4 kDa protein ZC395.10 in chromosome III.-related	ZC395.10	Emb, Ste, WT

-26	Bm1_45060	hypothetical protein	Y53G8AM.8	Emb, WT
-26	Bm1_15510	RE18450p, putative	M04B2.4	Gro, WT
-26	Bm1_05930	Muscle positioning protein 4, putative	*mup-4*	Prz, Lva, Sck, Ste, Adl, WT, Larval Lethal-Early (L1/L2)
-27	Bm1_47605	CUB domain containing protein	*sol-1*	Unclassified, WT
-27	Bm1_00645	Transmembrane cell adhesion receptor mua-3 precursor, putative	*mup-4*	Prz, Lva, Sck, Ste, Adl, WT, Larval Lethal-Early (L1/L2)
-27	Bm1_43030	protein C18H9.7 , putative	*rpy-1*	Unclassified, WT
-27	Bm1_56230	GH25683p, putative	Y57A10A.10	Unclassified, WT
-27	Bm1_34260	M-phase phosphoprotein, mpp8, putative	T09A5.8	Emb, WT
-28	Bm1_41220	Helix-loop-helix DNA-binding domain containing protein	*hlh-4*	Pvl, Gro, WT
-28	Bm1_34445	zgc:91831-related	K01G5.8	Unclassified, Gro, WT
-28	Bm1_39315	Zinc finger, C2H2 type family protein	Y56A3A.18	Complex Phenotype, Lva, Emb, Stp, Gro, WT
-29	Bm1_33410	CG32584-PB-related	ZK1248.11	Unclassified, WT
-30	Bm1_11825	hypothetical protein	F43D9.1	Dpy, Lva, Emb, Unclassified, WT
-30	Bm1_15840	Surfeit locus protein 6 containing protein	ZK546.14	Lva, Sck, Larval Arrest-Late (L3/L4), WT, Gro
-30	Bm1_13965	M-phase phosphoprotein-related	F54H5.3	Unclassified, WT
-30	Bm1_57145	Fibronectin type III domain containing protein	*let-805*	Prz, Sck, Emb, Lvl, Adl, Ste, WT
-30	Bm1_17070	Leucine Rich Repeat family protein	*pan-1*	Lva, Clr, Lvl, Unc, WT
-31	Bm1_47600	Suppressor of lurcher protein 1 precursor, putative	*sol-1*	Unclassified, WT
-31	Bm1_19390	Hypothetical protein	*daf-12*	Lon, Unclassified, WT
-32	Bm1_56290	ARID/BRIGHT DNA binding domain containing protein	C01G8.9	Let, Emb, Lvl, Ste, Rup, Bmd, Led, WT, Gro
-32	Bm1_11340	C2-HC type zinc finger protein C.e-MyT1, putative	*ekl-2*	Emb, Unc, WT
-32	Bm1_38435	Conserved hypothetical protein, putative	*tag-60*	Aldicarb resistant, WT
-33	Bm1_03370	LD15209p, putative	C13F10.7	Emb, WT
-33	Bm1_28035	Hypothetical protein	R05D11.7	Egl, Gro, WT
-33	Bm1_23370	Ulp1 protease family, C-terminal catalytic domain containing protein	*ulp-4*	Dpy, aldicarb resistant, Egl, Pch, WT, Gro
-33	Bm1_47300	conserved hypothetical protein	*daf-7*	Aldicarb resistant, WT
-33	Bm1_06640	Protein phosphatase inhibitor containing protein	C07H6.2	Gro, WT
-33	Bm1_33205	Protein cab-1.-related	*cab-1*	Aldicarb resistant, WT
-34	Bm1_27455	Lectin C-type domain containing protein	T19E7.1	Emb, Unclassified, WT
-34	Bm1_22440	Homeobox protein goosecoid, putative	*ceh-45*	Emb, WT

-35	Bm1_52815	conserved hypothetical protein	F45F2.10	Ste, WT, Stp
-36	Bm1_06130	Nuclear anchorage protein 1-related	*anc-1*	Mlt, Lvl, Gro, WT
-36	Bm1_06130	Nuclear anchorage protein 1-related	*anc-1*	Mlt, Lvl, Gro, WT
-37	Bm1_29715	Hypothetical protein	*lin-52*	Lva, Egl, WT, Stp
-37	Bm1_18195	Serine/threonine protein phosphatase PP1 isozyme 1, putative	C24H11.2	Aldicarb resistant, WT
-37	Bm1_37240	ephrin EFN-4, putative	*efn-2*	Unclassified, WT
-38	Bm1_21825	hypothetical protein	W03A5.4	Spindle Elongation/Integrity abnormal, Unclassified, WT
-38	Bm1_56600	cystatin-type cysteine proteinase inhibitor CPI-2, putative	*cli-1*	Ste, WT
-39	Bm1_55355	Protein FAM34A.-related	*acl-14*	Emb, WT
-39	Bm1_21530	hypothetical protein	R11H6.5	Unclassified, WT
-39	Bm1_47345	3 exoribonuclease family, domain 2 containing protein	F37C12.13	Lva, Emb, Larval Arrest-Early (L1/L2), Sterile F1, Ste, WT, Gro
-39	Bm1_38400	Conserved hypothetical protein, putative	Y37D8A.21	Lva, WT
-39	Bm1_13715	Fras1 protein-related	C48E7.6	Emb, WT
-39	Bm1_01555	hypothetical protein	*dgn-1*	Unclassified, WT
-40	Bm1_56045	myosin-like protein, putative	*anc-1*	Mlt, Lvl, Gro, WT
-40	Bm1_17360	conserved hypothetical protein	F25C8.3	Slu, WT
-41	Bm1_11715	Phospholipase c like protein 1, isoform b, putative	*pll-1*	Pnm, Larval Arrest-Late (L3/L4), WT
-42	Bm1_20785	hypothetical protein	*ssl-1*	Emb, WT
-42	Bm1_16705	conserved hypothetical protein	F37C12.2	Lva, Emb, WT
-42	Bm1_02455	WW domain containing protein	F13E6.4	Pvl, WT
-42	Bm1_05345	WD-repeat protein 3.-related	F13H8.2	Larval Arrest-Early (L1/L2), Gro, WT
-43	Bm1_46675	membrane-associated RING-CH protein III, putative	Y57A10B.1	Unclassified, WT
-43	Bm1_43275	membrane-associated RING-CH protein III, putative	C17E4.3	Emb, WT
-43	Bm1_12300	Fibronectin type III domain containing protein	*ptp-3*	Emb, Morphology defect, WT
-45	Bm1_23135	Hypothetical protein	*acy-3*	Unclassified, WT
-46	Bm1_20840	calcium-binding protein, putative	F59D6.7	Unc, WT
-47	Bm1_26685	F25C8.3 protein-related	F25C8.3	Slu, WT
-50	Bm1_54070	F25C8.3 protein-related	F25C8.3	Slu, WT
-51	Bm1_02325	Immunoglobulin I-set domain containing protein	*ketn-1*	Aldicarb resistant, Gro, WT
-52	Bm1_04865	DNA polymerase epsilon p17 subunit, putative	T26A5.8	Pnm, Emb, WT

Previously identified targets are shown with bold identifiers. Manually added annotations are shown in italics. *B. malayi* pub locus and descriptions are from

Ghedin *et al.* [11]. *C. elegans* gene names and RNAi phenotypes are from Wormbase.
