# Supplementary material for: Mining Predicted Essential Genes of Brugia malayi for Nematode Drug Targets
Source: PLoS One. 2007 Nov 14;2(11):e1189. doi: 10.1371/journal.pone.0001189 (PMC2063515; doi:10.1371/journal.pone.0001189)
Supplement: Supplementary Table S2 — Frequency of Interpro domains in the target sequences. (0.09 MB PDF) [file pone.0001189.s002.pdf]

Supplementary Table S2. Frequency of Interpro domains in the target sequences.

Interpro ID	Interpro domain description	Freq
IPR008160	Collagen triple helix repeat	19
IPR002486	Nematode cuticle collagen, N-terminal	17
IPR007087	Zinc finger, C2H2-type	13
IPR000990	Innexin	8
IPR008161	Collagen helix repeat	7
IPR000504	RNA-binding region RNP-1 (RNA recognition motif)	6
IPR001478	PDZ/DHR/GLGF	6
IPR007596	Viral A-type inclusion protein repeat	6
IPR000535	Major sperm protein	5
IPR001092	Basic helix-loop-helix dimerisation region bHLH	5
IPR003656	Zinc finger, BED-type predicted	5
IPR000637	HMG-I and HMG-Y, DNA-binding	4
IPR001841	Zinc finger, RING-type	4
IPR003961	Fibronectin, type III	4
IPR006209	EGF-like	4
IPR007110	Immunoglobulin-like	4
IPR000742	EGF-like, subtype 2	3
IPR001507	Endoglin/CD105 antigen	3
IPR001680	WD-40 repeat	3
IPR002223	Proteinase inhibitor I2, Kunitz metazoa	3
IPR002422	Amino acid/polyamine transporter II	3
IPR004373	Peptide chain release factor 1	3
IPR000210	BTB/POZ	2
IPR000244	Ribosomal protein L9	2
IPR000313	PWWP	2
IPR000357	HEAT	2
IPR000859	CUB	2
IPR001005	Myb, DNA-binding	2
IPR001124	Lipid-binding serum glycoprotein	2
IPR001406	tRNA pseudouridine synthase	2
IPR001606	AT-rich interaction region	2
IPR001611	Leucine-rich repeat	2
IPR001622	K+ channel, pore region	2
IPR001648	Ribosomal protein S18	2
IPR001810	Cyclin-like F-box	2
IPR001876	Zinc finger, RanBP2-type	2
IPR001881	EGF-like calcium-binding	2
IPR001978	Troponin	2
IPR002544	FMRFamide-related peptide	2
IPR002557	Chitin binding Peritrophin-A	2
IPR003014	N/apple PAN	2
IPR003170	UDP-N-acetylenolpyruvoylglucosamine reductase	2
IPR003397	Mitochondrial import inner membrane translocase, subunit Tim17/22	2
IPR003609	Apple-like	2
IPR004307	TspO/MBR-related protein	2

IPR004446	Histidinol phosphatase-related protein	2
IPR005560	Protein of unknown function DUF326	2
IPR006141	Protein splicing (intein) site	2
IPR006149	Nematode-specific EB region	2
IPR007847	Cysteine-rich Deinococcus radiodurans, N-terminal	2
IPR007855	RNA dependent RNA polymerase	2
IPR008191	Maternal tudor protein	2
IPR008572	Protein of unknown function DUF854, Caenorhabditis elegans	2
IPR010625	CHCH	2
IPR010761	Clc-like	2
IPR010811	Protein of unknown function DUF1409	2
IPR011668	Protein of unknown function DUF1610	2
IPR000008	C2	1
IPR000010	Proteinase inhibitor I25, cystatin	1
IPR000058	Zinc finger, AN1-type	1
IPR000082	SEA	1
IPR000198	RhoGAP	1
IPR000221	Protamine P1	1
IPR000225	Armadillo	1
IPR000276	Rhodopsin-like GPCR superfamily	1
IPR000282	Cytokine receptor class 2	1
IPR000342	Regulator of G protein signalling	1
IPR000413	Integrins alpha chain	1
IPR000449	Ubiquitin-associated	1
IPR000456	Ribosomal protein L17	1
IPR000529	Ribosomal protein S6	1
IPR000555	Mov34/MPN/PAD-1	1
IPR000557	Calponin repeat	1
IPR000583	Glutamine amidotransferase, class-II	1
IPR000611	Neuropeptide Y receptor	1
IPR000688	Hydrogenase expression/synthesis, HypA	1
IPR000690	Zinc finger, C2H2-type matrin	1
IPR000712	Apoptosis regulator Bcl-2 protein, BH	1
IPR000716	Thyroglobulin type-1	1
IPR000731	Sterol-sensing 5TM box	1
IPR000737	Proteinase inhibitor I7, squash	1
IPR000837	Fos transforming protein	1
IPR000949	ELM2	1
IPR000953	Chromo	1
IPR000967	Zinc finger, NF-X1-type	1
IPR000998	MAM	1
IPR001036	Acriflavin resistance protein	1
IPR001056	Photosystem II phosphoprotein PsbH	1
IPR001101	Plectin repeat	1
IPR001152	Thymosin beta-4	1
IPR001158	DIX	1
IPR001202	WW/Rsp5/WWP	1
IPR001298	Filamin/ABP280 repeat	1
IPR001304	C-type lectin	1

IPR001313	Pumilio/Puf RNA-binding	1
IPR001356	Homeobox	1
IPR001357	BRCT	1
IPR001394	Peptidase C19, ubiquitin carboxyl-terminal hydrolase 2	1
IPR001395	Aldo/keto reductase	1
IPR001436	Alpha crystallin	1
IPR001440	TPR repeat	1
IPR001469	H+-transporting two-sector ATPase, delta/epsilon subunit	1
IPR001684	Ribosomal protein L27	1
IPR001719	AP endonuclease, family 2	1
IPR001723	Steroid hormone receptor	1
IPR001767	Peptidase C46, hedgehog protein, hint region	1
IPR001770	G-protein, gamma subunit	1
IPR001799	Ephrin	1
IPR001839	Transforming growth factor beta	1
IPR001878	Zinc finger, CCHC-type	1
IPR002035	von Willebrand factor, type A	1
IPR002048	Calcium-binding EF-hand	1
IPR002068	Heat shock protein Hsp20	1
IPR002093	BRCA2 repeat	1
IPR002097	Profilin/allergen	1
IPR002108	Actin-binding, cofilin/tropomyosin type	1
IPR002110	Ankyrin	1
IPR002112	Transcription factor Jun	1
IPR002124	Cytochrome c oxidase, subunit Vb	1
IPR002219	Protein kinase C, phorbol ester/diacylglycerol binding	1
IPR002466	Adenosine deaminase/editase	1
IPR002475	BCL2-like apoptosis inhibitor	1
IPR002515	Zinc finger, C2HC-type	1
IPR002558	I/LWEQ	1
IPR002619	Protein of unknown function CX	1
IPR002637	Ham1-like protein	1
IPR002649	tRNA (guanine-N1-)-methyltransferase	1
IPR002653	Zinc finger, A20-type	1
IPR002673	Ribosomal L29e protein	1
IPR002677	Ribosomal L32p protein	1
IPR002711	HNH endonuclease	1
IPR002790	Protein of unknown function DUF88	1
IPR002850	Protein of unknown function DUF132	1
IPR002876	Protein of unknown function DUF28	1
IPR002885	Pentatricopeptide repeat	1
IPR002918	Lipase, class 2	1
IPR002921	Lipase, class 3	1
IPR002952	Eggshell protein	1
IPR002999	Tudor	1
IPR003093	Apoptosis regulator Bcl-2 protein, BH4	1
IPR003107	RNA-processing protein, HAT helix	1
IPR003109	GoLoco	1
IPR003117	cAMP-dependent protein kinase regulator, type II PKA R	1

	subunit	
IPR003121	SWIB/MDM2	1
IPR003124	Actin-binding WH2	1
IPR003169	GYF	1
IPR003213	Cytochrome oxidase c, subunit VIb	1
IPR003243	Proteinase inhibitor I25A and I25B, type 2 and phytocystatins	1
IPR003267	Small proline-rich	1
IPR003323	Ovarian tumour, otubain	1
IPR003335	SecD/SecF/SecDF export membrane protein	1
IPR003341	Protein of unknown function DUF139, cysteine rich	1
IPR003345	M protein repeat	1
IPR003367	Thrombospondin type 3 repeat	1
IPR003392	Patched	1
IPR003422	Ubiquinol-cytochrome C reductase hinge protein	1
IPR003428	Mitochondrial glycoprotein	1
IPR003582	Metridin-like ShK toxin	1
IPR003591	Leucine-rich repeat, typical subtype	1
IPR003639	Mov34-1	1
IPR003653	Peptidase C48, SUMO/Sentrin/Ubl1	1
IPR003683	Cytochrome b6/f complex, subunit 5	1
IPR003822	Paired amphipathic helix	1
IPR003882	Pistil-specific extensin-like protein	1
IPR003894	TAFH/NHR1	1
IPR003900	RepA / Rep+ protein KID	1
IPR003958	Transcription factor CBF/NF-Y/archaeal histone	1
IPR004012	RUN	1
IPR004122	Barrier to autointegration factor, BAF	1
IPR004146	DC1	1
IPR004149	Zinc-finger, NAD-dependent DNA ligase C4-type	1
IPR004153	CXCXC repeat	1
IPR004168	PPAK motif	1
IPR004203	Cytochrome c oxidase subunit IV	1
IPR004205	UcrQ	1
IPR004245	Protein of unknown function DUF229	1
IPR004365	nucleic acid binding, OB-fold, tRNA/helicase-type	1
IPR004374	Peptide chain release factor 2	1
IPR004417	Gid protein	1
IPR004418	Homoaconitase	1
IPR004821	Cytidyltransferase-related	1
IPR004827	Basic-leucine zipper (bZIP) transcription factor	1
IPR004830	Leucine rich repeat variant	1
IPR005024	Snf7	1
IPR005132	Rare lipoprotein A	1
IPR005194	Glycoside hydrolase, family 65, C-terminal	1
IPR005229	Conserved hypothetical protein 255	1
IPR005289	GTP-binding	1
IPR005290	Ribosomal protein S15, bacterial chloroplast and mitochondrial type	1
IPR005366	Protein of unknown function UPF0172	1

IPR005455	Plant profilin	1
IPR005716	Ribosomal protein S7, eukaryotic and archaeal form	1
IPR005735	Zinc finger, LSD1-type	1
IPR005804	Fatty acid desaturase	1
IPR006121	Heavy metal transport/detoxification protein	1
IPR006124	Metalloenzyme	1
IPR006195	Aminoacyl-transfer RNA synthetase, class II	1
IPR006219	Phospho-2-dehydro-3-deoxyheptonate aldolase, subtype 1	1
IPR006343	DnaD and phage-associated region	1
IPR006387	Plasmodium falciparum CPW-WPC	1
IPR006450	Uncharacterized phage protein (possible DNA packaging)	1
IPR006456	ZF-HD homeobox protein Cys/His-rich dimerisation region	1
IPR006487	Phage minor tail protein L	1
IPR006634	TRAM, LAG1 and CLN8 homology	1
IPR006649	Like-Sm ribonucleoprotein, eukaryotic and archaea-type, core	1
IPR006652	Kelch repeat	1
IPR006695	CENP-B, N-terminal DNA-binding	1
IPR006718	Dec-1 repeat	1
IPR006721	Mitochondrial ATP synthase epsilon chain	1
IPR006770	Opioid growth factor receptor repeat	1
IPR006785	Peroxisomal membrane anchor protein (Pex14p)	1
IPR006808	Mitochondrial ATP synthase g subunit	1
IPR006861	Hyaluronan/mRNA binding protein	1
IPR006954	Protein of unknown function DUF644	1
IPR007019	Surfeit locus 6	1
IPR007089	Leucine-rich repeat, cysteine-containing	1
IPR007092	Leucine-rich repeat, SDS22+-like	1
IPR007124	Histone-fold/TFIID-TAF/NF-Y	1
IPR007125	Histone core	1
IPR007129	Ubiquinol-cytochrome C chaperone	1
IPR007274	Ctr copper transporter	1
IPR007284	Protein of unknown function DUF398, Ground-like region	1
IPR007308	Protein of unknown function DUF408	1
IPR007330	MIT	1
IPR007529	Zinc finger, HIT-type	1
IPR007588	Zinc finger, FLYWCH-type	1
IPR007730	Sporulation related	1
IPR007741	Mitochondrial ribosome	1
IPR007811	RNA polymerase III RPC4	1
IPR007826	Photosystem II protein PsbM	1
IPR007829	TM2	1
IPR007834	DSS1/SEM1	1
IPR007858	Dpy-30	1
IPR007866	Protein of unknown function DUF714	1
IPR007900	Transcription initiation factor TFIID component TAF4	1
IPR007945	Neuroendocrine 7B2 precursor	1
IPR007967	Protein of unknown function DUF727	1
IPR007992	CybS	1
IPR008080	Parvalbumin	1

IPR008166	Protein of unknown function DUF23	1
IPR008197	Whey acidic protein, core region	1
IPR008386	ATP synthase E	1
IPR008387	Mitochondrial ATP synthase coupling factor 6	1
IPR008389	ATP synthase subunit H	1
IPR008448	Chordopoxvirus DNA-directed RNA polymerase 7 kDa polypeptide	1
IPR008504	Protein of unknown function DUF786	1
IPR008569	Protein of unknown function DUF851, Caenorhabditis elegans	1
IPR008593	DNA N-6-adenine-methyltransferase	1
IPR008597	Destabilase	1
IPR008669	Lsm interaction	1
IPR008688	Mitochondrial ATP synthase B chain	1
IPR008689	ATP synthase D chain, mitochondrial	1
IPR008699	NADH-ubiquinone oxidoreductase ASHI subunit	1
IPR008850	TEP1, N-terminal	1
IPR008906	HAT dimerisation	1
IPR009149	BR22	1
IPR009332	Surfeit locus 5	1
IPR009346	GRIM-19	1
IPR009423	NADH-ubiquinone oxidoreductase subunit b14.5b	1
IPR009578	Streptococcal surface antigen	1
IPR009719	Protein of unknown function DUF1296	1
IPR009762	Protein of unknown function DUF1331	1
IPR009805	Variable length PCR target	1
IPR009851	Modifier of rudimentary, Modr	1
IPR010010	Photosystem I M	1
IPR010012	Spasmodic peptide gm9a	1
IPR010257	Fatty acid desaturase subdomain	1
IPR010492	GINS complex, Psf3 component	1
IPR010514	COX aromatic rich	1
IPR010663	Zinc finger, Fpg-type	1
IPR011107	Protein phosphatase inhibitor	1
IPR011120	Neutral trehalase Ca2+ binding	1
IPR011424	C1-like	1
IPR011461	Protein of unknown function DUF1567	1
IPR011466	Protein of unknown function DUF1572	1
IPR011490	Uncharacterised sugar-binding	1
IPR011498	Kelch	1
IPR011506	Planctomycete extracellular	1
IPR011515	Shugoshin, C-terminal	1
IPR011521	YTV	1
IPR011562	Cofactor-independent phosphoglycerate mutase / phosphopentomutase	1
IPR011616	bZIP transcription factor, bZIP_1	1
IPR011633	Protein of unknown function DUF1602	1
IPR011647	KWG Leptospira	1
IPR011700	Basic leucine zipper	1
IPR011717	Tetratricopeptide TPR_4	1
