# Supplementary material for: Mining Predicted Essential Genes of Brugia malayi for Nematode Drug Targets
Source: PLoS One. 2007 Nov 14;2(11):e1189. doi: 10.1371/journal.pone.0001189 (PMC2063515; doi:10.1371/journal.pone.0001189)
Supplement: Supplementary Table S3 — EC numbers mapped to targets using ec2go. (0.05 MB PDF) [file pone.0001189.s003.pdf]

Supplementary Table S3. EC numbers mapped to targets using ec2go.

B. malayi pub locus	GO ID	EC ID	EC Description
Bm1_56530	GO:0006412	EC:3.6.5.3	protein-synthesizing GTPase
Bm1_20745	GO:0004000	EC:3.5.4.4	adenosine deaminase
Bm1_24615	GO:0046933	EC:3.6.3.14	H+-transporting two-sector ATPase
Bm1_24615	GO:0046961	EC:3.6.3.14	H+-transporting two-sector ATPase
Bm1_00120	GO:0046933	EC:3.6.3.14	H+-transporting two-sector ATPase
Bm1_00120	GO:0046961	EC:3.6.3.14	H+-transporting two-sector ATPase
Bm1_35215	GO:0016758	EC:2.4.1	Glycosyltransferases
Bm1_17930	GO:0016758	EC:2.4.1	Glycosyltransferases
Bm1_18685	GO:0008137	EC:1.6.5.3	NADH dehydrogenase (ubiquinone)
Bm1_18685	GO:0004129	EC:1.9.3.1	cytochrome-c oxidase
Bm1_03920	GO:0004129	EC:1.9.3.1	cytochrome-c oxidase
Bm1_03920	GO:0004129	EC:1.9.3.1	cytochrome-c oxidase
Bm1_38160	GO:0016491	EC:1	Oxidoreductases
Bm1_37810	GO:0005834	EC:3.6.5.1	heterotrimeric G-protein GTPase
Bm1_16530	GO:0004553	EC:3.2.1	phosphoric monoester hydrolases
Bm1_16555	GO:0046933	EC:3.6.3.14	H+-transporting two-sector ATPase
Bm1_16555	GO:0046961	EC:3.6.3.14	H+-transporting two-sector ATPase
Bm1_52630	GO:0004806	EC:3.1.1.3	triacylglycerol lipase
Bm1_14330	GO:0046933	EC:3.6.3.14	H+-transporting two-sector ATPase
Bm1_14330	GO:0046961	EC:3.6.3.14	H+-transporting two-sector ATPase
Bm1_38880	GO:0046933	EC:3.6.3.14	H+-transporting two-sector ATPase
Bm1_38880	GO:0046961	EC:3.6.3.14	H+-transporting two-sector ATPase
Bm1_44000	GO:0003954	EC:1.6.99.3	NADH dehydrogenase
Bm1_44000	GO:0008137	EC:1.6.5.3	NADH dehydrogenase (ubiquinone)
Bm1_23180	GO:0006412	EC:3.6.5.3	protein-synthesizing GTPase
Bm1_02135	GO:0006412	EC:3.6.5.3	protein-synthesizing GTPase
Bm1_14750	GO:0008121	EC:1.10.2.2	ubiquinol—cytochrome-c reductase
Bm1_29880	GO:0004197	EC:3.4.22	Cysteine endopeptidases
Bm1_29880	GO:0004221	EC:3.1.2.15	ubiquitin thiolesterase
Bm1_23555	GO:0008121	EC:1.10.2.2	ubiquinol—cytochrome-c reductase
Bm1_17810	GO:0046933	EC:3.6.3.14	H+-transporting two-sector ATPase
Bm1_17810	GO:0046961	EC:3.6.3.14	H+-transporting two-sector ATPase
