# Supplementary material for: Mining Predicted Essential Genes of Brugia malayi for Nematode Drug Targets
Source: PLoS One. 2007 Nov 14;2(11):e1189. doi: 10.1371/journal.pone.0001189 (PMC2063515; doi:10.1371/journal.pone.0001189)
Supplement: Supplementary Table S4 — GO terms associated with target pool sequences. The GO terms are a subset of the GO hierarchy (GO slim). All children of the GO slim nodes are mapped up to the nearest parent in the slim hierarchy. Counts total the occurrences of the exact GO term listed and all its children. (0.07 MB PDF) [file pone.0001189.s004.pdf]

Supplementary Table S4. GO terms associated with target pool sequences.

GO Category/Term	All Genes		Target Pool	
**Biological Process**	**count**	**%**	**count**	**%**
GO:0008150 biological_process	2572	33.1%	75	33.8%
GO:0008152 metabolism	1817	23.4%	37	16.7%
GO:0006810 transport	443	5.7%	33	14.9%
GO:0006811 ion transport	233	3.0%	30	13.5%
GO:0050794 regulation of cellular process	314	4.0%	11	5.0%
GO:0006350 transcription	300	3.9%	9	4.1%
GO:0019538 protein metabolism	798	10.3%	6	2.7%
GO:0007154 cell communication	239	3.1%	6	2.7%
GO:0007165 signal transduction	235	3.0%	5	2.3%
GO:0006629 lipid metabolism	71	0.9%	2	0.9%
GO:0015031 protein transport	68	0.9%	2	0.9%
GO:0007049 cell cycle	45	0.6%	2	0.9%
GO:0006259 DNA metabolism	117	1.5%	1	0.5%
GO:0007275 multicellular organismal development	28	0.4%	1	0.5%
GO:0030154 cell differentiation	20	0.3%	1	0.5%
GO:0016265 death	15	0.2%	1	0.5%
GO:0006464 protein modification	375	4.8%	0	0.0%
GO:0050896 response to stimulus	52	0.7%	0	0.0%
GO:0006950 response to stress	36	0.5%	0	0.0%
**Cellular Component**	**count**	**%**	**count**	**%**
GO:0005575 cellular_component	1691	25.5%	90	24.3%
GO:0005623 cell	1640	24.8%	88	23.7%
GO:0005622 intracellular	1147	17.3%	65	17.5%
GO:0043226 organelle	854	12.9%	41	11.1%
GO:0005737 cytoplasm	349	5.3%	35	9.4%
GO:0005634 nucleus	582	8.8%	24	6.5%
GO:0005739 mitochondrion	31	0.5%	10	2.7%
GO:0005886 plasma membrane	36	0.5%	8	2.2%
GO:0005840 ribosome	101	1.5%	3	0.8%
GO:0005856 cytoskeleton	59	0.9%	2	0.5%
GO:0005777 peroxisome	3	0.0%	2	0.5%
GO:0016023 cytoplasmic membrane-bound vesicle	23	0.3%	1	0.3%
GO:0005654 nucleoplasm	20	0.3%	1	0.3%
GO:0005815 microtubule organizing center	2	0.0%	1	0.3%
GO:0005783 endoplasmic reticulum	25	0.4%	0	0.0%
GO:0005730 nucleolus	23	0.3%	0	0.0%
GO:0005794 Golgi apparatus	19	0.3%	0	0.0%
GO:0005829 cytosol	12	0.2%	0	0.0%
GO:0005773 vacuole	2	0.0%	0	0.0%
GO:0005764 lysosome	1	0.0%	0	0.0%
**Molecular Function**	**count**	**%**	**count**	**%**
GO:0003674 molecular_function	3348	27.4%	112	34.1%
GO:0005488 binding	1998	16.3%	49	14.9%
GO:0003676 nucleic acid binding	882	7.2%	25	7.6%
GO:0003824 catalytic activity	1447	11.8%	24	7.3%
GO:0005198 structural molecule activity	192	1.6%	24	7.3%
GO:0005215 transporter activity	221	1.8%	16	4.9%
GO:0016787 hydrolase activity	518	4.2%	13	4.0%
GO:0005515 protein binding	284	2.3%	12	3.7%
GO:0003677 DNA binding	356	2.9%	9	2.7%

GO:0030528 transcription regulator activity	224	1.8%	8	2.4%
GO:0030234 enzyme regulator activity	62	0.5%	5	1.5%
GO:0004871 signal transducer activity	140	1.1%	4	1.2%
GO:0008233 peptidase activity (peptidase activity)	175	1.4%	3	0.9%
GO:0003700 transcription factor activity	138	1.1%	3	0.9%
GO:0004872 receptor activity	108	0.9%	3	0.9%
GO:0030246 carbohydrate binding	22	0.2%	3	0.9%
GO:0016740 transferase activity	460	3.8%	2	0.6%
GO:0003723 RNA binding	80	0.7%	2	0.6%
GO:0008289 lipid binding	30	0.2%	2	0.6%
GO:0008092 cytoskeletal protein binding	23	0.2%	2	0.6%
GO:0003779 actin binding	16	0.1%	2	0.6%
GO:0009055 electron carrier activity	9	0.1%	2	0.6%
GO:0005509 calcium ion binding	78	0.6%	1	0.3%
GO:0004518 nuclease activity	41	0.3%	1	0.3%
GO:0003682 chromatin binding	10	0.1%	1	0.3%
GO:0000166 nucleotide binding	659	5.4%	0	0.0%
GO:0016301 kinase activity	268	2.2%	0	0.0%
GO:0004672 protein kinase activity	222	1.8%	0	0.0%
GO:0005216 ion channel activity	65	0.5%	0	0.0%
GO:0004721 phosphoprotein phosphatase activity	45	0.4%	0	0.0%
GO:0003774 motor activity	33	0.3%	0	0.0%
GO:0008135 translation factor activity, nucleic acid binding	23	0.2%	0	0.0%
GO:0045182 translation regulator activity	23	0.2%	0	0.0%
GO:0005102 receptor binding	16	0.1%	0	0.0%
GO:0016209 antioxidant activity	9	0.1%	0	0.0%
GO:0005326 neurotransmitter transporter activity	4	0.0%	0	0.0%

The GO terms are a subset of the GO hierarchy (GO slim). All children of the GO slim nodes are mapped up to the nearest parent in the slim hierarchy. Counts total the occurrences of the exact GO term listed and all its children.
