# Supplementary material for: Mining Predicted Essential Genes of Brugia malayi for Nematode Drug Targets
Source: PLoS One. 2007 Nov 14;2(11):e1189. doi: 10.1371/journal.pone.0001189 (PMC2063515; doi:10.1371/journal.pone.0001189)
Supplement: Supplementary Table S5 — RNAi phenotype components of each binning category. (0.12 MB PDF) [file pone.0001189.s005.pdf]

Supplementary Table S5. RNAi phenotype components of each binning category.

RNAi Phenotype	Binning Category
Aberrant Cytoplasmic Structures	Embryonic Lethal/Arrest
Abs	Embryonic Lethal/Arrest
Asymmetry of Division abnormal	Embryonic Lethal/Arrest
Centrosome Attachment abnormal	Embryonic Lethal/Arrest
Chromosome Segregation (karyomeres) abnormal	Embryonic Lethal/Arrest
Complex Phenotype	Embryonic Lethal/Arrest
Cortical Dynamics abnormal	Embryonic Lethal/Arrest
Cpa	Embryonic Lethal/Arrest
Cyk	Embryonic Lethal/Arrest
Emb	Embryonic Lethal/Arrest
Etv	Embryonic Lethal/Arrest
Integrity of Membranous Organelles defective	Embryonic Lethal/Arrest
Led	Embryonic Lethal/Arrest
Let	Embryonic Lethal/Arrest
Lethal	Embryonic Lethal/Arrest
Mul	Embryonic Lethal/Arrest
Nmo	Embryonic Lethal/Arrest
Npo	Embryonic Lethal/Arrest
Nuclear Appearance abnormal	Embryonic Lethal/Arrest
Ocs	Embryonic Lethal/Arrest
Oma	Embryonic Lethal/Arrest
Osmotic Integrity defective	Embryonic Lethal/Arrest
Oth	Embryonic Lethal/Arrest
Passage Through Meiosis defective	Embryonic Lethal/Arrest
Pat	Embryonic Lethal/Arrest
Pna	Embryonic Lethal/Arrest
Pnm	Embryonic Lethal/Arrest
Pronuclear/Nuclear Appearance abnormal	Embryonic Lethal/Arrest
Rot	Embryonic Lethal/Arrest
Severe Pleiotropic Defects	Embryonic Lethal/Arrest
Sister Chromatid Separation abnormal (Cross-eyed)	Embryonic Lethal/Arrest
Sle	Embryonic Lethal/Arrest
Spd	Embryonic Lethal/Arrest
Spindle Assembly abnormal	Embryonic Lethal/Arrest
Spindle Elongation/Integrity abnormal	Embryonic Lethal/Arrest
Spn	Embryonic Lethal/Arrest
Spo	Embryonic Lethal/Arrest
Age	Growth Defect
Daf	Growth Defect

Developmental Delay	Growth Defect
General Pace of Development abnormal	Growth Defect
Gro	Growth Defect
Mab	Growth Defect
Mlt	Growth Defect
Sck	Growth Defect
Adl	Larval/Adult Lethal/Arrest
Larval Arrest-Early (L1/L2)	Larval/Adult Lethal/Arrest
Larval Arrest-Late (L3/L4)	Larval/Adult Lethal/Arrest
Larval Lethal-Early (L1/L2)	Larval/Adult Lethal/Arrest
Larval Lethal-Late (L3/L4)	Larval/Adult Lethal/Arrest
Lva	Larval/Adult Lethal/Arrest
Lvl	Larval/Adult Lethal/Arrest
Bli	Morphology Defect
Bmd	Morphology Defect
Clr	Morphology Defect
Dpy	Morphology Defect
Lon	Morphology Defect
Morphology defect	Morphology Defect
Pch	Morphology Defect
Rol	Morphology Defect
Rup	Morphology Defect
Sma	Morphology Defect
Hya	Movement Defect
Prl	Movement Defect
Prz	Movement Defect
Slu	Movement Defect
Unc	Movement Defect
Ced	Other
Esp	Other
Fem	Other
Fog	Other
Him	Other
Lin	Other
Lpd	Other
Mec	Other
Mut	Other
Rde	Other
Unclassified	Other
Abnormal coloration	Other
aldicarb_resistant	Other

Egg Size abnormal	Other
Entry Into Interphase abnormal	Other
Pace of P-Lineage abnormal	Other
Pro	Other
Stress Resistance	Other
Fgc	Sterility
Glp	Sterility
Gon	Sterility
Lag	Sterility
Mei	Sterility
Mel	Sterility
Mig	Sterility
Ooc	Sterility
Reproductive defect	Sterility
Ste	Sterility
Sterile F0/Fertility Problems	Sterility
Sterile F1	Sterility
Sterility/Impaired Fertility in F0	Sterility
Stp	Sterility
Tum	Sterility
Abnormal coloration	Unclassified
aldicarb_resistant	Unclassified
Egg Size abnormal	Unclassified
Entry Into Interphase abnormal	Unclassified
Pace of P-Lineage abnormal	Unclassified
Pro	Unclassified
Stress Resistance	Unclassified
Egl	Vulval/Egg Laying Defect
Evl	Vulval/Egg Laying Defect
Muv	Vulval/Egg Laying Defect
Pvl	Vulval/Egg Laying Defect
Pvu	Vulval/Egg Laying Defect
Vul	Vulval/Egg Laying Defect
