# Supplementary material for: Mining Predicted Essential Genes of Brugia malayi for Nematode Drug Targets
Source: PLoS One. 2007 Nov 14;2(11):e1189. doi: 10.1371/journal.pone.0001189 (PMC2063515; doi:10.1371/journal.pone.0001189)
Supplement: Supplementary Data Set S1 — Data set for target prioritization. Data values used in assigning scores for prioritization of targets. Maximum bit scores for alignments of putative B. malayi, C. elegans and Human orthologs were obtained from BLASTP results (see materials and methods). Bitscores of 0.0 are recorded when no similarity was identified with an E-value below the threshold used in the BLAST comparison. The total number of RNAi experiments reported for each target gene are based on wormbase release 150. Pheno Bins record the number of instances that a phenotype was reported in these experiments that belongs to each of 9 phenotype bins (see text). In this table, the “other/unclassified” bin was split into “other” and “unclassified” bins. Stage expression count refers to the number of distinct life cycle stages (L2, L3, L4, adult and microfilariae) having EST evidence for a particular target gene. L2, L3, L4, adult and microfilariae record the number of ESTs for that stage. Total Score was calculated as described in Table 2. Known targets are indicated in bold. Na indicates “no value”. (0.13 MB PDF) [file pone.0001189.s006.pdf]

Data Set S1

B. malayi pub locus	C. elegans max bitscore	B. malayi length/C. elegans length	human max bitscore	Total RNAi experiments	Pheno Bin larval/adult lethality/arre	Pheno Bin embryonic lethality	Pheno Bin sterility	Pheno Bin morphology defect	Pheno Bin growth defect	Pheno Bin movement defect	Pheno Bin vulval/egg laying defect	Pheno Bin other	Pheno Bin WT	Pheno Bin unclassified	Stage expression count	L2	L3	L4	Adult	microfilariae	LR5_descrip/druggable_EC #	GRAVY hydropathicity	TOTAL_SCORE
**Bm1_35120**	1141.0	0.99	0.0	4	2	1	0	1	0	1	3	0	1	0	1	0	0	0	1	0	na	-0.19	275
**Bm1_36170**	1107.0	0.90	0.0	5	5	0	1	1	1	0	4	0	1	0	1	0	0	0	2	0	na	-0.29	248
Bm1_45135	98.6	0.74	0.0	1	1	0	0	1	0	1	0	0	0	0	1	0	1	0	0	0	na	-0.43	248
**Bm1_35215**	1123.0	0.98	75.9	5	3	1	0	0	0	3	0	0	1	0	0	0	0	0	0	0	na	0.19	179
Bm1_36850	132.0	1.04	0.0	1	0	0	1	1	0	0	0	0	0	0	1	0	0	0	1	0	na	-0.21	172
**Bm1_22725**	861.0	0.90	0.0	5	4	1	0	0	0	0	0	0	3	0	0	0	0	0	0	0	na	-0.32	172
Bm1_15245	197.0	0.96	58.9	1	1	1	0	0	0	0	0	0	0	0	3	0	1	1	1	0	na	-0.30	157
Bm1_43465	702.0	1.07	0.0	4	1	0	2	1	0	0	0	0	3	0	0	0	0	0	0	0	na	0.17	157
Bm1_38120	620.0	0.97	0.0	4	0	1	0	3	0	0	3	0	1	0	0	0	0	0	0	0	na	-0.26	154
Bm1_35395	697.0	0.93	0.0	3	0	0	0	2	2	0	0	0	1	0	0	0	0	0	0	0	na	0.17	151
Bm1_36765	666.0	1.10	0.0	4	0	1	0	0	0	1	0	0	3	0	0	0	0	0	0	0	na	-0.39	143
Bm1_25640	684.0	0.72	0.0	3	0	2	1	0	0	2	0	0	1	0	0	0	0	0	0	0	na	-0.52	141
**Bm1_35480**	394.0	1.24	0.0	3	0	0	1	3	0	0	3	0	1	0	0	0	0	0	0	0	na	-0.20	140
Bm1_49915	232.0	0.94	0.0	1	1	0	0	0	0	0	0	0	1	0	0	0	0	0	0	0	na	-0.69	133
Bm1_45670	100.0	2.28	48.5	1	1	0	0	0	0	0	0	0	1	0	1	0	0	0	0	1	Integrins; alpha-chain	-0.70	125
**Bm1_37495**	489.0	0.84	0.0	4	0	4	0	3	0	0	2	1	1	0	0	0	0	0	0	0	na	-0.17	123
Bm1_46940	252.0	1.04	0.0	3	2	0	2	1	1	0	1	0	1	0	1	0	0	0	1	0	na	-0.50	119
Bm1_38110	481.0	0.95	0.0	4	0	0	1	2	0	0	2	0	2	0	0	0	0	0	0	0	na	-0.26	116
Bm1_32730	511.0	1.07	55.1	5	0	0	2	1	0	0	0	0	3	0	0	0	0	0	0	0	glycoprotein	-0.03	115
Bm1_42470	270.0	1.01	0.0	4	4	0	1	0	0	0	4	1	1	0	1	0	0	0	1	0	na	-0.50	114
Bm1_55705	166.0	0.66	0.0	1	1	0	0	0	0	0	0	0	1	0	0	0	0	0	0	0	na	-0.37	112
Bm1_38105	516.0	1.11	0.0	6	0	0	0	2	0	0	0	0	4	0	1	0	0	1	0	0	na	-0.18	110
Bm1_38425	736.0	1.00	61.2	5	0	1	1	2	0	0	0	0	3	0	0	0	0	0	0	0	na	-0.21	108
Bm1_43740	478.0	0.90	0.0	3	0	0	0	2	2	0	0	0	1	0	0	0	0	0	0	0	na	0.18	107
Bm1_19285	808.0	0.90	47.4	6	0	0	0	0	0	0	0	2	4	0	0	0	0	0	0	0	na	-0.26	107
Bm1_51995	220.0	0.87	0.0	3	2	0	0	0	0	0	0	0	2	0	0	0	0	0	0	0	glycoprotein	0.13	106
**Bm1_38160**	442.0	1.00	0.0	4	2	0	0	0	0	0	0	1	2	0	3	0	4	0	2	2	na	0.05	105
Bm1_02135	109.0	0.39	0.0	1	1	0	0	0	0	0	0	0	1	0	2	0	1	0	2	0	na	-0.28	105

Bm1_03880	67.4	1.74	0.0	1	1	0	0	0	0	0	0	0	1	0	0	0	0	0	0	0	na	-0.62	103
Bm1_35075	450.0	1.01	0.0	5	0	1	0	2	0	0	1	0	3	0	0	0	0	0	0	0	na	-0.01	100
Bm1_31660	382.0	1.01	0.0	5	6	0	0	0	0	1	0	0	0	0	0	0	0	0	0	0	na	0.13	99
**Bm1_02195**	250.0	0.60	0.0	3	0	0	1	3	0	0	3	0	1	0	2	0	0	0	3	1	na	-0.14	98
Bm1_09270	315.0	0.96	0.0	4	0	1	2	2	2	1	0	0	2	0	2	0	1	0	0	1	na	-0.45	98
Bm1_50630	423.0	0.91	0.0	4	0	1	1	3	0	0	0	0	2	0	0	0	0	0	0	0	na	-0.32	97
**Bm1_08695**	422.0	1.05	0.0	5	0	2	0	1	0	0	1	0	3	0	0	0	0	0	0	0	na	-0.36	96
Bm1_39265	234.0	0.99	0.0	2	1	0	0	0	0	0	1	1	1	0	0	0	0	0	0	0	na	-0.38	96
**Bm1_34455**	468.0	0.86	0.0	8	0	0	5	3	0	0	1	2	3	0	0	0	0	0	0	0	na	-0.42	91
Bm1_08915	471.0	0.93	0.0	4	0	0	0	0	0	0	0	1	3	0	0	0	0	0	0	0	na	-0.32	88
Bm1_16245	246.0	1.06	0.0	4	3	1	0	0	0	0	1	1	1	0	2	0	1	0	2	0	na	-0.87	84
Bm1_33575	238.0	0.92	0.0	4	1	1	0	0	0	0	5	0	1	0	0	0	0	0	0	0	na	0.22	82
Bm1_39790	388.0	1.06	0.0	4	0	1	0	0	0	0	0	1	3	0	0	0	0	0	0	0	na	-0.24	82
Bm1_33440	315.0	1.08	0.0	5	0	1	2	2	0	1	0	0	3	0	1	0	1	0	0	0	na	0.11	81
Bm1_41495	286.0	1.14	0.0	8	8	1	0	0	2	2	0	0	3	0	1	0	0	0	1	0	na	-0.88	81
Bm1_55030	74.7	1.41	0.0	2	1	1	0	0	0	1	0	0	1	0	2	0	1	0	1	0	na	0.19	79
Bm1_07680	564.0	0.98	70.5	4	0	1	0	3	0	0	1	1	1	0	1	0	1	0	0	0	na	-0.29	79
Bm1_30085	105.0	1.70	0.0	5	2	0	0	0	0	0	0	0	3	0	0	0	0	0	0	0	Apoptosis regulator protein, BCL2-family BH domain	-0.18	77
Bm1_56645	201.0	1.04	0.0	4	2	0	1	0	0	0	2	1	1	0	2	0	3	0	2	0	na	-0.62	77
Bm1_49050	110.0	1.08	0.0	4	0	2	1	5	0	0	3	0	1	0	1	0	0	0	2	0	na	0.70	75
Bm1_49335	358.0	0.98	0.0	5	0	0	0	2	0	0	0	0	3	0	0	0	0	0	0	0	na	-0.37	75
Bm1_05470	250.0	0.96	0.0	4	3	0	0	0	0	1	0	0	1	0	1	0	0	0	2	0	na	0.19	74
Bm1_53115	116.0	0.26	0.0	2	0	1	0	0	0	0	0	1	1	0	0	0	0	0	0	0	TspO- and MBR-related proteins	0.00	74
Bm1_41565	363.0	1.33	0.0	7	1	0	0	0	0	0	0	0	6	0	0	0	0	0	0	0	na	-0.27	74
Bm1_56145	154.0	0.83	0.0	5	1	5	2	1	0	2	2	0	1	0	2	0	0	0	4	1	na	-0.55	73
**Bm1_51960**	313.0	1.05	0.0	8	0	0	5	3	0	0	1	2	3	0	0	0	0	0	0	0	na	-0.14	72
Bm1_44220	72.8	0.78	0.0	3	2	0	1	2	0	0	1	0	1	0	3	0	1	0	1	1	na	-0.63	72
Bm1_05960	770.0	0.85	75.1	7	0	0	0	1	0	0	0	0	6	0	0	0	0	0	0	0	na	-0.14	71
Bm1_24555	64.7	0.83	0.0	3	1	1	1	2	2	1	0	0	1	0	0	0	0	0	0	0	na	-0.44	71
Bm1_16555	112.0	1.00	0.0	4	3	3	0	0	0	1	1	0	0	0	3	0	1	1	2	0	na	-0.84	71
Bm1_35415	191.0	0.98	0.0	2	1	0	0	0	0	0	0	1	1	0	2	0	4	0	3	0	na	-0.61	70
Bm1_52630	99.8	2.02	0.0	5	0	0	0	0	0	0	0	0	4	1	0	0	0	0	0	0	EC:3.1.1.3	-0.20	69
Bm1_21610	276.0	1.00	0.0	6	0	0	0	0	1	1	1	0	5	0	1	0	0	0	1	0	na	-0.25	68
Bm1_45560	297.0	1.04	0.0	4	0	0	0	0	1	1	0	0	3	0	0	0	0	0	0	0	na	-0.46	68
Bm1_14680	274.0	1.00	0.0	6	0	0	0	0	1	1	1	0	5	0	1	0	0	0	1	0	na	-0.22	67
Bm1_14330	62.0	1.31	0.0	4	2	2	0	0	0	3	2	0	0	0	4	1	2	0	2	1	na	-0.69	67
Bm1_47700	122.0	1.17	50.1	2	2	0	0	0	1	1	0	0	0	0	1	0	0	0	1	0	na	-0.01	67
Bm1_07635	113.0	1.03	0.0	3	3	0	0	0	0	1	1	0	0	0	0	0	0	0	0	0	na	0.75	67
Bm1_53755	139.0	1.00	0.0	3	2	1	0	0	0	0	1	0	1	0	0	0	0	0	0	0	na	-0.52	67
Bm1_45175	102.0	1.05	52.8	1	1	0	0	0	0	0	0	0	1	0	0	0	0	0	0	0	na	-0.10	67
Bm1_37530	175.0	0.94	0.0	5	0	1	2	2	3	2	0	0	2	0	1	0	0	0	1	0	na	-0.58	66
Bm1_33125	149.0	0.28	0.0	3	0	1	1	0	0	0	3	0	0	0	1	0	0	0	1	0	na	-0.34	65

Bm1_21620	157.0	0.98	0.0	5	7	0	0	1	0	1	0	0	0	0	1	0	0	0	0	1	na	-0.25	65
Bm1_18695	87.8	1.00	0.0	3	0	0	3	2	0	0	0	0	0	0	1	0	0	0	2	0	na	-0.78	65
Bm1_38065	334.0	0.92	0.0	6	0	0	1	1	1	0	0	0	5	0	0	0	0	0	0	0	na	0.08	64
Bm1_25750	332.0	0.99	0.0	3	0	0	0	0	0	0	0	1	2	0	0	0	0	0	0	0	na	0.06	64
Bm1_51095	596.0	0.89	49.7	7	0	0	0	0	0	0	0	0	6	1	0	0	0	0	0	0	na	-0.02	64
Bm1_51050	85.1	0.96	0.0	4	3	2	0	0	0	0	2	0	0	0	5	1	2	2	2	1	na	-0.22	64
Bm1_44775	516.0	0.55	0.0	15	14	1	0	0	1	3	0	0	3	0	0	0	0	0	0	0	na	-0.30	64
Bm1_32310	267.0	1.16	0.0	5	0	1	0	2	0	0	1	0	3	0	0	0	0	0	0	0	na	-0.10	63
Bm1_13015	103.0	1.02	0.0	3	0	0	4	0	0	0	0	0	0	0	3	0	1	1	8	0	na	-0.64	63
Bm1_44070	136.0	1.03	0.0	4	3	1	1	0	1	0	1	0	2	0	0	0	0	0	0	0	na	-0.52	63
Bm1_46095	53.5	1.25	0.0	2	2	0	0	0	0	0	0	0	1	0	2	0	1	0	1	0	na	-0.40	63
Bm1_08035	229.0	0.97	0.0	7	3	1	0	0	0	0	6	0	0	0	0	0	0	0	0	0	na	-0.17	63
Bm1_56805	147.0	1.03	0.0	4	2	1	1	0	0	0	2	1	1	0	0	0	0	0	0	0	na	-0.24	62
Bm1_48395	219.0	1.45	0.0	8	0	2	1	2	5	4	0	1	3	0	1	0	0	0	0	1	na	-0.73	62
Bm1_53205	203.0	0.90	0.0	2	0	0	0	0	0	0	1	0	1	0	0	0	0	0	0	0	na	-0.55	62
Bm1_41590	119.0	0.98	53.9	2	2	0	0	0	1	1	0	0	0	0	1	0	0	0	1	0	na	0.25	61
Bm1_21255	172.0	1.89	0.0	5	3	0	0	0	0	5	0	0	0	0	0	0	0	0	0	0	na	-0.39	61
Bm1_05895	336.0	0.81	0.0	10	4	1	0	0	0	0	2	0	5	0	0	0	0	0	0	0	na	-1.05	60
Bm1_42370	124.0	0.91	0.0	5	4	1	3	1	1	0	0	0	1	0	1	0	0	0	1	0	na	-0.16	60
Bm1_55805	304.0	1.06	57.4	3	3	0	0	0	0	0	1	1	1	0	1	0	2	0	0	0	na	-0.22	59
**Bm1_03495**	174.0	0.94	0.0	4	0	0	2	1	0	0	2	0	2	0	0	0	0	0	0	0	na	-0.29	58
Bm1_10140	114.0	0.36	0.0	2	1	0	0	1	1	0	0	0	1	0	0	0	0	0	0	0	na	-0.56	58
Bm1_21225	66.2	1.01	0.0	5	6	3	0	0	0	0	2	1	0	0	2	0	2	0	1	0	na	-0.26	58
Bm1_33565	66.6	0.65	50.8	1	1	0	0	0	0	0	0	0	1	0	0	0	0	0	0	0	na	-0.26	57
Bm1_55375	50.4	0.03	48.1	2	1	2	0	1	0	0	1	0	1	0	0	0	0	0	0	0	na	0.06	57
Bm1_56035	260.0	1.03	0.0	9	0	0	0	0	0	5	0	0	4	0	0	0	0	0	0	0	na	-0.60	57
Bm1_37760	412.0	0.95	48.9	6	0	2	0	0	1	3	2	0	2	0	0	0	0	0	0	0	na	-0.67	57
Bm1_43170	304.0	0.96	0.0	5	0	0	0	0	0	0	0	4	1	0	0	0	0	0	0	0	na	0.59	55
Bm1_37230	201.0	0.73	0.0	5	0	1	3	2	1	0	2	1	1	0	0	0	0	0	0	0	na	0.09	55
Bm1_09225	157.0	1.09	58.9	1	0	1	0	0	0	0	0	0	1	0	0	0	0	0	0	0	na	-0.93	54
Bm1_36315	169.0	1.08	0.0	6	6	1	0	0	0	0	1	1	1	0	0	0	0	0	0	0	na	-0.61	54
Bm1_08450	47.0	0.34	0.0	3	0	1	1	0	0	0	3	0	0	0	0	0	0	0	0	0	na	-0.58	53
Bm1_41500	181.0	1.67	0.0	8	8	1	0	0	2	2	0	0	3	0	0	0	0	0	0	0	na	-0.85	53
Bm1_42535	220.0	0.79	0.0	6	7	0	0	0	0	0	0	0	1	0	0	0	0	0	0	0	na	-0.39	52
Bm1_51010	182.0	1.04	0.0	5	2	2	0	0	0	0	1	0	1	0	0	0	0	0	0	0	na	-0.09	52
Bm1_36555	147.0	1.02	0.0	5	0	0	4	1	0	0	0	0	3	0	3	0	5	1	5	0	na	-0.67	52
Bm1_33065	256.0	1.12	0.0	7	0	0	0	0	0	0	0	1	6	0	0	0	0	0	0	0	na	-0.60	51
Bm1_29610	109.0	1.05	0.0	2	1	0	0	0	0	0	0	0	1	0	3	0	1	0	2	1	na	-0.43	51
Bm1_54230	83.6	1.15	69.3	1	1	0	0	0	0	0	0	0	1	0	0	0	0	0	0	0	na	-0.73	51
Bm1_39200	62.4	0.83	0.0	3	0	0	0	1	1	2	1	0	1	0	0	0	0	0	0	0	na	-0.35	51
Bm1_27205	234.0	0.55	0.0	3	1	0	0	0	1	0	1	1	2	0	0	0	0	0	0	0	na	0.03	51
Bm1_05160	221.0	0.89	0.0	10	0	2	0	3	0	2	0	0	6	0	1	0	0	0	1	0	na	-1.45	51
Bm1_55745	221.0	1.01	66.6	3	1	0	0	0	0	0	0	1	2	0	0	0	0	0	0	0	superfamily	0.01	50

Bm1_39610	73.6	0.83	0.0	3	3	1	0	0	0	0	0	0	1	0	0	0	0	0	0	0	na	-0.66	50
Bm1_11590	112.0	1.14	70.5	3	3	2	0	0	0	3	1	0	1	0	0	0	0	0	0	0	na	-0.68	49
Bm1_22470	267.0	0.89	0.0	6	1	0	0	0	0	0	0	0	5	0	0	0	0	0	0	0	na	-0.23	49
Bm1_52795	0.0	0.19	0.0	3	4	0	0	0	0	1	0	0	0	0	0	0	0	0	0	0	na	-0.78	49
Bm1_23555	82.0	1.06	0.0	3	0	1	1	0	0	1	0	0	2	0	3	0	1	0	2	1	na	-0.35	49
Bm1_47145	110.0	1.11	0.0	3	0	0	3	0	0	0	0	0	1	0	0	0	0	0	0	0	na	-0.71	49
Bm1_45220	166.0	0.97	0.0	7	1	1	0	0	0	0	3	0	4	0	1	0	0	0	1	0	na	-0.81	49
Bm1_32990	244.0	0.72	0.0	7	2	1	0	1	0	0	0	0	5	0	2	0	1	0	1	0	na	-1.64	48
Bm1_08735	167.0	1.05	0.0	5	0	2	0	1	0	0	0	0	3	0	1	0	0	0	1	0	na	-1.18	48
Bm1_52640	79.0	1.05	0.0	4	3	1	0	0	0	1	1	0	0	0	0	0	0	0	0	0	na	-0.55	48
Bm1_16540	127.0	1.15	0.0	2	1	0	0	0	0	0	0	0	2	0	0	0	0	0	0	0	na	-1.01	48
Bm1_20120	207.0	0.86	0.0	7	0	0	0	0	1	2	1	0	5	0	1	0	0	0	1	0	na	-0.10	48
Bm1_40975	114.0	1.03	0.0	6	7	0	0	1	0	0	0	0	1	0	2	0	0	0	1	1	na	-0.11	48
Bm1_41700	52.4	0.57	51.6	4	6	0	0	0	0	0	0	0	0	0	0	0	0	0	0	0	Zinc finger in RANBP and others	-0.20	47
Bm1_12550	0.0	0.28	0.0	3	3	1	0	0	0	1	0	0	1	0	0	0	0	0	0	0	na	-0.45	47
Bm1_16780	161.0	0.93	0.0	5	0	2	1	2	0	0	2	0	3	0	0	0	0	0	0	0	na	0.60	47
Bm1_14325	175.0	0.93	0.0	5	0	2	1	3	0	0	0	1	2	0	0	0	0	0	0	0	na	0.21	46
Bm1_00815	193.0	0.93	0.0	6	0	0	0	0	2	0	0	0	4	0	2	0	1	0	3	0	na	-0.81	46
**Bm1_01990**	157.0	0.11	0.0	3	1	1	1	1	0	1	0	0	1	0	0	0	0	0	0	0	na	-0.86	46
Bm1_25060	164.0	1.00	0.0	5	0	1	1	1	0	0	1	0	4	0	0	0	0	0	0	0	na	-0.64	45
Bm1_51260	146.0	1.39	0.0	5	0	1	2	2	0	1	0	0	3	0	0	0	0	0	0	0	na	-0.03	45
Bm1_46165	0.0	0.98	0.0	2	0	1	0	0	0	1	0	0	1	0	4	1	1	3	2	0	na	-1.79	45
**Bm1_17930**	765.0	0.31	56.2	3	1	1	1	1	0	1	0	0	1	0	0	0	0	0	0	0	na	-0.17	44
Bm1_32875	452.0	0.84	47.0	5	0	2	0	0	0	0	0	0	3	0	0	0	0	0	0	0	na	-0.85	44
Bm1_25645	104.0	0.06	0.0	3	0	2	1	0	0	2	0	0	1	0	0	0	0	0	0	0	na	-0.41	43
Bm1_08745	0.0	0.62	0.0	3	1	0	0	0	0	3	0	0	1	0	2	0	1	0	2	0	na	-0.15	42
Bm1_09360	291.0	0.50	0.0	10	4	1	0	0	0	0	2	0	5	0	3	0	2	0	1	1	na	-1.34	42
Bm1_36270	208.0	0.32	56.2	2	0	1	0	0	0	0	0	1	1	0	2	0	1	0	3	0	TspO- and MBR-related proteins	0.18	42
Bm1_43045	0.0	0.44	0.0	3	0	0	4	1	0	0	0	0	1	0	0	0	0	0	0	0	na	-0.78	42
Bm1_34840	149.0	1.04	0.0	22	3	0	13	5	0	0	10	1	12	0	3	0	6	2	3	0	na	-0.65	42
Bm1_23570	63.5	1.38	0.0	4	0	2	0	3	1	0	1	0	2	0	0	0	0	0	0	0	na	-0.75	42
Bm1_36575	112.0	0.54	0.0	4	1	1	0	0	0	3	1	0	0	0	0	0	0	0	0	0	na	-0.05	42
Bm1_26170	218.0	0.33	0.0	3	1	0	0	0	1	0	1	1	2	0	0	0	0	0	0	0	na	-0.24	41
Bm1_28210	146.0	0.85	0.0	5	0	2	1	2	0	0	2	0	3	0	0	0	0	0	0	0	na	0.59	41
Bm1_37610	191.0	0.95	0.0	4	0	0	0	0	0	1	0	0	3	0	0	0	0	0	0	0	na	-0.46	41
Bm1_23080	116.0	1.08	43.9	4	3	1	1	0	1	1	2	1	0	0	3	0	2	0	2	1	na	-0.13	41
Bm1_42700	56.6	0.18	0.0	4	3	0	2	2	0	0	1	0	2	0	0	0	0	0	0	0	na	-0.05	41
Bm1_28670	137.0	0.99	0.0	4	0	2	1	0	0	0	0	0	2	0	0	0	0	0	0	0	na	-0.79	41
Bm1_24580	0.0	0.73	0.0	4	4	1	0	0	0	0	1	1	1	0	4	1	1	1	1	0	na	-0.64	41
Bm1_12855	59.7	1.39	0.0	4	5	0	0	0	0	0	0	0	0	0	0	0	0	0	0	0	na	-0.94	40
Bm1_22670	256.0	0.77	0.0	4	0	0	0	0	0	0	0	1	3	0	0	0	0	0	0	0	na	-0.36	40
Bm1_50710	182.0	0.68	0.0	3	0	0	0	0	0	1	0	0	2	0	1	0	0	0	1	0	na	-0.16	39
Bm1_18000	160.0	0.91	0.0	4	0	0	0	0	0	2	0	1	1	0	0	0	0	0	0	0	na	-0.34	39

Bm1_51520	184.0	0.89	0.0	5	0	0	2	0	0	0	0	0	3	0	0	0	0	0	0	0	na	-0.66	39
Bm1_24165	82.4	1.20	0.0	2	1	0	0	0	0	0	0	0	2	0	0	0	0	0	0	0	na	-0.86	39
Bm1_27515	57.8	1.05	0.0	5	5	0	1	1	2	0	0	1	1	0	0	0	0	0	0	0	na	-0.19	38
Bm1_08545	107.0	1.39	72.4	2	1	0	2	0	1	0	0	0	1	0	0	0	0	0	0	0	na	-0.72	38
Bm1_02565	0.0	0.40	0.0	5	3	1	1	1	0	2	1	0	1	0	5	1	3	3	4	1	na	-0.12	38
Bm1_04880	115.0	1.01	52.4	3	3	1	0	0	0	0	1	1	0	0	3	0	2	0	1	1	na	-0.46	38
Bm1_52140	202.0	0.94	0.0	12	1	0	0	0	0	0	0	0	10	0	0	0	0	0	0	0	na	-0.32	38
Bm1_00490	61.6	0.22	0.0	5	3	0	1	2	3	2	0	1	1	0	1	0	0	0	0	1	na	-0.35	38
Bm1_18060	194.0	0.74	0.0	3	1	0	0	0	0	0	0	0	2	0	0	0	0	0	0	0	na	-0.24	38
Bm1_50825	178.0	0.94	0.0	5	0	0	0	2	0	0	0	0	3	0	1	0	1	0	0	0	na	0.37	38
Bm1_37860	105.0	0.36	60.5	4	0	1	0	3	0	0	1	0	2	0	2	0	1	0	3	0	Aldo/keto reductase family	-0.28	38
Bm1_23385	212.0	0.50	0.0	4	1	1	0	0	0	0	1	0	2	0	0	0	0	0	0	0	na	-0.78	37
Bm1_37570	420.0	0.81	70.9	3	1	1	0	0	0	1	0	0	2	0	0	0	0	0	0	0	na	-0.33	37
**Bm1_04665**	278.0	0.34	0.0	6	2	2	1	1	1	0	1	0	3	0	1	0	2	0	0	0	na	-0.10	37
Bm1_18340	112.0	0.42	0.0	3	0	2	1	0	0	0	0	0	1	0	1	0	0	2	0	0	na	-0.09	37
Bm1_54115	96.7	0.99	0.0	6	0	3	0	0	0	1	1	1	2	0	1	0	0	0	1	0	na	-0.75	37
Bm1_54490	192.0	0.98	0.0	4	0	0	0	0	0	0	0	1	3	0	0	0	0	0	0	0	na	-0.13	37
Bm1_00215	0.0	2.19	0.0	3	3	0	0	0	0	0	0	0	1	0	2	0	1	0	1	0	na	-0.98	37
Bm1_27330	180.0	1.09	47.0	5	1	2	1	0	0	1	4	0	2	0	2	1	0	0	1	0	na	-0.82	37
Bm1_27330	180.0	1.09	47.0	5	1	2	1	0	0	1	4	0	2	0	2	1	0	0	1	0	na	-0.82	37
Bm1_15680	131.0	1.02	0.0	4	1	0	0	0	0	1	0	0	3	0	0	0	0	0	0	0	na	-0.72	37
Bm1_27280	0.0	0.80	0.0	7	9	2	4	1	3	1	1	0	1	0	1	0	1	0	0	0	na	-0.37	37
Bm1_55000	273.0	0.53	0.0	10	0	2	0	3	0	2	0	0	6	0	1	0	1	0	0	0	na	-1.11	37
Bm1_43955	203.0	0.73	67.4	2	2	1	0	0	0	0	0	0	1	0	0	0	0	0	0	0	na	-0.05	37
Bm1_08225	62.4	0.11	0.0	4	3	0	0	1	1	1	1	0	2	0	0	0	0	0	0	0	na	-0.44	36
Bm1_13550	85.9	1.05	0.0	6	1	0	2	3	2	2	0	0	3	0	0	0	0	0	0	0	na	-0.38	36
Bm1_43515	114.0	1.21	0.0	6	0	2	0	0	0	1	0	0	4	0	3	0	1	1	1	0	na	-0.47	36
Bm1_25440	0.0	0.10	0.0	5	4	2	0	0	0	2	3	0	0	0	0	0	0	0	0	0	na	0.14	36
Bm1_19985	90.5	1.00	0.0	6	0	0	4	1	0	0	0	0	4	0	3	0	2	1	2	0	na	-0.67	36
Bm1_36335	0.0	1.75	0.0	4	0	0	1	2	0	0	2	0	2	0	5	1	3	2	2	1	na	-0.64	35
Bm1_40905	575.0	0.19	0.0	5	2	1	0	0	0	0	1	0	3	0	0	0	0	0	0	0	na	-0.29	35
Bm1_50900	146.0	1.56	0.0	6	0	0	0	0	1	1	1	0	5	0	0	0	0	0	0	0	na	-0.27	35
Bm1_22525	154.0	0.94	0.0	4	1	0	0	0	0	0	0	0	3	0	0	0	0	0	0	0	na	-0.66	34
Bm1_19065	167.0	0.65	0.0	4	1	0	0	0	0	0	0	0	3	0	2	0	1	0	1	0	na	-1.20	34
Bm1_29140	92.0	0.93	0.0	3	1	0	0	0	0	0	0	0	2	0	1	0	0	0	1	0	na	-0.57	34
Bm1_17810	112.0	1.00	63.5	3	3	1	0	0	0	1	2	0	0	0	0	0	0	0	0	0	na	0.88	34
Bm1_56515	97.1	1.02	0.0	5	2	0	0	0	0	0	0	1	3	0	1	0	0	0	1	0	na	-0.81	34
Bm1_09670	122.0	0.68	0.0	7	5	1	0	0	0	0	3	1	1	0	0	0	0	0	0	0	na	-1.04	34
Bm1_46400	89.7	1.39	0.0	5	0	0	2	0	1	0	0	0	3	0	1	0	0	0	1	0	na	-0.97	33
Bm1_55790	74.7	1.11	0.0	5	0	1	0	0	0	0	4	1	1	0	0	0	0	0	0	0	na	-0.18	33
Bm1_13030	0.0	0.94	0.0	4	3	1	0	1	0	1	1	0	1	0	0	0	0	0	0	0	na	0.28	33
Bm1_10505	116.0	1.02	0.0	9	0	0	4	2	0	0	3	1	4	0	0	0	0	0	0	0	na	-0.83	32
Bm1_48195	129.0	0.90	0.0	5	0	1	0	2	0	0	1	0	3	0	0	0	0	0	0	0	na	0.10	32

Bm1_31780	127.0	0.66	0.0	3	2	0	0	0	0	0	0	0	1	0	0	0	0	0	0	0	na	0.49	32
Bm1_49770	183.0	1.03	60.8	2	2	0	0	0	0	0	0	0	1	0	0	0	0	0	0	0	na	-0.24	32
Bm1_00720	144.0	1.38	0.0	7	0	2	0	0	0	0	0	0	5	0	0	0	0	0	0	0	na	-0.58	32
Bm1_12400	57.0	1.25	0.0	4	0	2	1	0	0	0	1	0	2	0	0	0	0	0	0	0	na	-0.42	31
Bm1_23180	106.0	1.13	0.0	8	4	1	0	0	0	0	2	1	2	0	0	0	0	0	0	0	na	-0.83	31
Bm1_09610	134.0	0.72	0.0	10	0	2	0	3	0	2	0	0	6	0	4	0	4	2	3	1	na	-1.53	31
Bm1_16685	72.4	1.00	0.0	7	8	0	0	0	0	1	0	0	0	0	0	0	0	0	0	0	na	-0.73	31
Bm1_52255	105.0	0.85	0.0	3	0	0	0	1	0	0	0	0	2	0	2	0	1	0	1	0	na	-0.24	31
Bm1_07780	52.4	0.73	0.0	2	0	1	0	0	0	0	0	0	1	0	2	0	2	0	2	0	na	-0.10	31
Bm1_45405	278.0	1.00	50.8	4	0	2	0	0	0	0	1	1	1	0	0	0	0	0	0	0	na	-0.99	31
Bm1_41030	60.1	0.64	0.0	6	5	1	3	1	0	0	0	0	2	1	0	0	0	0	0	0	na	-0.84	30
Bm1_52065	0.0	1.07	0.0	7	9	0	0	1	0	1	2	0	0	0	2	0	2	0	3	0	na	-0.85	30
Bm1_23010	141.0	1.06	47.8	3	2	1	0	0	0	0	1	1	1	0	0	0	0	0	0	0	na	-0.49	30
Bm1_57645	72.0	0.54	0.0	3	1	0	0	0	0	0	1	1	1	0	0	0	0	0	0	0	na	-0.38	30
Bm1_07925	88.6	0.75	0.0	4	0	1	0	0	0	0	2	0	2	0	0	0	0	0	0	0	na	-0.32	30
Bm1_09975	166.0	0.70	0.0	4	0	0	0	0	2	0	0	0	2	0	0	0	0	0	0	0	na	-0.38	29
Bm1_52560	93.6	0.36	0.0	2	1	0	0	0	0	0	0	0	2	0	0	0	0	0	0	0	na	-0.83	29
Bm1_55970	265.0	0.41	0.0	15	14	1	0	0	1	3	0	0	3	0	0	0	0	0	0	0	na	-0.45	29
Bm1_27240	0.0	0.09	0.0	5	5	0	0	1	2	2	0	0	0	0	0	0	0	0	0	0	na	-0.06	29
Bm1_14115	92.0	0.98	0.0	4	0	0	0	2	1	0	0	0	2	0	0	0	0	0	0	0	na	-0.58	29
Bm1_04775	87.8	0.34	0.0	4	0	1	0	0	0	1	2	1	0	0	0	0	0	0	0	0	na	-0.49	29
Bm1_47280	79.7	0.53	0.0	3	1	0	1	0	0	0	0	1	2	0	0	0	0	0	0	0	na	-0.88	29
Bm1_05925	185.0	0.15	0.0	6	0	2	0	2	0	2	4	0	1	0	0	0	0	0	0	0	na	-0.58	28
Bm1_49935	127.0	1.29	70.9	4	1	0	0	0	0	0	0	0	3	0	2	0	1	0	0	1	Zinc finger in RANBP and others	-1.08	28
Bm1_21655	60.5	1.05	0.0	6	1	1	0	0	0	0	4	1	2	0	0	0	0	0	0	0	na	-0.74	28
Bm1_19420	46.2	0.39	0.0	6	5	1	0	0	0	0	1	0	2	0	1	0	0	0	1	0	na	-1.90	28
Bm1_07450	0.0	4.69	0.0	3	1	0	0	1	0	0	1	0	2	0	0	0	0	0	0	0	na	-0.64	28
Bm1_54105	57.4	0.86	0.0	5	3	0	0	0	0	2	0	0	2	0	0	0	0	0	0	0	na	-0.53	27
Bm1_12525	313.0	0.16	0.0	5	4	1	0	0	0	0	0	0	3	0	0	0	0	0	0	0	na	-0.33	27
Bm1_13915	150.0	0.73	0.0	4	1	0	0	0	0	0	0	0	3	0	0	0	0	0	0	0	na	-0.28	27
Bm1_39000	55.1	0.85	0.0	5	0	2	0	0	0	1	1	0	4	0	2	0	1	3	0	0	na	-0.28	27
Bm1_54705	79.0	1.04	0.0	8	0	1	2	0	0	0	0	0	5	0	3	0	7	2	2	0	na	-0.93	26
Bm1_05210	0.0	0.08	0.0	4	0	1	3	1	1	0	0	0	1	0	0	0	0	0	0	0	na	-1.80	26
Bm1_56880	0.0	0.74	0.0	4	2	3	1	0	0	0	0	0	0	0	0	0	0	0	0	0	na	0.24	26
Bm1_17365	234.0	0.19	0.0	5	4	1	0	0	0	0	0	0	3	0	0	0	0	0	0	0	na	-0.50	26
Bm1_40345	84.7	1.09	0.0	6	1	0	0	0	0	0	0	0	5	0	1	0	0	0	1	0	na	-0.33	26
Bm1_52595	132.0	1.31	0.0	6	0	1	0	0	0	0	0	0	5	0	0	0	0	0	0	0	na	0.06	26
Bm1_38270	311.0	0.83	48.9	3	0	0	0	0	0	1	0	1	2	0	1	0	1	0	0	0	na	-1.12	26
Bm1_06665	304.0	1.05	50.8	6	1	0	0	0	0	0	0	0	5	0	1	0	0	0	1	0	na	0.40	26
Bm1_39335	145.0	1.30	0.0	7	0	0	0	0	0	0	0	2	5	0	0	0	0	0	0	0	na	0.42	25
Bm1_41530	96.3	0.68	0.0	6	0	0	4	2	0	0	0	0	4	0	0	0	0	0	0	0	na	-0.54	25
Bm1_00640	0.0	0.28	0.0	6	3	2	2	0	0	3	1	0	1	0	0	0	0	0	0	0	na	-0.74	25
Bm1_06810	154.0	1.05	48.5	4	0	1	3	1	1	0	0	0	1	0	1	0	0	0	1	0	na	-0.65	25

Bm1_18685	222.0	0.96	49.3	7	4	0	1	0	0	0	3	0	2	0	3	0	1	0	3	1	na	-0.37	25
Bm1_49180	97.8	1.03	0.0	2	0	0	0	0	0	0	0	1	0	0	1	0	2	0	0	0	na	-0.90	25
Bm1_19740	126.0	0.20	0.0	3	2	0	0	0	0	0	0	0	1	0	0	0	0	0	0	0	na	-0.52	25
Bm1_45285	68.6	0.92	0.0	5	0	2	0	0	0	0	0	0	3	0	2	0	1	0	1	0	na	-0.37	25
Bm1_53210	0.0	0.52	0.0	2	0	0	0	0	0	0	1	0	1	0	0	0	0	0	0	0	na	-0.92	25
Bm1_37315	64.3	1.00	0.0	4	0	0	0	0	0	1	0	0	3	0	1	0	0	0	1	0	na	-0.82	25
Bm1_07795	95.9	0.91	0.0	6	2	0	0	0	0	1	0	0	4	0	0	0	0	0	0	0	na	-0.44	25
Bm1_11840	54.3	1.79	0.0	4	0	0	0	0	0	0	1	0	3	0	2	0	0	0	2	3	na	-0.66	24
Bm1_21815	97.8	0.86	0.0	6	0	1	0	0	0	0	2	1	3	0	0	0	0	0	0	0	na	-0.29	24
Bm1_32260	94.7	1.99	0.0	7	0	1	1	1	0	0	0	4	2	0	0	0	0	0	0	0	na	-0.99	24
Bm1_04070	47.8	0.14	0.0	2	1	0	0	0	0	0	0	0	2	0	0	0	0	0	0	0	na	-1.01	24
Bm1_50790	0.0	0.69	0.0	7	7	0	0	0	0	1	1	1	0	0	2	0	0	0	1	1	na	-0.42	24
Bm1_22765	79.0	0.30	0.0	3	1	0	0	0	0	1	0	0	2	0	0	0	0	0	0	0	na	-0.65	24
Bm1_22905	112.0	0.97	0.0	4	1	0	0	0	0	0	0	0	3	0	0	0	0	0	0	0	na	0.36	24
Bm1_38370	139.0	0.85	0.0	7	0	0	0	0	0	0	0	0	6	1	0	0	0	0	0	0	na	-0.90	24
Bm1_15075	97.4	0.87	0.0	6	0	0	0	0	1	1	1	0	5	0	0	0	0	0	0	0	na	-0.42	23
Bm1_14650	87.8	0.86	0.0	7	0	0	1	0	0	4	0	0	4	0	0	0	0	0	0	0	na	-0.45	23
Bm1_55755	85.9	1.40	0.0	21	0	0	0	0	0	0	0	1	20	0	1	0	0	0	2	0	na	-0.20	23
Bm1_55755	85.9	1.40	0.0	22	0	0	0	0	0	0	0	1	21	0	1	0	0	0	2	0	na	-0.20	23
Bm1_53230	56.2	0.93	0.0	5	0	2	0	0	0	0	0	0	3	0	3	0	1	0	1	1	na	-0.92	23
Bm1_53630	0.0	0.54	0.0	5	0	1	1	2	0	0	3	0	2	0	0	0	0	0	0	0	na	-0.66	23
Bm1_09930	0.0	0.06	0.0	2	1	0	0	0	0	0	0	0	1	0	0	0	0	0	0	0	na	-0.51	23
Bm1_23380	100.0	0.31	0.0	4	1	1	0	0	0	0	1	0	2	0	0	0	0	0	0	0	na	-0.50	22
Bm1_15990	225.0	1.00	68.9	4	2	1	2	0	0	0	1	1	1	0	0	0	0	0	0	0	na	-1.01	22
Bm1_09120	70.1	0.88	0.0	3	1	0	0	0	0	0	0	0	2	0	0	0	0	0	0	0	na	-0.75	22
Bm1_46225	108.0	1.06	0.0	7	0	1	0	0	0	0	0	0	6	0	0	0	0	0	0	0	na	-0.24	22
Bm1_21885	103.0	3.31	0.0	6	1	0	0	0	0	0	0	0	5	0	0	0	0	0	0	0	na	-0.20	22
Bm1_46355	94.0	0.79	0.0	10	7	0	0	0	1	1	0	0	4	0	0	0	0	0	0	0	na	-0.28	22
Bm1_28165	97.4	0.31	0.0	5	0	0	0	2	2	0	0	0	3	0	1	0	0	0	1	0	na	-0.49	22
Bm1_01765	70.1	1.00	0.0	8	0	0	0	0	0	0	0	3	5	0	5	1	2	1	2	1	na	0.16	22
Bm1_01505	0.0	0.28	0.0	3	2	0	0	0	0	0	0	0	2	0	3	0	1	1	2	0	na	0.67	22
Bm1_44095	81.6	0.84	0.0	3	1	0	0	0	0	0	0	0	2	0	0	0	0	0	0	0	na	0.05	22
Bm1_19440	108.0	0.96	54.7	4	2	1	2	0	0	1	1	1	1	0	1	0	0	0	1	0	na	-0.60	21
Bm1_34110	51.2	1.14	0.0	9	5	2	0	0	0	0	3	0	3	0	0	0	0	0	0	0	na	-0.51	21
Bm1_36295	119.0	0.55	0.0	7	1	0	0	0	0	0	1	1	5	0	1	0	0	0	0	1	na	-1.08	21
Bm1_06340	126.0	0.83	0.0	7	0	0	0	0	0	0	0	1	5	0	0	0	0	0	0	0	na	-0.54	21
Bm1_50365	56.2	1.14	0.0	5	1	0	0	0	0	1	0	0	4	0	1	0	1	0	0	0	na	-1.51	21
Bm1_41110	137.0	0.86	0.0	8	0	0	0	0	0	1	0	0	7	0	0	0	0	0	0	0	na	0.40	21
Bm1_53505	427.0	0.13	0.0	10	2	1	0	1	0	3	4	0	5	0	0	0	0	0	0	0	na	-0.90	21
Bm1_35045	73.2	1.06	0.0	3	0	0	0	0	0	0	0	1	2	0	1	0	0	0	1	0	na	0.06	20
Bm1_38610	234.0	3.91	55.8	3	0	0	0	0	0	0	1	0	3	0	2	0	1	0	1	0	na	-0.96	20
Bm1_13170	119.0	0.42	0.0	9	0	4	1	0	1	4	1	0	5	0	0	0	0	0	0	0	na	-0.35	20
Bm1_32180	126.0	0.81	0.0	7	0	2	0	0	0	0	0	0	5	0	0	0	0	0	0	0	na	0.27	20

Bm1_07440	0.0	0.06	0.0	2	0	0	0	0	0	1	0	0	1	0	0	0	0	0	0	0	na	-0.69	20
Bm1_26400	381.0	0.17	0.0	5	1	0	1	0	0	0	0	0	4	0	0	0	0	0	0	0	na	-0.69	20
Bm1_25025	78.6	0.84	0.0	9	7	0	0	0	0	0	0	0	3	0	0	0	0	0	0	0	na	-0.14	20
Bm1_43720	218.0	0.84	69.7	5	4	1	3	0	1	1	2	1	2	0	0	0	0	0	0	0	na	-0.11	20
Bm1_10260	0.0	0.10	0.0	5	1	1	0	0	1	1	2	0	1	0	0	0	0	0	0	0	na	-1.38	20
Bm1_37335	131.0	0.05	0.0	8	0	1	6	4	0	0	0	0	4	0	1	0	0	0	4	0	na	-0.18	20
Bm1_20495	68.2	1.00	0.0	5	0	2	0	0	0	0	0	0	3	0	0	0	0	0	0	0	na	-0.77	19
Bm1_33635	123.0	0.57	0.0	5	1	0	0	0	0	1	0	0	3	0	0	0	0	0	0	0	na	-0.10	19
Bm1_54575	101.0	0.06	0.0	4	0	0	0	2	0	0	1	0	2	0	1	0	0	0	0	1	na	-0.46	19
Bm1_03645	178.0	0.39	0.0	4	1	0	0	0	0	0	0	0	3	0	0	0	0	0	0	0	na	-0.27	19
Bm1_10215	0.0	0.11	0.0	9	11	1	3	0	0	1	1	0	1	0	0	0	0	0	0	0	na	-1.05	18
Bm1_00120	62.4	1.09	47.0	4	4	2	0	0	0	1	0	0	1	0	1	0	0	0	1	0	na	-0.94	18
Bm1_21210	102.0	0.45	0.0	5	0	1	0	2	0	0	1	0	3	0	0	0	0	0	0	0	na	0.15	18
Bm1_01060	0.0	0.87	0.0	5	1	2	0	2	0	0	0	0	3	0	1	0	0	0	0	1	na	-1.23	18
Bm1_45055	64.7	1.10	0.0	4	0	0	0	0	0	1	0	0	3	0	0	0	0	0	0	0	na	-0.53	18
Bm1_10475	48.9	1.08	0.0	7	1	0	0	0	0	1	0	1	5	0	1	0	0	0	0	1	na	-0.76	17
Bm1_46520	55.5	0.74	0.0	4	2	0	0	0	0	0	0	0	3	0	0	0	0	0	0	0	na	0.03	17
Bm1_14055	91.7	0.52	0.0	5	0	1	0	0	0	2	0	0	4	0	0	0	0	0	0	0	na	-0.10	17
Bm1_22500	66.2	0.55	0.0	8	3	1	1	0	0	1	1	3	2	0	0	0	0	0	0	0	na	-0.59	17
Bm1_46015	84.7	0.99	0.0	8	2	0	0	0	0	0	0	0	5	0	0	0	0	0	0	0	na	0.11	17
Bm1_06310	47.0	0.71	0.0	5	1	0	0	1	0	0	1	0	4	0	0	0	0	0	0	0	na	-0.59	17
Bm1_18740	153.0	0.06	0.0	8	0	1	0	3	0	1	6	0	4	0	0	0	0	0	0	0	na	-0.71	16
Bm1_47930	373.0	0.08	0.0	14	4	1	6	5	2	2	5	0	6	0	0	0	0	0	0	0	na	-0.46	16
Bm1_05890	175.0	0.16	0.0	10	4	1	0	0	0	0	2	0	5	0	2	0	1	0	0	1	na	-1.05	16
Bm1_03770	0.0	0.41	0.0	4	0	0	1	2	1	0	0	1	1	0	0	0	0	0	0	0	na	-0.34	16
Bm1_25120	0.0	0.94	0.0	4	3	0	0	0	0	0	0	0	2	0	0	0	0	0	0	0	na	-0.15	16
Bm1_38875	0.0	1.00	0.0	6	1	1	0	1	2	3	0	0	2	0	0	0	0	0	0	0	na	-0.40	16
Bm1_13600	232.0	1.00	47.4	81	1	0	0	0	0	0	0	3	77	0	1	0	0	0	4	0	na	-0.67	15
Bm1_23015	102.0	0.83	57.4	4	3	2	1	2	0	0	1	0	1	0	0	0	0	0	0	0	na	-0.58	15
Bm1_13605	234.0	1.97	48.1	75	1	1	0	0	0	1	0	3	70	0	1	0	0	0	8	0	na	-0.69	15
Bm1_33105	0.0	0.79	0.0	9	0	2	1	0	0	2	4	5	1	0	1	0	0	0	1	0	na	0.00	15
Bm1_22560	0.0	0.01	0.0	5	0	0	2	2	2	0	0	0	2	0	0	0	0	0	0	0	na	-0.64	15
Bm1_10860	70.9	1.22	0.0	9	0	1	0	0	0	0	0	0	8	0	0	0	0	0	0	0	na	-0.42	15
Bm1_22820	69.3	1.01	0.0	7	1	0	0	0	0	0	0	0	5	1	1	1	0	0	0	0	na	0.02	15
Bm1_46970	124.0	0.82	0.0	7	0	0	0	0	0	0	0	1	6	0	0	0	0	0	0	0	na	0.80	15
Bm1_38880	105.0	1.02	53.5	6	6	4	2	0	0	1	0	0	2	0	2	0	0	1	3	0	na	-0.31	14
Bm1_46130	92.4	0.53	0.0	5	0	1	0	1	0	0	1	0	3	0	0	0	0	0	0	0	na	0.60	14
Bm1_42730	107.0	0.81	45.8	4	2	2	0	0	0	0	1	0	1	0	1	0	0	0	1	0	na	-0.43	14
Bm1_09160	76.3	0.66	0.0	6	0	2	0	0	0	0	0	0	4	0	0	0	0	0	0	0	na	-0.85	14
Bm1_29320	49.7	0.27	0.0	10	0	1	7	4	3	1	0	0	5	0	0	0	0	0	0	0	na	-0.62	14
Bm1_57650	181.0	1.00	64.3	3	0	0	0	1	4	0	0	0	1	0	0	0	0	0	0	0	na	-0.65	14
Bm1_56480	107.0	0.91	68.6	3	4	0	0	0	0	1	0	0	0	0	0	0	0	0	0	0	na	-0.67	14
Bm1_26235	124.0	0.42	0.0	6	1	0	0	0	0	0	0	0	5	0	0	0	0	0	0	0	na	-0.66	13

Bm1_55690	106.0	0.98	43.5	4	2	1	0	0	0	0	1	0	2	0	1	0	0	1	0	0	na	-0.57	13
Bm1_10660	67.0	0.56	0.0	4	1	0	0	0	0	0	0	0	4	0	0	0	0	0	0	0	na	-0.34	13
Bm1_41635	0.0	3.54	0.0	6	0	1	1	1	0	0	3	0	3	0	0	0	0	0	0	0	na	-0.10	13
Bm1_51715	119.0	0.92	54.3	4	3	1	0	0	0	1	1	0	1	0	1	0	1	0	0	0	na	-0.14	13
Bm1_40800	126.0	0.93	60.5	4	2	0	0	2	0	0	3	0	2	0	0	0	0	0	0	0	na	-0.51	13
Bm1_50395	0.0	1.56	0.0	4	1	0	0	0	0	0	1	1	2	0	0	0	0	0	0	0	na	-0.49	13
Bm1_53760	110.0	1.15	58.2	4	2	1	1	0	0	1	1	0	1	0	2	0	2	1	0	0	na	-1.24	12
Bm1_28945	279.0	0.97	53.5	5	0	0	0	0	0	1	0	0	4	0	0	0	0	0	0	0	na	0.12	12
Bm1_50000	64.7	0.74	0.0	6	1	0	0	0	0	0	0	0	5	0	0	0	0	0	0	0	na	-0.44	12
Bm1_53515	132.0	0.09	0.0	10	2	1	0	1	0	3	4	0	5	0	0	0	0	0	0	0	na	-1.26	12
Bm1_33500	52.8	0.79	0.0	6	0	0	0	1	2	0	0	0	4	0	0	0	0	0	0	0	na	-0.21	12
Bm1_36265	67.4	1.83	57.0	6	3	3	0	3	0	2	5	1	1	0	1	0	0	0	1	0	na	-0.75	12
Bm1_29880	252.0	1.10	73.9	3	1	0	0	0	0	0	1	0	1	0	0	0	0	0	0	0	na	-0.28	12
Bm1_52720	97.8	0.43	46.2	5	4	3	1	0	0	2	1	0	2	0	1	0	0	0	0	1	na	-0.82	12
Bm1_21695	97.1	1.06	48.5	3	0	0	0	0	0	1	2	0	2	0	0	0	0	0	0	0	na	-0.61	12
Bm1_23935	65.5	1.00	0.0	7	0	0	0	0	0	0	0	3	4	0	0	0	0	0	0	0	na	0.01	12
Bm1_06925	121.0	0.21	0.0	5	1	0	1	0	0	0	0	0	4	0	0	0	0	0	0	0	na	-0.33	12
Bm1_07615	79.0	1.80	56.6	2	0	1	0	0	0	0	1	0	1	0	0	0	0	0	0	0	na	-0.11	12
Bm1_46775	72.0	0.25	0.0	3	1	0	0	0	0	0	0	0	3	0	0	0	0	0	0	0	na	0.00	12
Bm1_29000	0.0	1.10	0.0	4	2	0	0	0	0	0	0	0	2	0	0	0	0	0	0	0	na	-0.89	11
Bm1_27615	55.1	0.65	46.6	4	0	0	2	0	3	3	0	0	1	0	1	0	0	0	1	0	na	-0.71	11
Bm1_32720	50.4	1.40	0.0	3	0	0	0	0	0	0	0	1	2	0	0	0	0	0	0	0	na	-0.68	11
Bm1_07485	0.0	0.25	0.0	12	7	3	4	2	1	2	1	1	5	0	0	0	0	0	0	0	na	-1.00	11
Bm1_26745	75.5	0.85	0.0	7	0	0	0	0	0	0	0	1	6	0	0	0	0	0	0	0	na	0.01	11
Bm1_18115	62.8	0.65	0.0	5	1	0	0	0	0	0	0	0	4	0	0	0	0	0	0	0	na	-0.22	11
Bm1_00910	228.0	0.87	48.5	4	0	0	0	0	0	2	0	1	1	0	0	0	0	0	0	0	na	-0.30	11
Bm1_43075	71.2	0.57	0.0	6	1	0	0	0	0	0	0	0	5	0	0	0	0	0	0	0	na	-1.04	11
Bm1_02140	213.0	0.04	0.0	10	2	1	0	1	0	3	4	0	5	0	0	0	0	0	0	0	na	-0.28	11
Bm1_53390	60.8	0.19	0.0	3	0	1	0	0	0	0	0	1	2	0	0	0	0	0	0	0	na	-0.21	10
Bm1_52605	81.3	0.55	0.0	7	1	0	0	0	0	1	0	0	6	0	0	0	0	0	0	0	na	0.05	10
Bm1_05820	0.0	0.10	0.0	3	1	0	0	0	0	0	0	0	3	0	0	0	0	0	0	0	na	-0.77	10
Bm1_54895	82.4	0.37	0.0	4	0	0	0	0	0	1	0	0	3	0	0	0	0	0	0	0	na	-0.17	10
Bm1_37705	86.3	0.86	59.3	4	3	1	0	1	0	1	2	0	2	0	0	0	0	0	0	0	na	-0.60	10
Bm1_16040	53.1	0.30	0.0	7	1	0	0	0	0	0	0	0	6	0	3	0	2	1	1	0	na	0.05	10
Bm1_02065	112.0	0.34	0.0	14	1	1	0	2	2	0	0	0	11	0	0	0	0	0	0	0	na	-0.66	9
Bm1_41775	73.9	1.16	55.5	5	2	1	0	1	0	1	4	1	2	1	1	0	0	0	1	0	na	-0.73	9
Bm1_19380	45.8	1.19	0.0	13	0	0	0	0	0	0	0	0	12	1	0	0	0	0	0	0	na	-0.59	9
Bm1_56530	241.0	0.77	68.6	6	4	0	1	0	1	1	4	0	1	0	0	0	0	0	0	0	na	-0.44	9
Bm1_30695	120.0	0.96	53.5	2	1	0	0	0	0	0	0	0	2	0	1	0	0	0	2	0	na	-0.26	9
Bm1_32280	99.4	0.18	0.0	5	0	1	0	2	0	0	0	0	3	0	0	0	0	0	0	0	na	0.10	9
Bm1_24615	233.0	1.05	71.2	8	3	2	0	0	0	3	1	1	2	0	3	0	2	0	1	1	na	-0.52	9
Bm1_09195	97.4	0.11	47.8	3	2	0	0	2	0	0	1	0	1	0	0	0	0	0	0	0	na	-0.96	8
Bm1_42645	84.0	0.21	0.0	4	0	0	0	0	0	0	1	0	3	0	0	0	0	0	0	0	na	-0.07	8

Bm1_01030	49.3	0.46	0.0	5	1	0	0	0	0	0	0	0	4	0	0	0	0	0	0	0	na	-0.38	8
Bm1_50115	95.9	0.95	67.4	5	5	2	1	3	0	2	1	2	1	0	0	0	0	0	0	0	na	-0.07	8
Bm1_26495	0.0	0.08	0.0	3	1	0	0	0	0	0	0	0	2	0	0	0	0	0	0	0	na	0.13	7
Bm1_46050	50.1	0.55	0.0	11	0	1	0	1	0	1	0	0	9	0	0	0	0	0	0	0	na	-0.56	7
Bm1_01100	0.0	2.14	0.0	10	2	4	2	0	0	0	1	0	4	0	0	0	0	0	0	0	na	-0.44	7
Bm1_27360	100.0	0.20	0.0	4	1	0	0	0	0	0	0	0	3	0	0	0	0	0	0	0	na	0.08	7
Bm1_35060	136.0	1.07	48.1	3	1	0	0	0	0	0	0	1	2	0	2	0	2	0	1	0	na	-1.31	7
Bm1_10425	0.0	0.03	0.0	5	0	0	0	0	1	2	0	0	3	0	0	0	0	0	0	0	na	-0.06	7
Bm1_44890	307.0	0.75	58.9	3	1	0	0	0	0	0	0	0	2	0	0	0	0	0	0	0	na	0.09	6
Bm1_42145	70.5	0.44	0.0	14	0	0	0	0	0	0	0	0	11	3	0	0	0	0	0	0	na	-0.43	6
Bm1_06460	94.4	0.42	0.0	4	0	0	0	0	0	0	0	1	3	0	0	0	0	0	0	0	na	0.06	6
Bm1_56405	0.0	1.23	0.0	5	1	1	0	0	0	0	0	0	3	0	0	0	0	0	0	0	na	-0.32	6
Bm1_23670	67.8	0.18	0.0	7	0	1	0	0	0	0	1	0	5	0	0	0	0	0	0	0	na	-1.04	6
Bm1_34425	152.0	1.08	60.1	4	0	1	2	0	0	2	0	0	1	0	0	0	0	0	0	0	na	-0.24	6
Bm1_08610	130.0	0.22	0.0	14	0	0	0	0	0	0	0	0	7	7	0	0	0	0	0	0	na	-1.02	6
Bm1_05190	0.0	0.67	0.0	4	1	0	0	0	0	0	0	0	3	0	0	0	0	0	0	0	na	-0.57	6
Bm1_13195	0.0	0.39	0.0	4	1	0	0	0	0	0	0	0	3	0	0	0	0	0	0	0	na	-1.50	6
Bm1_18845	149.0	0.89	63.2	6	6	1	0	0	0	0	2	0	2	0	1	0	0	0	3	0	na	-0.57	6
Bm1_19290	0.0	0.27	0.0	10	0	0	0	0	0	0	1	0	10	0	1	0	0	0	0	1	na	-0.67	5
Bm1_01955	0.0	0.15	0.0	9	7	0	3	1	0	1	1	0	1	0	0	0	0	0	0	0	na	1.39	5
Bm1_17130	99.0	0.04	50.8	3	2	0	0	2	0	0	1	0	1	0	0	0	0	0	0	0	na	-1.09	5
Bm1_09640	141.0	1.04	53.1	4	0	0	3	1	0	0	0	0	1	0	0	0	0	0	0	0	na	-0.59	4
Bm1_57420	137.0	0.50	73.2	2	1	0	0	1	1	0	0	0	1	0	0	0	0	0	0	0	na	-0.26	4
Bm1_09565	328.0	0.83	57.0	5	0	0	0	0	0	0	0	4	1	0	0	0	0	0	0	0	na	0.77	4
Bm1_03010	71.2	0.38	0.0	8	0	0	0	0	0	0	0	1	6	0	0	0	0	0	0	0	na	-0.16	4
Bm1_10315	50.8	0.13	0.0	6	1	1	0	0	0	0	0	0	5	0	0	0	0	0	0	0	na	-0.09	4
Bm1_35660	130.0	1.17	65.9	5	5	1	1	0	0	0	1	0	1	0	2	0	1	0	1	0	na	0.44	4
Bm1_21040	62.8	0.28	0.0	10	0	0	0	0	0	0	0	4	6	0	0	0	0	0	0	0	na	-0.95	4
Bm1_06045	0.0	0.23	0.0	8	1	0	0	1	0	0	1	0	7	0	0	0	0	0	0	0	na	-0.64	4
Bm1_49240	0.0	0.13	0.0	5	0	2	0	0	0	0	0	1	3	0	0	0	0	0	0	0	na	0.12	3
Bm1_28060	200.0	1.05	63.2	4	0	0	0	0	0	0	2	2	2	0	0	0	0	0	0	0	na	-0.66	3
Bm1_13520	152.0	1.32	48.9	9	4	1	0	0	0	0	2	0	4	0	1	0	0	0	0	1	na	-0.60	3
Bm1_20745	102.0	0.24	72.0	4	0	0	0	0	0	0	1	1	3	0	0	0	0	0	0	0	EC:3.5.4.4	-0.17	3
Bm1_39165	96.7	0.17	66.6	4	0	0	3	2	0	1	4	0	1	0	0	0	0	0	0	0	na	-0.42	2
Bm1_16530	200.0	0.68	58.2	5	0	2	1	2	0	0	2	0	3	0	0	0	0	0	0	0	na	-0.79	2
Bm1_43570	157.0	0.90	63.9	6	7	1	1	1	2	0	0	1	0	0	0	0	0	0	0	0	na	-0.05	2
Bm1_24565	0.0	1.44	0.0	6	1	0	0	0	0	0	0	0	5	0	0	0	0	0	0	0	na	-0.26	2
Bm1_15165	0.0	0.19	0.0	8	2	0	0	0	0	1	0	0	5	0	0	0	0	0	0	0	na	0.09	2
Bm1_29960	268.0	0.98	65.5	10	0	0	2	0	0	0	0	1	7	0	0	0	0	0	0	0	na	-0.40	2
Bm1_52385	156.0	0.90	58.2	2	0	0	0	0	0	1	0	0	1	0	0	0	0	0	0	0	na	-0.89	1
Bm1_42980	169.0	0.99	47.4	4	0	0	0	0	0	0	0	1	3	0	2	0	2	1	0	0	na	-0.43	1
Bm1_08025	0.0	0.41	0.0	5	1	0	0	0	0	0	0	0	4	0	0	0	0	0	0	0	na	0.08	1
Bm1_03155	0.0	0.13	0.0	17	2	0	0	1	0	0	1	0	15	0	0	0	0	0	0	0	na	-0.62	1

Bm1_06290	0.0	0.63	0.0	9	1	0	0	0	0	0	0	0	8	0	0	0	0	0	0	0	na	-0.39	1
Bm1_14965	130.0	1.02	73.6	4	1	0	0	1	0	0	4	1	1	0	0	0	0	0	0	0	na	-0.13	1
Bm1_00705	0.0	0.25	0.0	9	0	0	0	1	0	0	0	0	8	0	0	0	0	0	0	0	na	-1.16	1
Bm1_06655	0.0	0.81	0.0	4	0	0	0	0	0	0	0	1	3	0	0	0	0	0	0	0	na	-1.12	1
Bm1_19425	0.0	0.17	0.0	4	0	0	0	0	0	0	0	1	3	0	0	0	0	0	0	0	na	-1.84	1
Bm1_36080	84.7	0.98	60.1	6	8	1	2	1	0	0	0	0	0	0	2	0	2	0	0	1	na	-0.75	1
Bm1_02410	0.0	0.13	0.0	6	0	0	0	0	0	0	0	2	4	0	0	0	0	0	0	0	na	-0.34	0
Bm1_13005	0.0	0.15	0.0	7	0	0	0	0	0	0	0	1	6	0	0	0	0	0	0	0	na	-1.19	0
Bm1_43725	109.0	0.31	67.4	5	4	1	3	0	1	1	2	1	2	0	1	0	0	0	1	0	na	-0.39	0
Bm1_31870	146.0	0.99	68.2	7	4	1	2	2	1	1	1	0	5	0	1	0	0	0	2	0	na	-0.35	0
Bm1_08175	87.8	0.78	50.4	5	6	0	0	0	1	1	0	0	0	0	0	0	0	0	0	0	na	-0.72	0
Bm1_00430	0.0	1.00	0.0	10	0	0	0	0	0	0	0	0	7	3	0	0	0	0	0	0	na	-2.88	0
Bm1_28625	203.0	1.03	66.6	7	0	2	1	1	0	0	0	0	4	0	1	0	0	0	1	0	na	-0.39	0
Bm1_28315	122.0	0.93	57.4	5	0	1	2	2	2	1	0	0	3	0	1	0	1	0	0	0	na	-0.15	0
Bm1_32025	44.3	0.28	0.0	6	0	0	0	0	0	0	0	2	4	0	0	0	0	0	0	0	na	0.28	0
Bm1_03765	0.0	0.11	0.0	4	0	0	0	0	0	0	0	1	3	0	0	0	0	0	0	0	na	-0.19	0
Bm1_51735	144.0	1.40	46.6	3	0	1	0	0	0	0	0	0	2	0	0	0	0	0	0	0	na	-0.93	-1
Bm1_13150	72.8	0.47	55.5	3	2	0	1	1	0	0	0	1	1	0	0	0	0	0	0	0	na	-0.50	-1
Bm1_27705	215.0	1.02	63.9	12	0	0	0	0	0	0	0	0	6	6	3	0	1	0	1	1	na	-0.60	-1
Bm1_34300	118.0	0.73	48.1	5	0	0	5	1	1	0	0	0	1	0	0	0	0	0	0	0	na	-0.29	-2
Bm1_22990	231.0	0.78	61.6	7	3	1	0	1	1	1	0	1	1	0	0	0	0	0	0	0	na	-0.45	-2
Bm1_25910	215.0	0.91	67.0	9	4	1	0	3	0	0	4	0	1	1	0	0	0	0	0	0	na	-0.61	-2
Bm1_25810	57.4	0.30	43.5	3	0	0	0	1	4	0	0	0	1	0	0	0	0	0	0	0	na	-0.71	-2
Bm1_33765	111.0	0.92	66.2	6	3	2	2	0	2	2	0	0	3	0	1	0	0	0	1	0	na	-0.61	-3
Bm1_34145	63.9	0.99	55.8	3	2	0	0	0	0	0	1	1	2	0	0	0	0	0	0	0	na	0.24	-3
Bm1_14240	204.0	0.89	59.7	5	0	0	0	0	1	2	0	0	3	0	0	0	0	0	0	0	na	-0.59	-3
Bm1_19990	108.0	1.12	50.4	6	0	0	4	1	0	0	0	0	4	0	2	0	1	1	0	0	na	-0.66	-3
Bm1_29435	170.0	0.56	53.5	3	0	1	1	0	0	1	0	0	2	0	0	0	0	0	0	0	na	0.59	-3
Bm1_33020	128.0	0.78	46.6	3	0	0	1	0	0	0	0	0	2	0	1	0	0	0	1	0	na	0.00	-4
Bm1_04725	68.2	0.36	62.4	3	2	0	0	1	1	1	0	0	2	0	0	0	0	0	0	0	na	-0.61	-4
Bm1_35390	114.0	1.30	74.3	5	5	1	2	0	1	1	0	0	0	0	0	0	0	0	0	0	na	-0.88	-4
Bm1_39400	115.0	0.73	57.8	2	0	0	0	0	0	0	1	0	1	0	0	0	0	0	0	0	na	-0.68	-4
Bm1_07190	78.2	0.86	71.6	2	0	1	0	0	0	0	1	0	1	0	0	0	0	0	0	0	na	0.21	-4
Bm1_05435	159.0	0.70	50.8	9	1	3	3	5	3	0	3	1	4	0	0	0	0	0	0	0	na	0.06	-5
Bm1_37515	0.0	0.34	0.0	5	0	0	0	0	0	0	0	1	4	0	0	0	0	0	0	0	na	0.63	-5
Bm1_34195	280.0	0.37	61.2	5	0	2	1	3	0	0	0	0	2	0	1	0	0	0	1	0	na	-0.75	-5
Bm1_33855	154.0	1.93	73.6	5	1	3	0	0	0	0	1	0	2	0	2	0	1	0	1	0	na	-0.34	-5
Bm1_30505	174.0	0.61	53.1	4	0	2	0	0	0	2	0	0	2	0	0	0	0	0	0	0	na	0.18	-5
Bm1_55395	53.5	0.42	45.1	5	5	2	0	0	1	1	0	1	1	0	0	0	0	0	0	0	na	0.26	-6
Bm1_21970	79.3	0.98	47.0	6	0	0	2	3	2	3	0	0	3	0	0	0	0	0	0	0	na	0.10	-6
Bm1_13050	56.2	0.27	48.1	4	1	2	1	0	0	2	0	0	2	0	0	0	0	0	0	0	na	-0.55	-6
Bm1_03920	124.0	1.06	65.5	6	3	3	0	0	0	1	2	0	2	0	0	0	0	0	0	0	na	-0.47	-6
Bm1_06785	97.1	1.08	57.4	3	2	0	0	0	0	0	0	0	2	0	0	0	0	0	0	0	na	-0.92	-7

Bm1_22805	63.2	0.52	62.0	5	2	1	0	0	0	1	4	0	1	0	2	0	0	1	2	0	na	-0.41	-7
Bm1_02630	63.9	1.07	59.3	5	6	1	1	0	0	0	0	0	1	0	0	0	0	0	0	0	na	-0.82	-7
Bm1_17210	159.0	0.98	52.8	4	1	0	0	0	0	0	0	0	3	0	0	0	0	0	0	0	na	-0.08	-7
Bm1_29430	132.0	1.05	67.4	7	4	3	1	0	0	1	2	2	1	0	0	0	0	0	0	0	na	-0.59	-8
Bm1_10835	52.4	0.99	60.5	5	7	0	0	0	1	1	0	0	0	0	0	0	0	0	0	0	na	-0.21	-9
Bm1_31055	134.0	1.11	65.5	4	1	1	0	0	0	0	0	0	3	0	2	0	1	0	2	0	na	-1.26	-9
Bm1_44000	132.0	0.65	55.1	3	1	0	0	0	0	0	0	1	1	0	2	0	3	0	3	0	na	-1.10	-9
Bm1_41915	89.7	0.59	69.3	4	3	0	0	0	0	0	3	0	1	0	0	0	0	0	0	0	na	-0.51	-9
Bm1_16675	87.0	1.10	61.6	5	1	0	0	0	0	0	4	0	1	0	1	0	1	0	0	0	na	-0.97	-9
Bm1_36460	101.0	1.07	56.6	7	1	1	1	1	0	0	1	3	2	0	4	0	1	1	1	1	na	-0.54	-9
Bm1_52475	82.8	0.76	64.3	5	2	2	3	2	1	0	0	0	2	0	0	0	0	0	0	0	na	-0.61	-10
Bm1_01075	0.0	1.00	0.0	3	0	0	0	0	0	0	1	0	3	0	0	0	0	0	0	0	na	3.75	-10
Bm1_07885	109.0	0.95	48.5	5	0	1	0	0	0	0	1	3	2	0	0	0	0	0	0	0	na	-0.79	-10
Bm1_40540	117.0	1.07	45.8	7	1	0	0	1	0	0	0	0	5	0	0	0	0	0	0	0	na	-0.66	-10
Bm1_42620	70.5	0.84	60.1	4	0	1	1	0	0	0	2	0	2	0	1	0	0	0	1	0	na	0.30	-11
Bm1_33770	53.9	0.88	48.1	7	0	1	0	0	1	0	4	1	1	0	2	0	0	1	2	0	na	-0.88	-11
Bm1_36075	55.1	1.01	45.8	5	4	0	0	0	0	0	0	0	2	0	0	0	0	0	0	0	na	-1.28	-11
Bm1_49670	115.0	1.03	66.6	5	0	1	2	0	1	1	1	0	2	0	0	0	0	0	0	0	na	-1.17	-12
Bm1_48075	87.4	0.90	64.7	4	0	1	1	0	1	1	1	0	2	0	0	0	0	0	0	0	na	-1.04	-12
Bm1_04435	73.2	0.09	55.8	5	4	0	1	1	0	0	3	0	2	0	0	0	0	0	0	0	na	-0.18	-12
Bm1_55635	101.0	0.78	65.9	3	0	0	0	2	2	0	0	0	0	0	0	0	0	0	0	0	na	-0.72	-12
Bm1_57435	86.7	0.11	65.1	5	2	0	3	2	2	0	3	0	1	0	0	0	0	0	0	0	na	-0.52	-13
Bm1_14750	89.4	2.35	57.4	5	0	2	0	0	0	0	0	0	3	0	5	1	2	2	3	1	na	-0.36	-13
Bm1_34560	55.5	0.98	49.3	4	0	2	0	0	0	0	0	0	2	0	3	0	1	0	2	1	na	-1.30	-13
Bm1_46380	88.2	1.00	52.0	4	0	1	1	0	0	1	0	0	3	0	0	0	0	0	0	0	na	0.30	-13
Bm1_46795	92.4	0.83	70.5	5	0	2	1	0	1	3	1	0	1	0	1	0	1	0	0	0	na	-1.10	-14
Bm1_46160	119.0	0.94	66.6	5	1	2	0	0	0	0	2	0	2	0	0	0	0	0	0	0	na	-0.63	-14
Bm1_49750	80.9	0.66	69.7	4	2	1	1	0	0	0	1	0	2	0	1	0	0	0	2	0	na	0.19	-14
Bm1_47525	147.0	0.52	70.1	3	0	2	0	0	0	0	1	0	2	0	0	0	0	0	0	0	na	-0.31	-15
Bm1_12585	96.3	1.04	44.7	8	0	0	0	0	0	1	0	0	7	0	0	0	0	0	0	0	na	-0.49	-15
Bm1_37955	169.0	0.94	70.5	3	0	0	0	0	0	1	0	1	2	0	0	0	0	0	0	0	na	-0.22	-15
Bm1_18760	49.7	0.69	49.3	2	0	1	0	0	0	0	0	0	1	0	1	0	1	0	0	0	na	0.26	-15
Bm1_02155	169.0	0.31	59.7	4	0	1	1	2	0	0	0	0	2	0	2	0	1	0	1	0	na	0.03	-15
Bm1_01935	87.8	1.11	57.0	7	0	2	0	0	0	0	1	0	5	0	1	0	0	0	1	0	na	-0.43	-16
Bm1_03840	68.6	1.24	54.7	6	1	2	0	0	0	2	0	0	3	0	1	0	1	0	0	0	na	-1.19	-16
Bm1_37810	87.4	1.03	55.5	6	4	0	0	0	0	0	0	0	3	0	0	0	0	0	0	0	na	-0.98	-17
Bm1_16340	72.4	0.78	53.9	8	2	4	0	0	0	2	3	1	2	0	0	0	0	0	0	0	na	-0.86	-17
Bm1_27220	130.0	1.22	71.2	7	2	0	0	0	0	6	0	0	2	0	0	0	0	0	0	0	na	-0.64	-17
Bm1_14345	56.2	1.53	48.5	4	0	0	2	0	0	0	0	0	2	0	0	0	0	0	0	0	na	-0.48	-18
Bm1_48760	47.0	0.98	54.3	3	0	2	0	0	0	0	0	1	1	0	0	0	0	0	0	0	na	-0.50	-18
Bm1_37370	346.0	0.35	68.2	8	0	1	6	4	0	0	0	0	4	0	0	0	0	0	0	0	na	-0.73	-18
Bm1_02675	75.1	1.03	48.1	4	1	0	0	0	0	0	0	0	3	0	0	0	0	0	0	0	na	-0.43	-18
Bm1_02520	93.6	0.77	53.9	5	2	0	1	0	0	0	0	0	4	0	0	0	0	0	0	0	na	-1.35	-18

Bm1_56915	0.0	0.14	59.7	4	3	1	0	1	0	1	0	0	1	0	0	0	0	0	0	0	na	-0.28	-18
Bm1_41180	100.0	1.05	60.5	12	2	2	0	0	1	1	5	0	5	0	1	0	1	0	0	0	na	-0.65	-19
Bm1_10195	61.6	0.26	46.2	4	0	0	1	1	0	0	1	0	3	0	0	0	0	0	0	0	na	-0.34	-19
Bm1_37250	70.9	0.26	67.8	3	2	0	0	0	0	0	1	0	1	0	0	0	0	0	0	0	na	-0.49	-19
Bm1_41995	101.0	0.79	58.9	13	3	2	1	2	0	0	1	3	7	0	1	0	0	0	1	0	na	-1.92	-20
Bm1_44295	112.0	1.00	67.0	5	1	0	0	2	0	0	0	0	2	0	1	0	1	0	0	0	na	-0.79	-20
Bm1_48330	0.0	0.29	46.2	3	1	0	0	0	0	0	0	0	2	0	1	0	0	0	1	0	na	-0.47	-20
Bm1_45185	109.0	0.67	69.3	9	1	2	0	0	1	3	6	0	2	0	4	0	1	1	3	1	na	-0.52	-20
Bm1_44035	69.7	0.87	61.6	4	1	0	0	1	0	0	0	0	3	0	1	0	0	0	1	0	na	-0.77	-21
Bm1_28490	102.0	0.86	55.1	4	1	0	0	0	0	0	0	0	3	0	0	0	0	0	0	0	na	-1.27	-21
Bm1_26345	128.0	0.82	55.8	5	0	1	0	0	0	0	0	1	4	0	0	0	0	0	0	0	na	-0.17	-22
Bm1_09495	135.0	1.12	72.0	3	1	0	0	0	0	0	0	0	2	0	0	0	0	0	0	0	na	-0.18	-22
Bm1_17305	51.6	0.16	45.8	8	1	2	1	2	0	1	3	3	2	0	0	0	0	0	0	0	na	-0.80	-22
Bm1_25285	149.0	0.63	51.2	7	0	0	0	0	0	0	0	0	6	1	0	0	0	0	0	0	na	-0.52	-22
Bm1_17120	102.0	1.21	57.8	5	1	0	0	0	0	0	0	0	4	0	0	0	0	0	0	0	na	-1.17	-22
Bm1_54140	62.0	0.31	46.2	10	1	4	2	4	0	0	3	1	4	0	0	0	0	0	0	0	na	-1.08	-22
Bm1_19655	125.0	1.03	72.4	5	1	1	0	0	0	0	1	0	3	0	0	0	0	0	0	0	na	-0.68	-23
Bm1_42395	99.0	0.86	51.6	8	0	1	0	0	0	0	0	1	7	0	0	0	0	0	0	0	na	-0.46	-23
Bm1_32145	131.0	0.55	72.4	5	0	0	5	1	1	0	0	0	1	0	0	0	0	0	0	0	na	-0.83	-23
Bm1_31210	56.6	1.75	67.0	6	4	1	0	1	0	0	2	0	3	0	0	0	0	0	0	0	na	-0.47	-23
Bm1_27030	69.3	0.02	53.9	11	3	2	9	4	1	3	7	0	3	0	0	0	0	0	0	0	na	-0.44	-23
Bm1_27570	144.0	0.09	55.5	9	11	1	3	0	0	1	1	0	1	0	0	0	0	0	0	0	na	-0.88	-23
Bm1_26605	134.0	1.06	65.5	6	1	0	0	0	0	0	0	0	5	0	0	0	0	0	0	0	na	-0.31	-24
Bm1_46495	159.0	0.37	57.0	4	0	0	1	0	0	1	0	0	1	0	0	0	0	0	0	0	na	-0.38	-24
Bm1_04785	98.2	0.88	52.8	5	0	0	0	0	0	0	0	1	4	0	0	0	0	0	0	0	na	-0.49	-25
Bm1_01560	118.0	0.62	49.3	4	0	0	0	0	0	0	0	1	3	0	0	0	0	0	0	0	na	-0.33	-25
Bm1_49645	62.8	0.89	58.9	3	0	0	0	0	0	0	1	0	2	0	0	0	0	0	0	0	na	-0.30	-25
Bm1_45665	97.4	0.69	65.5	5	2	0	1	0	0	0	1	0	4	0	0	0	0	0	0	0	na	-0.21	-25
Bm1_30230	84.3	1.12	61.6	5	1	0	0	0	0	1	0	0	3	0	0	0	0	0	0	0	na	-0.88	-26
Bm1_45060	105.0	0.72	55.1	5	1	0	0	0	0	0	0	0	5	0	0	0	0	0	0	0	na	-0.36	-26
Bm1_15510	200.0	0.35	55.5	4	0	1	0	0	0	0	0	0	3	0	0	0	0	0	0	0	na	-0.36	-26
Bm1_05930	242.0	0.14	69.7	6	0	2	0	2	0	2	4	0	1	0	0	0	0	0	0	0	na	-0.69	-26
Bm1_47605	145.0	0.46	49.3	3	0	0	0	0	0	0	0	1	2	0	0	0	0	0	0	0	na	-0.10	-27
Bm1_00645	211.0	0.08	65.9	6	0	2	0	2	0	2	4	0	1	0	0	0	0	0	0	0	na	-0.46	-27
Bm1_43030	231.0	0.49	61.6	4	0	0	0	0	0	0	0	1	3	0	0	0	0	0	0	0	na	-0.20	-27
Bm1_56230	231.0	0.61	66.2	3	0	0	0	0	0	0	0	1	2	0	0	0	0	0	0	0	na	0.31	-27
Bm1_34260	47.0	1.10	53.1	4	1	0	0	0	0	0	0	0	3	0	0	0	0	0	0	0	na	-1.15	-27
Bm1_41220	80.5	0.77	56.2	5	0	1	0	0	1	0	0	0	4	0	0	0	0	0	0	0	na	-0.64	-28
Bm1_34445	91.7	1.05	68.6	7	0	1	0	0	0	0	0	1	5	0	2	0	1	0	2	0	na	-1.01	-28
Bm1_39315	94.7	0.80	61.6	11	4	2	0	0	0	1	1	0	5	0	0	0	0	0	0	0	na	-1.09	-28
Bm1_33410	57.8	0.98	52.4	3	0	0	0	0	0	0	0	1	2	0	0	0	0	0	0	0	na	-0.83	-29
Bm1_11825	264.0	0.28	65.9	5	1	0	1	0	0	0	1	1	2	0	0	0	0	0	0	0	na	0.16	-30
Bm1_15840	90.5	0.42	67.0	5	0	3	0	0	0	0	2	0	1	0	0	0	0	0	0	0	na	-1.56	-30

Bm1_13965	54.3	0.62	46.2	7	0	0	0	0	0	0	0	1	6	0	0	0	0	0	0	0	na	-1.00	-30
Bm1_57145	166.0	0.08	53.1	10	2	1	0	1	0	3	4	0	5	0	0	0	0	0	0	0	na	-0.89	-30
Bm1_17070	111.0	0.72	72.4	5	0	0	1	1	0	0	2	0	3	0	0	0	0	0	0	0	na	-0.04	-30
Bm1_47600	233.0	0.39	61.6	3	0	0	0	0	0	0	0	1	2	0	0	0	0	0	0	0	na	-0.25	-31
Bm1_19390	122.0	0.22	47.0	12	0	0	2	0	0	0	0	2	7	0	0	0	0	0	0	0	na	-0.32	-31
Bm1_56290	188.0	0.25	74.3	9	11	1	3	0	0	1	1	0	1	0	0	0	0	0	0	0	na	-0.77	-32
Bm1_11340	88.2	0.12	63.2	3	1	0	0	1	0	0	0	0	1	0	0	0	0	0	0	0	na	-0.54	-32
Bm1_38435	134.0	0.18	55.5	12	0	0	0	0	0	0	0	0	11	1	1	0	0	0	1	0	na	-0.61	-32
Bm1_03370	57.4	0.37	54.3	4	1	0	0	0	0	0	0	0	3	0	1	1	0	0	0	0	na	-1.29	-33
Bm1_28035	81.3	0.97	67.0	6	0	2	0	0	1	0	0	0	4	0	0	0	0	0	0	0	na	-1.85	-33
Bm1_23370	133.0	0.86	70.1	13	0	2	2	0	1	0	0	0	7	4	0	0	0	0	0	0	na	-0.11	-33
Bm1_47300	98.6	0.78	69.3	8	0	0	0	0	0	0	0	0	7	1	2	0	0	0	3	1	na	-0.47	-33
Bm1_06640	83.2	0.99	64.3	6	0	1	0	0	0	0	0	0	5	0	0	0	0	0	0	0	na	-0.94	-33
Bm1_33205	143.0	0.59	62.8	12	0	0	0	0	0	0	0	0	9	3	0	0	0	0	0	0	na	-0.80	-33
Bm1_27455	62.0	1.24	60.1	9	1	0	0	0	0	0	0	3	5	0	0	0	0	0	0	0	na	-0.59	-34
Bm1_22440	89.4	0.70	70.1	3	1	0	0	0	0	0	0	0	3	0	0	0	0	0	0	0	na	-0.25	-34
Bm1_52815	72.4	0.45	59.3	5	0	0	0	0	0	2	0	0	4	0	0	0	0	0	0	0	na	-0.24	-35
Bm1_06130	171.0	0.04	52.4	23	0	9	0	0	0	0	4	0	14	0	1	0	2	0	0	0	na	-0.40	-36
Bm1_06130	169.0	0.04	52.4	23	0	9	0	0	0	0	4	0	14	0	1	0	1	0	0	0	na	-0.38	-36
Bm1_29715	49.7	0.98	67.8	7	0	0	0	0	1	3	1	0	4	0	0	0	0	0	0	0	na	-0.46	-37
Bm1_18195	84.3	0.21	48.9	15	0	0	0	0	0	0	0	0	13	2	0	0	0	0	0	0	na	-0.20	-37
Bm1_37240	80.9	0.83	63.9	3	0	0	0	0	0	0	0	1	2	0	0	0	0	0	0	0	na	-0.24	-37
Bm1_21825	72.4	0.84	67.8	5	1	0	0	0	0	0	0	1	4	0	0	0	0	0	0	0	na	-0.65	-38
Bm1_56600	69.7	1.10	64.7	8	0	0	0	0	0	1	0	0	7	0	0	0	0	0	0	0	na	-0.05	-38
Bm1_55355	170.0	0.37	65.9	6	1	0	0	0	0	0	0	0	5	0	0	0	0	0	0	0	na	-0.23	-39
Bm1_21530	0.0	1.11	48.9	5	0	0	0	0	0	0	0	1	4	0	0	0	0	0	0	0	na	-0.70	-39
Bm1_47345	72.4	0.25	70.9	10	2	1	0	0	0	2	6	0	4	0	2	0	0	1	0	1	na	-0.21	-39
Bm1_38400	76.6	0.49	60.8	7	0	0	0	0	0	0	1	0	6	0	0	0	0	0	0	0	na	-0.49	-39
Bm1_13715	113.0	0.12	56.6	5	1	0	0	0	0	0	0	0	4	0	0	0	0	0	0	0	na	-0.35	-39
Bm1_01555	164.0	0.48	68.2	4	0	0	0	0	0	0	0	1	3	0	0	0	0	0	0	0	na	-0.16	-39
Bm1_56045	238.0	0.07	64.7	23	0	9	0	0	0	0	4	0	14	0	1	0	0	0	2	0	na	-0.57	-40
Bm1_17360	180.0	0.14	58.2	6	0	0	0	1	0	0	0	0	5	0	0	0	0	0	0	0	na	-0.40	-40
Bm1_11715	107.0	0.12	57.4	9	1	0	0	0	0	0	1	0	6	0	0	0	0	0	0	0	na	-0.37	-41
Bm1_20785	120.0	0.38	67.8	5	1	0	0	0	0	0	0	0	4	0	0	0	0	0	0	0	na	-0.53	-42
Bm1_16705	61.2	0.63	63.5	7	1	0	0	0	0	0	1	0	6	0	0	0	0	0	0	0	na	0.14	-42
Bm1_02455	56.2	0.75	65.5	5	0	0	0	0	1	0	0	0	4	0	0	0	0	0	0	0	na	-1.00	-42
Bm1_05345	89.0	0.11	65.5	6	0	1	0	0	0	0	1	0	5	0	1	0	0	0	0	1	na	-0.28	-42
Bm1_46675	105.0	0.49	67.0	4	0	0	0	0	0	0	0	1	3	0	0	0	0	0	0	0	na	-0.31	-43
Bm1_43275	85.1	0.58	72.0	4	1	0	0	0	0	0	0	0	3	0	0	0	0	0	0	0	na	-0.15	-43
Bm1_12300	147.0	0.07	65.5	5	1	0	1	0	0	0	0	0	4	0	0	0	0	0	0	0	na	-0.50	-43
Bm1_23135	94.0	0.12	56.6	10	0	0	0	0	0	0	0	1	9	0	0	0	0	0	0	0	na	-0.05	-45
Bm1_20840	95.5	0.37	70.1	6	0	0	0	2	0	0	0	0	4	0	0	0	0	0	0	0	na	-0.48	-46
Bm1_26685	186.0	0.05	62.4	6	0	0	0	1	0	0	0	0	5	0	0	0	0	0	0	0	na	-0.51	-47

Bm1_54070	213.0	0.09	67.0	6	0	0	0	1	0	0	0	0	5	0	0	0	0	0	0	0	na	-0.03	-50
Bm1_02325	182.0	0.05	67.0	29	0	8	0	0	0	0	0	0	19	1	0	0	0	0	0	0	na	-0.39	-51
Bm1_04865	0.0	1.20	68.9	10	4	0	0	0	0	0	0	0	9	0	0	0	0	0	0	0	na	-0.34	-52
		min_value																					-52
		max_value																					275
